# Mouse genotypes drive the liver and adrenal gland clocks

**DOI:** 10.1038/srep31955

**Published:** 2016-08-18

**Authors:** Rok Košir, Uršula Prosenc Zmrzljak, Anja Korenčič, Peter Juvan, Jure Ačimovič, Damjana Rozman

**Affiliations:** 1 Center for Functional Genomics and Bio-Chips, Institute of Biochemistry, Faculty of Medicine, University of Ljubljana, Zaloska cesta 4, Ljubljana, Slovenia; 2 Institute of Biochemistry, Faculty of Medicine, University of Ljubljana, Zaloska cesta 4, Ljubljana, Slovenia

## Abstract

Circadian rhythms regulate a plethora of physiological processes. Perturbations of the rhythm can result in pathologies which are frequently studied in inbred mouse strains. We show that the genotype of mouse lines defines the circadian gene expression patterns. Expression of majority of core clock and output metabolic genes are phase delayed in the C56BL/6J line compared to 129S2 in the adrenal glands and the liver. Circadian amplitudes are generally higher in the 129S2 line. Experiments in dark – dark (DD) and light – dark conditions (LD), exome sequencing and data mining proposed that mouse lines differ in single nucleotide variants in the binding regions of clock related transcription factors in open chromatin regions. A possible mechanisms of differential circadian expression could be the entrainment and transmission of the light signal to peripheral organs. This is supported by the genotype effect in adrenal glands that is largest under LD, and by the high number of single nucleotide variants in the Receptor, Kinase and G-protein coupled receptor Panther molecular function categories. Different phenotypes of the two mouse lines and changed amino acid sequence of the *Period 2* protein possibly contribute further to the observed differences in circadian gene expression.

The majority of organisms on Earth have evolved a robust inner body circadian (*circa* = approximately, *dian* = day) clock that ticks away in most of their cells. It controls physiological, behavioural and cellular processes, ranging from body temperature, sleep/wake and feeding rhythms to metabolism and hormone secretion^1^. In mammals, the hierarchical structure of the circadian system necessitates that the master clock located in the suprachiasmatic nucleus (SCN) of the hypothalamus not only synchronises peripheral clocks but also entrains its own clock to the day/night cycle^1^. While the SCN clock seems to entrain to the outside world primarily through photic information *via* the retina and the retinohypothalamic tract, peripheral tissues entrain to cues through direct (humoral and neural connections) and indirect SCN signals (rest/activity cycles, feeding time, body temperature)^2^,^3^. Despite differences in the anatomical structure of the circadian systems, the molecular mechanisms generating oscillations are identical in all cells and consist of a transcriptional-translational feedback loop. The positive part of this loop, composed of a BMAL1 dimer with either CLOCK or NPAS2, enables E-box mediated transcriptional activation of core clock and clock controlled genes (CCG). 43% of all protein coding genes show circadian rhythms in transcription somewhere in the body, largely in an organ-specific manner^4^,^5^.

Inbred mouse strains are invaluable tools towards understanding the molecular biology and influence of rhythm perturbations on disease development *in vivo*^2^,^6^ Especially for mutant strains, conducting research on one mouse strain alone may lead to missing diverse phenotypes^7^, as had been shown for the development of metabolic syndrome in *Clock* mutant mice^8^,^9^. Other aspects of circadian physiology are also affected by genotype including free-running activity rhythms^10^,^11^, feeding cycles^12^ and phase-shifting effects^13^. Among rare understood molecular effects are mutations in the *Hiomt* gene^14^ that result in different melatonin production in mouse strains. Recent whole genome sequencing projects exposed a large number of structural and single nucleotide variants between the inbred mouse strains^15^. Mouse knock-out models of core clock genes were frequently derived by gene targeting of embryonic stem cells of 129 mouse strains^16^,^17^,^18^ and the initial analyses performed on mice with a mixed genetic background, where results can be confounded by background effects^19^.

We discovered crucial changes in circadian gene expression in the C57BL/6JOlaHsd and the 129S2/SvPas × C57BL/J6 strains in peripheral organs. Whole exome sequencing and *in silico* analyses were applied to expose the nucleotide variants which could contribute importantly to the observed differences in the circadian or diurnal patterns of genes expression (Fig. 1).

## Results

### Circadian gene expression in liver and adrenal glands of C57BL/6 and 129S2 mouse lines

We evaluated gene expression patterns of 10 core clock and 15 metabolic genes collected under DD and LD conditions (Supplementary Figs S1 and S2). In liver we screened expression of the genes from cholesterol synthesis (*Cyp51, Cyp7a1, Cyp8b*, *Por, Hmgcr*) and regulators of metabolism (*Ppara, Ppargc1a, Car, Rxr*). In adrenal glands we focused on steroid metabolism (*Cyp51, Cyp17a1, Cyp11a1, Cyp11b2, Cyp21a1*). A detailed list is in Supplementary Table S1. Time series gene expression data were analysed by cosinor analysis to determine the presence/absence of circadian oscillation (Table 1, Supplementary Table S2). Under LD conditions all core clock genes displayed a clear diurnal rhythm in liver and adrenal glands of both mouse lines (Table 1). Under DD, *Dec2* and *Cry2* in the liver and *Per1* in adrenal glands are arrhythmic in C57BL/6 (Supplementary Table S2). Metabolic genes are significantly more affected by genotype since many are circadian (in DD) and diurnal (in LD) in one but not the other mouse line under the same condition and tissue (Supplementary Table S2). Overall, a higher number of investigated genes displays circadian or diurnal expression in the 129S2 genotype (87%) compared to the C57BL/6J (76%) (Table 1).

**Figure 2:**
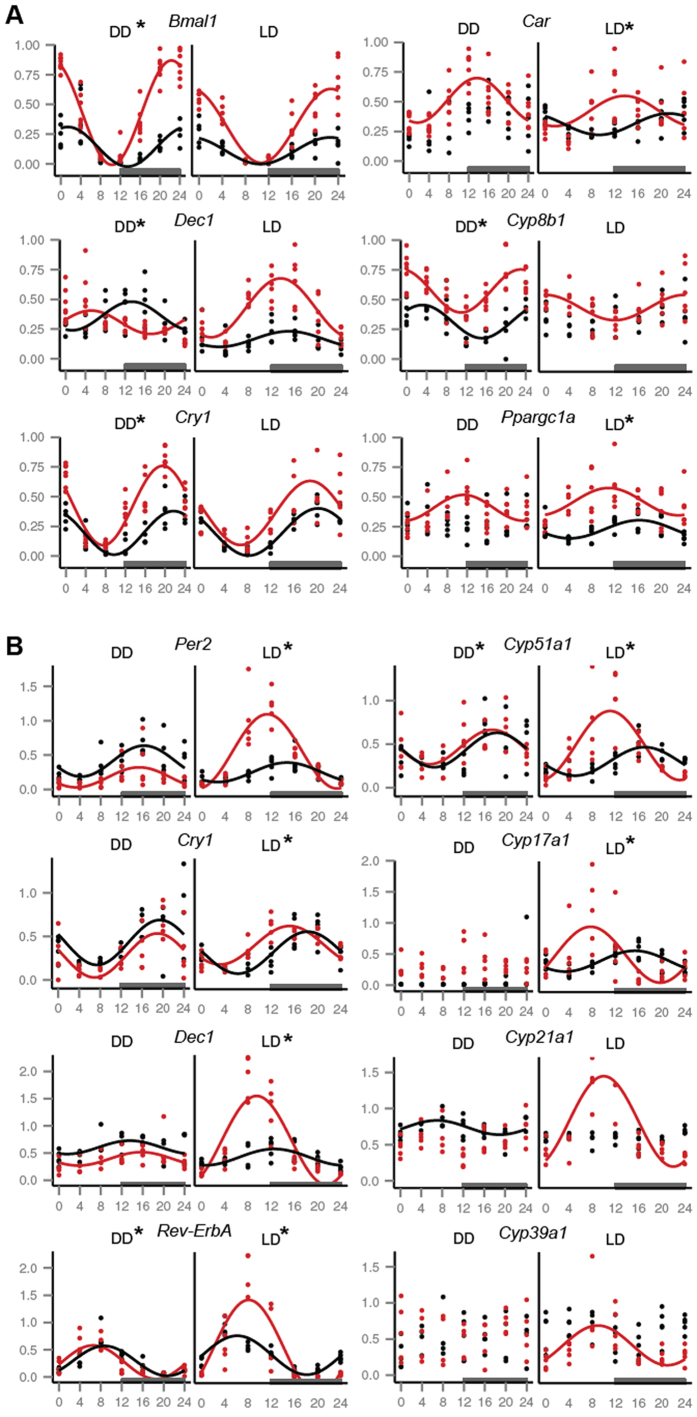
Circadian gene expression patterns. Differences in phase of core clock and metabolic genes were discovered in mouse liver –(**A**) and adrenal glands – (**B**). Each dot represents one mouse sample of either 129S2 (red) or C57BL/6J (black) strain. Curves confirm the presence of a circadian (DD) or diurnal (LD) rhythm based on the cosinor analysis with a period of 24 h. If no curve is present a gene did not show a statistically significant (p < 0.01) circadian expression pattern. Mice were sampled every 4 hours during a 24 h period in either DD – dark-dark conditions or LD – light-dark conditions. The horizontal axis represents circadian or diurnal time of sampling; CT0 corresponds to 7 am. The dark grey rectangle indicates the subjective night and night in DD and LD conditions respectively.

**Figure 3:**
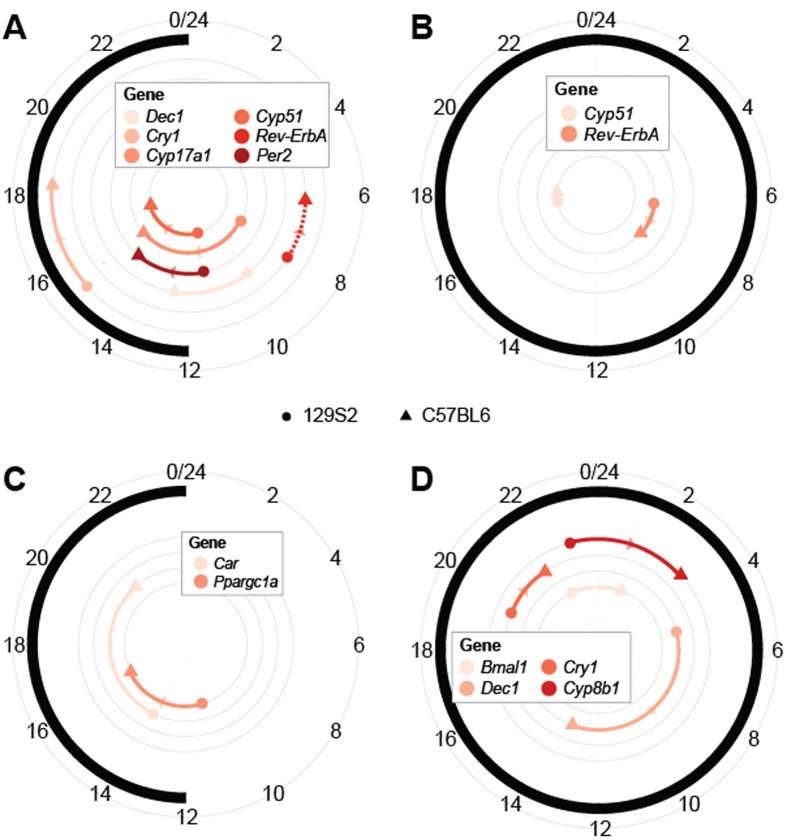
Genotype effect of the phase of gene expression. The 24 h day is represented in the form of a circular diagram with each gene shown on a separate concentric circle. The time of peak expression (phase) for each gene (colour coded) is marked with a circle (129S2) or triangle (C57BL/6J) depending on mouse genotype. Coloured solid and dashed lines represent genes where the C57BL/6J genotype had a phase delay or phase advance respectively. The solid black line shows the time of lights out (darkness) under LD (A, C) or DD (B, D) conditions. A and B represent adrenal glands; C and D represent liver. All genes with the exception of *Rev-ErbA* show a phase delay (expression peak at a later CT) in the C57BL/6J strain in all conditions and tissues. *Rev-ErbA* in adrenal glands under LD conditions (A – dashed line) shows a phase advance. The largest effect of genotype was seen under LD conditions in adrenal glands (A).

In Table 2 and Supplementary Table S3 we compared circadian phases and amplitudes of gene expression in both mouse lines. Based on mathematical models of circadian rhythms constructed from *in vivo* data we regarded phases as more important since changes in phases lead to large perturbations of the whole circadian system^20^. The comparative analysis shows that phases of several core clock genes are affected by the genotype, including *Bmal1, Dec1* and *Cry1* in the liver, and *Per2, Dec1, Cry1* and *Rev-ErbA* in adrenal glands (Figs 2 and 3). Interestingly, whereas in liver the circadian phases of gene expression differ between mouse lines in complete darkness (DD) (Figs 2A and 3D), the differences in phases in adrenal glands are seen primarily under LD conditions (Figs 2B and 3B). As shown in Fig. 3, the peak expression of core clock genes is phase delayed in C57BL/6J compared to the 129S2 with the exception of *Rev-ErbA* in adrenal glands (Fig. 3A).

Generally, metabolic genes were more variable concerning the presence**/** absence of circadian expression which makes comparisons of some phases and amplitudes impossible. More importantly, in both light conditions and tissues all metabolic genes for which circadian or diurnal rhythmicity was confirmed showed a phase delay in the C57BL/6J genotype (Figs 2 and 3). The phase delay is on average 2.8 h for core clock genes and 6.1 h for metabolic genes for both tissues and light conditions.

Similarly, as for the phases, genotype dependent rules were observed also for amplitudes. For the liver metabolic genes, the amplitudes were less affected by the genotype compared to core clock genes (Supplementary Table 3) while in adrenal glands the amplitudes were affected only when light had been present (LD). Generally, amplitudes were in most cases higher in the 129S2 genotype (*Cry1* and *Cyp11b1* are exceptions) (Table 2, Supplementary Table S3), suggesting differences in entrainment and in transmission of the light signal to the adrenal gland between C57BL/6J and 129S2 lines.

### Exome sequencing: functional characterization of SNVs and genome variations in differentially expressed circadian genes

**Figure 4:**
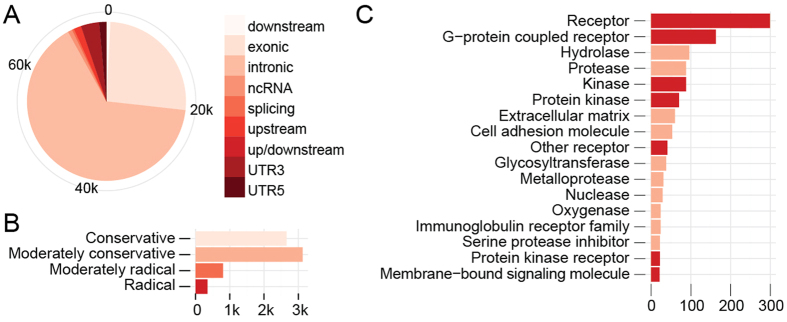
Whole exome sequencing analysis. An overview of single nucleotide variants discovered to be different between strains used in our analysis (**A)** The pie chart shows the relative abundance of SNVs found at different genomic locations: the majority were discovered in intronic regions with a little over one quarter in exonic regions. (**B)** The bar chart shows the number of non-synonymous variants found in each class based on the Grantham Matrix Score. The majority of variants were classified as conservative and moderately conservative. (**C**) Results of DAVID analysis of genes having nonsynonymous variants within their exome region. Dark red represents gene categories also involved in circadian entrainment pathways.

In effort to reveal molecular causes for the phase delays and other variations in circadian or diurnal expression profiles whole exome sequencing has been performed by Illumina HiSeq 2000 platform to about 50-fold coverage using the 100-bp paired-end reads (Supplementary Table S4, S5). A total of 90,994 SNVs (85,224 in the 129S2 and 5,770 in the C57BL/6J lines) were discovered in exomes when compared to the NCBI reference mouse line (NCBIM37/mm9). The 3,426 SNVs that were identical in both mouse lines were removed from further analysis. As shown in Table 3 the majority of SNVs lie in the non-coding genome including intronic (57.99%), intergenic (10.50%) and UTR (4.73%) regions. Approximately 20,000 SNVs (23.16%) are within exon regions (Fig. 4A). These nucleotide variations can affect the resulting phenotype, especially 6,938 (36.5%) variants causing nonsynonymous substitutions including loss or gain of stop codons.

These variants were distributed in 2,553 genes of which the majority (86%) carried multiple (up to 4) variants. The rest (356 genes) showed a higher number of nonsynonymous SNVs. To predict which of the variants could have deleterious effect, Grantham Matrix Score (GMS) was determined for each nonsynonymous SNV. The majority of nonsynonymous SNVs belong to conservative (38.31%) and moderately conservative groups (45.07%) while the most interesting are the 5% and 11.6% SNVs from the radical and moderately radical groups (Fig. 4B). 287 genes from the radical group were annotated: significant association was found with Panther molecular function categories including Receptor (p < 5.7e-4), Kinase (p < 1.9e-2) and G-protein coupled receptor (p < 2.4e-2) (Fig. 4C). DAVID Analysis of SNVs that result in gain or loss of stop codons also showed significant association with Panther molecular function categories: Receptor (p < 4.7e-2) and G-protein coupled receptor (p < 6.5e-2). 42 out of 80 variants discovered by exome sequencing were included in DAVID analysis. The remaining SNVs were removed after manual inspection with Variant Effect Predictor (VEP) since Ensembl had shown them not to be true gain or loss of stop codons variants (Supplementary Table S6).

Since the biggest differences in gene expression was detected in adrenal glands in LD conditions, we screened the literature for known genes and pathways involved in transmission of light information from the retina to the adrenal gland. A list of over 200 genes was created including genes from light reception, SCN light transduction, SCN neuropeptides, steroid metabolism and regulation, etc. (Supplementary Table S7). These genes were analyzed for the presence of SNVs and 74 genes were identified. The most interesting were melanopsin (*Opn4*), a G-protein coupled receptor and a photopigment that is important for proper circadian entrainment^21^; vasoactive intestinal peptide receptor (*Vipr2*) important for synchronization of pacemaker neurons in the SCN^22^; Neuronal PAS domain-containing protein 2 (*Npas2*), a functional substitute of the CLOCK protein in the SCN^23^ and the steroidogenic factor 1 (*Sf1*), essential for expression control of most steroidogenic genes^24^.

40 SNVs were discovered in intronic, UTR and exonic regions of *Opn4* with 4 non-synonymous SNVs located in the C-terminal cytoplasmic region with several amino acids that can undergo phosphorylation^25^,^26^. Three SNVs (c.C1504T:p.P502S, c.G1496C:p.C499S and c.A1402T:p.T468S) induce a conversion of the original amino acid to serine in the 129S2 mouse line, increasing potential phosphorylation sites and possibly affecting signaling. In humans mutations of the *Opn4* gene result in seasonal affective disorder^27^.

*Vipr2* gene contains 25 intronic and 1 nonsynonymous exonic SNV (c.G1309C:p.A397P). The later induces conversion of alanine to proline in C terminal region, potentially changing the secondary structure. *Vipr2*^−/−^ mice lack a normal circadian rest/activity behavior and have no circadian expression of the core clock genes *Per1, Per2*, and *Cry1* in the SCN. They also failed to show acute induction of *Per1* and *2* by nocturnal illumination^28^.

20 SNVs were located in *Npas2*, one in the 5‘ UTR and 4 in exons, two of these nonsynonymous (c.T1938C:p.L481P, c.A2189G:p.T562A). *Npas2* is expressed in neural tissues and is critical for adaptation to restricted feeding^29^.

*Sf-1* (*Nr5a1*) contains 2 SNVs, one is nonsynonymous (c.G715T:p.A172S) and lies near the phosphorylation site (p.203) which is important for maximal SF1-mediated transcription^30^. The newly formed serine can also be phosphorylated, suggesting that this SNV could affect the protein activity in the 129S2 mouse line. *Sf-1* expression requires E-box element located in the promoter region^31^. SF-1 regulates *Cyp11a1* and *Cyp21a1* expression that show different amplitudes in LD in adrenal glands (Supplementary Tables S2 and S3). Described SNVs and others mentioned in Discussion could affect the gene expression in adrenal gland.

Differences in the liver expression patterns could be influenced also by metabolic signals (majority of differences between strains were detected in DD). Literature screen and SNV analysis identified some candidate genes that could potentially serve as sensors of hepatocyte external stimuli, but only *Ppargc1a* and *Npas2* harbor nonsynonymous SNVs in coding regions. *Ppargc1a* encodes a transcriptional coactivator (PGC1a) that coordinates gene expression of mitochondrial biogenesis, respiration, hepatic gluconeogenesis and is considered as one of the most important integrators of external stimuli. SNV analysis revealed a change in amino acid sequence (c.G2163A:p.R675H), that is important for interaction with other transcription factors.

Even if both mouse lines had the access to the same food *ad libitum*, the phenotypic differences in food and water consumption could participate to hepatic circadian expression differences^32^. The hepatic clock can easily be re-set with the restricted feeding (RF). According to the literature, one of the most described signaling pathways that could trigger hepatic re-setting is the insulin signalling cascade^33^. We chose 50 genes involved in the insulin signalling^34^ and checked for the presence of SNVs (Supplementary Table S8). The insulin receptor gene *Insr* contains only synonymous SNVs (6 exonic, 1 intergenic, 4 in 3UTR, 14 intronic) so it is difficult to speculate on their function. A nonsynonymous SNV (c.T590G:p.V197G) was found in *Irs2* (insulin receptor substrate protein) in the PTBi domain which is important for the phosphorylation status dependent peptide binding. Since the valine to glycine change is near the phosphoinositide binding site the activity of the IRS2 might be modified. Non-synonymous SNVs reside in other feeding related genes: in protein tyrosine phosphatase gene *Ptpn14* which is involved in mediation of insulin sensitivity by de-phosphorylating tyrosine residues in the insulin receptor and reducing its activity. Among 21 SNVs one is nonsynonymous (c.A2660G.p.H887R) in the protein region with yet unknown function. SNVs are found also in other genes: in the gene c-Jun-N-terminal kinase *Mapk10* (c.C1370T:p.A457V) at the site with the unknown function, in *Cdc42bpa* (c.G12562A:p.R521Q), and in *Cdc42se2* (c.C128T:p.S43F) near the GTPase interaction site. CDC42 binds to p85a PI3K isoform and modulates insulin sensitivity of the cell. Nine SNVs are found in *Cap1* gene involved in the CAP-Cbl pathway collaborating with the PI3K pathway in stimulation of GLUT4 translocation. One SNV (c.T724C:p.S242P) is located in the region with the trypsin-like protease activity.

At the molecular level, the circadian system is maintained by intertwining feedback loops, so we wondered whether the SNVs from the exome sequencing lie in our genes of interest and could therefore affect the differential circadian patterns in mouse lines. 77 SNVs were discovered in core clock (*Bmal1*, *Per1*, *Per2*, *Dbp*) and metabolic genes (*Cyp21a1*, *Cyp17a1*, *Cyp51*, *Ppargc1a*, *Car* and *Ppara*) (Table 4 and Supplementary Table S9) with more than 75% in *Bmal1, Per2, Ppargc1a* and *Car* (in average 14 SNVs per gene). Nonsynonymous mutations reside in the core clock gene *Per2* and in transcriptional coactivator *Ppargc1*, with GMS scores of 58 and 29, respectively. In *Per2* serine is changed to threonine at position 172 (G515C:p.S172T) just before the PAS 1 domain important for the dimerization. The amino acid substitutions for the *Ppargc1a* gene are described above.

### Genome variations in binding sites of core clock proteins

**Figure 5:**
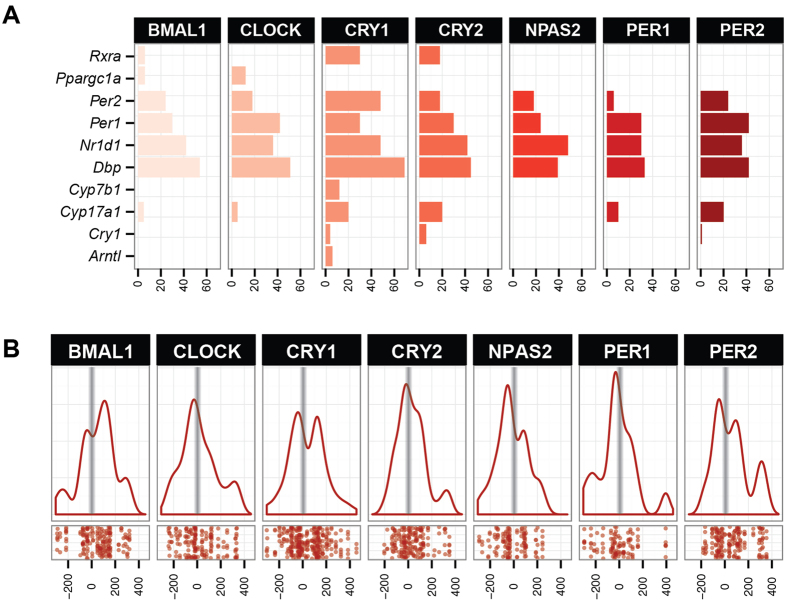
*In silico* analysis of promoter regions. We determined the presence of SNPs in core clock protein binding sites in genes whose expression was measured with RT-qPCR. Data for core clock binding sites were obtained from ChIP-Seq data from Koike *et al*.^21^; data for the presence of SNPs was taken from the Imputed Mouse SNP Resource^22^. Data on SNPs locations of six 129 strains was obtained from the database and used for analysis. (**A**) The bar chart represents the number of SNPs found in the binding site of core clock proteins in our genes of interest. (**B**) Red lines represent average densities of SNPs across all binding sites in different GOI for different core clock proteins. Data from Koike *et al*. was normalized, so that the peak of each binding region corresponds to 0 in the graph below (and is marked with a grey vertical line). Positions of the SNPs are recalculated and displayed in base pare distance so each SNP lies relative to the peak center.

We evaluated promoters of genes of interest and checked for SNPs of clock protein binding sites. DNA binding sites of BMAL1, CRY1, CRY2, PER1, PER2, CLOCK and NPAS2 were taken from ChIP-Seq experiments performed by Koike *et al*.^35^. Since the majority of binding regions were outside the exome sequencing area, we applied data from the Imputed Mouse SNP resource^36^ that included six 129 lines: 129P1/ReJ, 129P3/J, 129S1SvlmJ, 129S6, 129T2/SvEmsJ and 129 × 1/SvJ, for which SNPs are determined compared to the C57Bl/6J reference genome. We searched for the presence of SNPs within clock protein binding regions (Supplementary Table S10). Figure 5A displays the number of SNPs per core clock protein binding region in genes of interest. Promoters of *Dbp, Rev-ErbA, Per1* and *Per2* have the largest number of SNPs. These genes (and some others) have also the highest number of core clock protein binding peaks according to Koike et. al^35^: *Dbp* 4.29 peaks per gene, *Cry2* 4.57, *Per1* 4.71, *Per2* 5.43, *Por* 5.44 and *Rev-ErbA* 7.14.

When determining the density of SNPs within each clock protein binding region, we compared it with the location of ChIP-Seq peaks (Fig. 5B). In most cases the location of ChIP-Seq peak coincides with a peak in SNP density so the differential regulation of circadian expression is expected.

To evaluate the SNP density in transcription factor binding regions compared to the rest of the DNA, we assessed it in the context of the DNase hypersensitive sites (DHS) form open chromatin regions^37^ (Supplementary Table S11). DHS regions where clock related proteins bind contain one SNP per 219.6 nucleotides in comparison to all standard DHS sites which contain one SNP per 224.8 nucleotides on average; the difference is statistically significant (p-value 0.045).

## Discussion

The relatively high degree of anatomic, physiological and genomic similarities between humans and mice (approx. 95% of genes are shared) have made mice important models for the study of various biomedical issues^6^,^38^. To eliminate misleading results due to genetic variability of mice, lines with the lowest degree of intra-strain variability are selected. This precluded us from seeing the effects of mouse genetic backgrounds on studied processes^7^.

**Figure 6:**
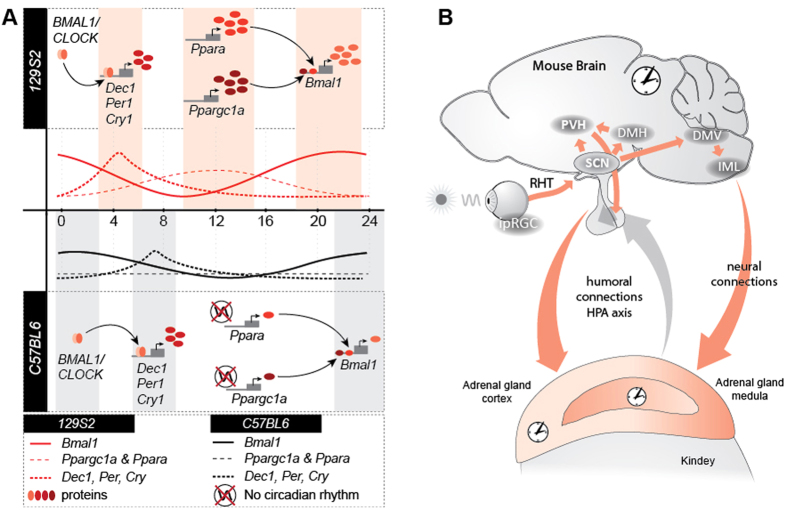
The effect of genotype on gene expression. Circadian (DD) or diurnal (LD) expression of core clock and metabolic genes was shown to be affected by mouse genetic background. A mechanism based on gene expression data only that could explain the differences observed in liver is proposed under A. (**A**) The noncircadian expression of *Ppargc1a* and *Ppara* in the C57BL/6J strain could lead to a reduced and phase delayed expression of *Bmal1* (confirmed by expression data) which in turn could lead to a reduced and delay expression of other core clock (confirmed for *Cry1* and *Bhlhe40*). (**B**) In adrenal glands the largest differences observed were under LD conditions, leading us to believe that entrainment pathways could be affected by mouse genotype. Several SNVs were discovered in genes known to be involved in circadian entrainment such as *Opn4*, *Vipr2* and *Npas2*. The picture depicts several neural pathways between different brain regions known to be involved in entrainment as well as hormonal connections important in adrenal regulation that could be affected by SNVs present between strains.

Here we show a clear genotype effect on circadian or diurnal gene expression, with differences in phases and amplitudes of core clock and metabolic genes in C57BL/6J and 129S2 mouse lines. In liver of the C57BL/6J genotype two core clock (*Cry2* and *Dec2*) and several metabolic genes (*Car, Cyp7a1, Pgc1a* and *Pparα*) lacked circadian expression under DD in contrast to the 129S2 genotype (Supplementary Table S2). While the absence of circadian expression in the two clock genes is not sufficient to abolish rhythmicity due to the redundancy of the clocks molecular network^39^, as shown by mouse knock-out models of *Cry2*^16^ and *Dec2*^40^, it could lead to changes in circadian expression of clock controlled genes^41^. This is supported by the absence of circadian rhythm in two important regulators of hepatic energy metabolism, *Pparα* and *Pgc1α.* Although expression of both genes is regulated by the clock, they also reciprocally regulate expression of *Bmal1* and in turn circadian rhythms (Fig. 6A)^42^,^43^. The lack of circadian peak of *Pparα* and *Pgc1α* in the C57BL/6J genotype could lead to reduced and delayed peak of *Bmal1* expression (Supplementary Figure S1, Supplementary Tables S2 and S3). This might also be the reason for the reduced amplitudes of *Per1, Cry1, Cry2, Dbp* and *Rev-ErbA* as well as the observed delayed peaks of *Dec1* and *Cry1* (Figs 2A, 3D and 6A, Supplementary Table S3). PPARα is a nuclear receptor that binds exogenous as well as endogenous substances. The availability of those ligands can alter the PPARα activity which is not necessary following the mRNA expression. Fatty acids (arachidonic, polyunsaturated FAs and their derivates) are among the most important ligands of PPARα^44^. In our experimental setup both mouse lines were kept under the same laboratory conditions and had the access to the same amount and type of food. However, the ingestion, absorption and metabolism of ligands can differ due to genetic background. Similar interpretation could be used also for the activity of PGC-1α which is a master integrator of external stimuli: AMPK phosphorylation and by SIRT1 regulated epigenetic status can alter PGC-1α activity according to the metabolic state of the organism (energy needs, cold, fasting, exercise etc)^45^.

The largest influence of genotype was, however, observed in adrenal glands under LD conditions. Here the majority of measured clock genes were differentially expressed between mouse lines either in amplitude or phase (Figs 2B and 3A, Table 2). This large genotype effect may be due to entrainment of the circadian network in peripheral organs. The main pacemaker, the suprachiasmatic nucleus (SCN) of the hypothalamus, entrains peripheral tissues either directly through humoral and neural factors or indirectly through behaviour (locomotor activity, fasting/eating)^46^. In LD conditions the daily entrainment takes place with the onset of light. It was shown that adrenal glands are in the first line of peripheral organs that respond to light signals^47^. Several layers of signal transduction and regulation exists between the SCN and the adrenal gland (the hypothalamus-pituitary-adrenal - HPA axis and neural innervation^46^,^21^).

SNVs found by exome sequencing in genes with differential circadian or diurnal expression could not fully explain the observed expression differences. Functional characterization of genes with highest variability between the 129S2 and C57BL/6J mouse lines, followed by enrichment analysis of genes involved in adrenal and liver circadian entrainment, returned genes from Receptor, Kinase and G-protein coupled receptors functional categories. These are key pathways of circadian photoreception in the SCN and light signal transduction to the periphery^48^ (Fig. 6B). In addition to the genes that have evident role in circadian events, such as *Opn4, Vipr2* and *Npas2* described in detail in the exome sequencing results section, other genes with nonsynonymous SNVs are: neural transmitters transporters (*Slc6a4*), receptors (*Chrnb4, Nmur2*), signal proteins (*Ssfa2*), proteins involved in cholesterol to steroid hormone synthesis (*Tspo, Stard13*) and cholesterol to bile acid conversion enzyme (*Cyp7a1*). 26–31% of nonsynonymous SNPs are expected to have effect on protein function^49^, thus it is possible that some of these SNVs could influence entrainment pathways leading from SCN to the adrenal gland and result in subtle changes in circadian gene expression. One of the mouse lines used in our experiments was C57BL/6JOlaHsd. It is generally known that a related subline C57BL/N carries a mutation in *Crb1* gene known as “rd8” that causes retinal lesions. Zurita *et al*.^50^ performed SNP analysis of ten different C57BL/6 strains and found no “rd8” mutation in C57BL/6JOlaHsd subline. Therefore this mutation cannot contribute to different light perception.

Adrenal gland and liver are both governed by the SCN clock and are innervated by the splanchnic nerve, but both organs differ greatly regarding the signals for entrainment. It was shown before that the dominant factor for re-entrainment differs importantly among organs and tissues. For the liver the restricted feeding overruns HPA-axis derived glucocorticoid signals^51^. Adrenal gland is from this point of view more strongly affected by the SCN mediated light perception. Under LD conditions organism is re-entrained daily with the onset of light, so it is expected that differences in adrenal gland LD expression will be more profound than differences in the liver. Ishida *et al*.^47^ clearly showed that the re-entrainment induced by light affected adrenal gland *Per1* expression followed by a corticosterone peak, but did not affect the liver *Per1* expression. In our experimental set-up both mouse lines were housed and fed under the same conditions so the difference in the liver daily re-entrainment (in LD conditions) are not expected unless some great difference in processing of the metabolic re-entrainment signals is present due to the genotype.

The mouse lines 129S2 and C57BL/6 differ in several phenotype facts. For example, the 129 line has a lower body weight, higher daily food intake and lower water intake compared to C57BL/6^32^. Additionally, restricted feeding, glucocorticoid levels, or the temperature cycles, can all influence the circadian entrainment of peripheral organs. These phenotypic differences, even if mechanistically not well defined, could also affect metabolic regulation of the hepatic circadian rhythmicity. The insulin signalling is among better described mechanisms of hepatic clock synchronization. We found several SNVs in insulin - related genes but the effectiveness of these SNVs awaits determination. Berglund *et al*.^52^ indeed showed that 129 mice have the greatest insulin secretion and C57Bl/6J respond the fastest to hyperinsulinemia after 5h fasting. Among 4 mouse strains used in their study C57BL/6J has the highest blood glucose and glucagon levels while 129 the lowest blood glucose and insulin and highest insulin clearance.

It is not negligible that 129/S2 and C57BL/6J strains differ in the average length of free-running period (23.93 ± 0.07 h and 23.77 ± 0.02 h, respectively)^10^. They differ as well in locomotor activity with C57BL/6J mice being more active than the 129 line^55^. The behavioural activity can change the circadian clock by suppression of SCN neuronal activity^56^,^57^, so such mechanism can represent a direct influence of phenotypic differences in the circadian clock. The body temperature rhythm is lower in C57BL/6 compared to three other mouse lines^58^. Buhr *et al*.^59^ suggested that temperature is a universal resetting cue. During the restricted feeding regime, the body temperature cycles are slightly changed and this can activate the Heat-shock-protein pathway^60^. Heat shock factor 1 (HSF1) activity is strongly circadian and phosphorylated form can bind the HSE elements in Per2 promoter. HSF1 off-sync activation can cause *Per2* expression in an immediate early fashion which can re-set the clock in peripheral organs^61^,^62^,^63^. We checked if some SNVs are present in the genes important for the activation of HSP pathway: in Hsp90aa1, Hsp90ab1 and Hsf1 no SNVs were found. We checked also the two HSE regions of the *Per2* promoter^63^ and also found no SNVs between the C57Bl/6 and 129/S2 lines.

A large contributor to the genotype effects could also lie in SNPs in the core clock protein binding sites. Mutations in binding sites determined by ChIP-Seq can largely reduce the circadian amplitude of gene expression^53^. However, since the density of SNPs is enriched at the ChIP-Seq peaks of clock protein binding regions (Fig. 5B) it is expected that these SNPs indeed have an important role in regulating the circadian expression. Changes at the genomic level alone will not be able to explain all differences observed. As we have shown in our previous work, the circadian amplitude of *Cyp17a1* in adrenal glands is affected by methylation of its promoter region^54^.

In conclusion, our data show the effect of mouse genetic background on circadian expression of core clock and metabolic genes. The genotype influenced the tissue (liver and adrenal glands) and light dependent circadian (DD) or diurnal (LD) expression. While we uncovered potential SNVs that could explain these differences between the C57BL/6 and 129S2 mouse lines, confirmation of their causative effect is difficult due to complexity of the circadian system^46^. It is important to comprehend that the C57BL/6 and 129S2 mouse lines vary in tissue circadian expression patterns where the phases in C57BL/6 are generally delayed and the amplitudes lower. The experiments together with sequencing and data mining proposed the differences of clock-related transcription factors binding regions and the entrainment mechanisms of the peripheral organs as major causes for the observed circadian or diurnal differences.

## Methods

### Animal experiments, Circadian Collection of Samples and Isolation of materials

C57BL/6JOlaHsd and 129S2/SvPasCrlf × C57BL/6JRj male mice with free access to food and water were housed under a 12:12 h LD or under DD and sacrificed every 4h over a 24h period. Liver and adrenal glands were collected. RNA and DNA were isolated by standard protocols. Animal experiments were carried out in accordance with approved guidelines: European Convention for the protection of vertebrate animals used for experimental and other scientific purposes (ETS 123); National Institutes of Health guidelines for work with laboratory animals. All experimental protocols were approved by the Veterinary Administration of the Republic of Slovenia (license number 34401-9/2008/4, 34401-38/2009/2, 34401-44/2009/2).

### Gene Expression Data Analysis

RT-q PCR was performed by gene-specific primers applying the SYBR Green method and data normalized as described earlier^64^. Normalized gene expression values were analysed by the Cosinor analysis^65^, calculated and visualized in program language R^66^.

### Whole Exome Sequencing and Data Analysis

Whole exome sequencing was performed on the Illumina Hiseq2000 platform. The Burrows-Wheeler Aligner was used to align the mouse strains sequences to the NCBI reference sequence (NCBI37/mm9). The final BAM files were used as input for SOAPsnp in order to identify single nucleotide variations (Supplementary Data S1 and Supplementary Data S2). In order to predict which of the variants could have the most deleterious effect on the protein activity the Grantham Matrix Score (GMS) was determined for each nonsynonymous SNV (Fig. 4B)^67^ and DAVID applied to annotate genes belonging to the radical group. Other procedures were performed in R and Bioconductor. Whole exome sequencing data is available at the European Nucleotide Archive under the Study code: PRJEB11861 (http://www.ebi.ac.uk/ena/data/view/PRJEB11861).

### *In silico* analysis

Genomic location of genes whose circadian or diurnal expression was measured by RT-qPCR was extracted from ChIP-seq data obtained from Koike *et al*.^35^. After obtaining genomic locations of binding sites the presence of SNPs was determined with the aid of the Imputed Mouse SNP Resource^36^ (Supplementary Table S10). The NCBI Build 37 (mm9) was used as a reference for genomic locations for both databases.

Imputed Mouse SNP Resource^36^ was used to count the number of SNPs in DHS sites from Ling *et al*.^37^ supplemental Table S5A containing a merged list of Standard DHS sites. SNP density was obtained by dividing the number of SNPs by DHS site length. DHS sites of clock related protein were identified using Koike *et al*.^35^ Supplemental Table S2 containing master peak list for each transcription factor. Average SNP density for DHS regions was calculated for each clock related protein individually (i.e. BMAL1, CLOCK, NPAS2, PER1, PER2, CRY1, CRY2) and compared to an average SNP density over all standard DHS sites^37^ using a single-sample *t* test (Supplementary Table S11).

## Additional Information

**How to cite this article**: Košir, R. *et al*. Mouse genotypes drive the liver and adrenal gland clocks. *Sci. Rep.* **6**, 31955; doi: 10.1038/srep31955 (2016).

## Supplementary Material

Supplementary Table S1

Supplementary Table S2

Supplementary Information

## Figures and Tables

**Figure 1:**
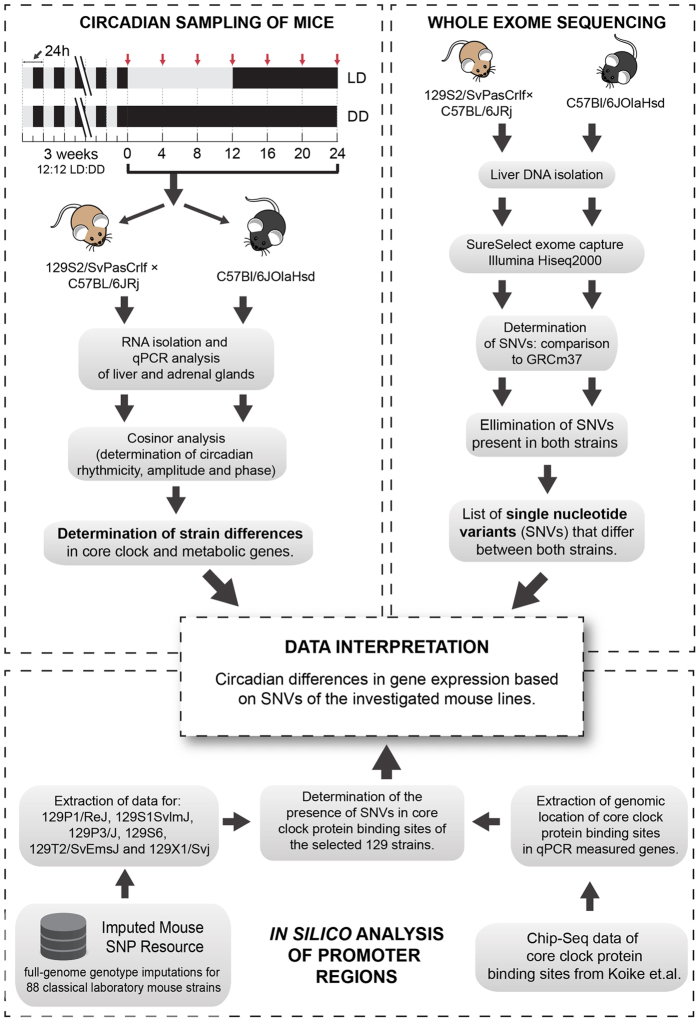
Experimental workflow. Different parts of experimental methods used to evaluate the influence of mouse genotype on circadian (DD) or diurnal (LD) expression of genes in peripheral tissues.

**Table 1:** Assessment of circadian rhythmicity in gene expression.

	129S2 strain				C57BL/6J			
	Liver		Adrenal gland		Liver		Adrenal gland	
	DD	LD	DD	LD	DD	LD	DD	LD
Core clock genes	10 (100%)	10 (100%)	10 (100%)	10 (100%)	8 (80%)	10 (100%)	9 (90%)	10 (100%)
Metabolic genes	8 (80%)	8 (80%)	1 (17%)	6 (100%)	5 (50%)	6 (60%)	3 (50%)	4 (67%)
All genes	18 (90%)	18 (90%)	11 (69%)	16 (100%)	13 (65%)	16 (80%)	12 (75%)	14 (88%)
By tissue & strain	36 (90%)		27 (84%)		29 (73%)		26 (81%)	
By strain	63 (87%)				55 (76%)			

The presence of circadian (DD) or diurnal (LD) oscillation for each gene was determined with the cosinor analysis of expression data gathered with qRT-PCR. A p-value of 0.01 was determined as a cut of value above which genes were not considered to have a circadian or diurnal expression pattern. The number and percentage of genes displaying a circadian or diurnal pattern is shown below for each of the 8 possible conditions based on the number of strains (2), tissues (2) and light conditions (2). A list of core clock and metabolic genes can be found in Supplementary Table S1.

**Table 2:** Comparison of amplitudes and phases (peak expression) of core clock and metabolic genes in 129S2 line and C57BL/6J.

	DD		LD	
	Liver	Adrenal gland	Liver	Adrenal gland
Amplitude	8 (*40%*)	0	8 (*40%*)	13 (*81%*)
	*Bmal1, Per1, Cry1, Cry2, Por, Ppara, Dbp, Rev-ErbA*		*Bmal1, Per1, Per2, Per3, Dec1, Cry2, Dbp, Rev-ErbA*	*Bmal1, Per1, Per2, Per3, Cry2, Dec1, Dec2, Dbp, Rev-ErbA, Cyp51, Cyp11a1, Cyp17a1, Cyp21a1*
Phase	4 (*20%*)	3 (*19%*)	2 (*10%*)	7 (*44%*)
	*Bmal1, Dec1, Cry1, Cyp8b1*	*Cyp51, Rev-ErbA, Cyp21a1*	*Pgc1a, Car*	*Cyp51, Cyp17a1, Cyp39a1, Per2, Dec1, Rev-ErbA, Cry1*

The amplitudes and phases were compared based on the cosinor analysis (a 95% confidence interval for amplitudes and phases was calculated for each gene). Genes that did not show a circadian or diurnal expression were not included in the analysis. A p-value of 0.01 was determined as a cut of value above which genes were not considered to be differentially expressed between stains. The number and percentage (%) of genes with different circadian or diurnal patterns for each condition and tissue are reported.

**Table 3:** Location and functional consequences of SNVs from whole exome sequencing of 129S2 line and C57BL/6J.

SNV location/type	Compared to mouse NCBI reference strain (NCBI37; mm9)					
	C57BL/6J		129S2 strain		Difference between strains(*after removal of identical SNVs*)	
Total number of SNVs	5770	(100%)	85224	(100%)	83715	(100%)
Exonic SNVs	1358	(23.54%)	20015	(23.49%)	19392	(23.16%)
* Synonymous SNVs*	*481*	(8.34%)	*12884*	(15.12%)	*12638*	(15.10%)
* Nonsynonymous SNVs*	*863*	(14.96%)	*7290*	(8.55%)	*6915*	(8.26%)
* Stopgain SNVs*	*28*	(0.49%)	*62*	(0.07%)	*50*	(0.06%)
* Stoploss SNVs*	*7*	(0.12%)	*31*	(0.04%)	*30*	(0.04%)
Both exonic region and splicing junction SNVs	21	(0.36%)	252	(0.30%)	241	(0.29%)
Splicing region SNVs	16	(0.28%)	72	(0.08%)	68	(0.08%)
5’ UTR or 3’ UTR SNVs	282	(4.89%)	3975	(4.66%)	3957	(4.73%)
Intron region SNVs	2356	(40.83%)	48842	(57.31%)	48548	(57.99%)
Intergenic SNVs	1295	(22.44%)	9241	(10.84%)	8787	(10.50%)
ncRNA	239	(4.14%)	1114	(1.31%)	1057	(1.26%)

Sequence alignment and annotation is described in Material and Methods. The exome sequences of both mouse lines were aligned to NCBI37 reference strain. The same SNV can be part of different categories, for example it can have different effects in different transcripts from the same gene.

**Table 4:** SNVs in genes with genotype related expression differences.

Symbol	Entrez ID	Number of Mutations					
		Intergenic	Intronic	3’UTR	Exonic		SUM
					Synonymous	Nonsynonimous	
*Bmal1*	11865	2	10		1		13
*Per2*	18627		9		3	1	13
*Per1*	18626		6		3		9
*Cyp21a1*	13079	1					1
*Cyp17a1*	13074		1				1
*Cyp51*	13121		1	1			2
*Dbp*	13170		1	1	1		3
*Ppargc1a*	19017	3	12		1	1	17
*Car*	12355		13		2		15
*Ppara*	19013		3				3
	**SUM**	**6**	**56**	**2**	**11**	**2**	**77**
	**%**	**8%**	**73%**	**3%**	**14%**	**3%**	

The table shows the number of SNVs that were discovered between the C57BL/6J and 129S2 strain by exome sequencing in genes whose circadian or diurnal expression profiles were affected by genotype.

## References

[b1] Dibner C. & Schibler U. Circadian timing of metabolism in animal models and humans. J Intern Med 277, 513–527 (2015). 25599827 10.1111/joim.12347

[b2] Saini C., Brown S. A. & Dibner C. Human peripheral clocks: applications for studying circadian phenotypes in physiology and pathophysiology. Front Neurol 6, 95 (2015). 26029154 10.3389/fneur.2015.00095 PMC4429585

[b3] Albrecht U. Timing to perfection: the biology of central and peripheral circadian clocks. Neuron 74, 246–260 (2012). 22542179 10.1016/j.neuron.2012.04.006

[b4] Bollinger T. & Schibler U. Circadian rhythms - from genes to physiology and disease. Swiss Med Wkly 144, w13984 (2014). 25058693 10.4414/smw.2014.13984

[b5] Zhang R., Lahens N. F., Ballance H. I., Hughes M. E. & Hogenesch J. B. A circadian gene expression atlas in mammals: Implications for biology and medicine. Proc. Natl. Acad. Sci. 111, 16219–16224 (2014). 25349387 10.1073/pnas.1408886111 PMC4234565

[b6] Arble D. M., Ramsey K. M., Bass J. & Turek F. W. Circadian disruption and metabolic disease: findings from animal models. Best Pr. Res Clin Endocrinol Metab 24, 785–800 (2010). 10.1016/j.beem.2010.08.003 PMC3011935 21112026

[b7] Troublesome variability in mouse studies. Nat Neurosci 12, 1075 (2009). 19710643 10.1038/nn0909-1075

[b8] Turek F. W. . Obesity and metabolic syndrome in circadian Clock mutant mice. Science 308, 1043–1045 (2005). 15845877 10.1126/science.1108750 PMC3764501

[b9] Kennaway D. J., Owens J. A., Voultsios A., Boden M. J. & Varcoe T. J. Metabolic homeostasis in mice with disrupted Clock gene expression in peripheral tissues. Am J Physiol Regul Integr Comp Physiol 293, R1528–R1537 (2007). 17686888 10.1152/ajpregu.00018.2007

[b10] Schwartz W. J. & Zimmerman P. Circadian timekeeping in BALB/c and C57BL/6 inbred mouse strains. J. Neurosci. 10, 3685–3694 (1990). 2230953 10.1523/JNEUROSCI.10-11-03685.1990 PMC6570095

[b11] Ebihara S., Tsuji K. & Kondo K. Strain differences of the mouse’s free-running circadian rhythm in continuous darkness. Physiol Behav 20, 795–799 (1978). 684115 10.1016/0031-9384(78)90308-6

[b12] Abe H., Kida M., Tsuji K. & Mano T. Feeding cycles entrain circadian rhythms of locomotor activity in CS mice but not in C57BL/6J mice. Physiol Behav 45, 397–401 (1989). 2756028 10.1016/0031-9384(89)90146-7

[b13] Ebihara S., Goto M. & Oshima I. The phase-shifting effects of pentobarbital on the circadian rhythm of locomotor activity in the mouse: strain differences. Brain Res 454, 404–407 (1988). 3409025 10.1016/0006-8993(88)90847-5

[b14] Kasahara T., Abe K., Mekada K., Yoshiki A. & Kato T. Genetic variation of melatonin productivity in laboratory mice under domestication. Proc Natl Acad Sci U A 107, 6412–6417 (2010). 10.1073/pnas.0914399107 PMC2851971 20308563

[b15] Adams D. J., Doran A. G., Lilue J. & Keane T. M. The Mouse Genomes Project: a repository of inbred laboratory mouse strain genomes. Mamm Genome, 10.1007/s00335-015-9579-6 (2015). 26123534

[b16] van der Horst G. T. . Mammalian Cry1 and Cry2 are essential for maintenance of circadian rhythms. Nature 398, 627–630 (1999). 10217146 10.1038/19323

[b17] Bae K. . Differential functions of mPer1, mPer2, and mPer3 in the SCN circadian clock. Neuron 30, 525–536 (2001). 11395012 10.1016/s0896-6273(01)00302-6

[b18] Cermakian N., Monaco L., Pando M. P., Dierich A. & Sassone-Corsi P. Altered behavioral rhythms and clock gene expression in mice with a targeted mutation in the Period1 gene. EMBO J 20, 3967–3974 (2001). 11483500 10.1093/emboj/20.15.3967 PMC149149

[b19] Muller U. Ten years of gene targeting: targeted mouse mutants, from vector design to phenotype analysis. Mech Dev 82, 3–21 (1999). 10354467 10.1016/s0925-4773(99)00021-0

[b20] Korencic A. . Timing of circadian genes in mammalian tissues. Sci Rep 4, 5782 (2014). 25048020 10.1038/srep05782 PMC5376044

[b21] Mohawk J. A., Green C. B. & Takahashi J. S. Central and peripheral circadian clocks in mammals. Annu Rev Neurosci 35, 445–462 (2012). 22483041 10.1146/annurev-neuro-060909-153128 PMC3710582

[b22] Sheward W. J. . Entrainment to feeding but not to light: circadian phenotype of VPAC2 receptor-null mice. J Neurosci 27, 4351–4358 (2007). 17442819 10.1523/JNEUROSCI.4843-06.2007 PMC6672325

[b23] DeBruyne J. P., Weaver D. R. & Reppert S. M. CLOCK and NPAS2 have overlapping roles in the suprachiasmatic circadian clock. Nat Neurosci 10, 543–545 (2007). 17417633 10.1038/nn1884 PMC2782643

[b24] Rice D. A., Mouw A. R., Bogerd A. M. & Parker K. L. A shared promoter element regulates the expression of three steroidogenic enzymes. Mol Endocrinol 5, 1552–1561 (1991). 1775136 10.1210/mend-5-10-1552

[b25] Blasic J. R. Jr., Lane Brown R. & Robinson P. R. Light-dependent phosphorylation of the carboxy tail of mouse melanopsin. Cell Mol Life Sci 69, 1551–1562 (2012). 22159583 10.1007/s00018-011-0891-3 PMC4045631

[b26] Sexton T. J. & Van Gelder R. N. G-Protein Coupled Receptor Kinase 2 Minimally Regulates Melanopsin Activity in Intrinsically Photosensitive Retinal Ganglion Cells. PLos One 10, e0128690 (2015). 26069965 10.1371/journal.pone.0128690 PMC4467020

[b27] Roecklein K. A. . A missense variant (P10L) of the melanopsin (OPN4) gene in seasonal affective disorder. J Affect Disord 114, 279–285 (2009). 18804284 10.1016/j.jad.2008.08.005 PMC2647333

[b28] Harmar A. J. . The VPAC2 Receptor Is Essential for Circadian Function in the Mouse Suprachiasmatic Nuclei. Cell 109, 497–508 (2002). 12086606 10.1016/s0092-8674(02)00736-5

[b29] Dudley C. A. . Altered Patterns of Sleep and Behavioral Adaptability in NPAS2-Deficient Mice. Science 301, 379–383 (2003). 12843397 10.1126/science.1082795

[b30] Hammer G. D. . Phosphorylation of the Nuclear Receptor SF-1 Modulates Cofactor Recruitment: Integration of Hormone Signaling in Reproduction and Stress. Mol. Cell 3, 521–526 (1999). 10230405 10.1016/s1097-2765(00)80480-3

[b31] Harris A. N. & Mellon P. L. The Basic Helix-Loop-Helix, Leucine Zipper Transcription Factor, USF (Upstream Stimulatory Factor), Is a Key Regulator of SF-1 (Steroidogenic Factor-1) Gene Expression in Pituitary Gonadotrope and Steroidogenic Cells. Mol. Endocrinol. 12, 714–726 (1998). 9605934 10.1210/mend.12.5.0100

[b32] Bachmanov A. A., Reed D. R., Beauchamp G. K. & Tordoff M. G. Food Intake, Water Intake, and Drinking Spout Side Preference of 28 Mouse Strains. Behav. Genet. 32, 435–443 (2002). 12467341 10.1023/a:1020884312053 PMC1397713

[b33] Sato M., Murakami M., Node K., Matsumura R. & Akashi M. The Role of the Endocrine System in Feeding-Induced Tissue-Specific Circadian Entrainment. Cell Rep. 8, 393–401 (2014). 25017062 10.1016/j.celrep.2014.06.015

[b34] Taniguchi C. M., Emanuelli B. & Kahn C. R. Critical nodes in signalling pathways: insights into insulin action. Nat. Rev. Mol. Cell Biol. 7, 85–96 (2006). 16493415 10.1038/nrm1837

[b35] Koike N. . Transcriptional Architecture and Chromatin Landscape of the Core Circadian Clock in Mammals. Science 338, 349–354 (2012). 22936566 10.1126/science.1226339 PMC3694775

[b36] Wang J. R. . Imputation of single-nucleotide polymorphisms in inbred mice using local phylogeny. Genetics 190, 449–458 (2012). 22345612 10.1534/genetics.111.132381 PMC3276610

[b37] Ling G., Sugathan A., Mazor T., Fraenkel E. & Waxman D. J. Unbiased, Genome-Wide *In Vivo* Mapping of Transcriptional Regulatory Elements Reveals Sex Differences in Chromatin Structure Associated with Sex-Specific Liver Gene Expression. Mol. Cell. Biol. 30, 5531–5544 (2010). 20876297 10.1128/MCB.00601-10 PMC2976433

[b38] Bryda E. C. The Mighty Mouse: the impact of rodents on advances in biomedical research. Mo Med 110, 207–211 (2013). 23829104 PMC3987984

[b39] Lowrey P. L. & Takahashi J. S. Genetics of circadian rhythms in Mammalian model organisms. Adv Genet 74, 175–230 (2011). 21924978 10.1016/B978-0-12-387690-4.00006-4 PMC3709251

[b40] Rossner M. J. . Disturbed clockwork resetting in Sharp-1 and Sharp-2 single and double mutant mice. PLos One 3, e2762 (2008). 18648504 10.1371/journal.pone.0002762 PMC2447179

[b41] Bass J. & Takahashi J. S. Circadian integration of metabolism and energetics. Science 330, 1349–1354 (2010). 21127246 10.1126/science.1195027 PMC3756146

[b42] Liu C., Li S., Liu T., Borjigin J. & Lin J. D. Transcriptional coactivator PGC-1α integrates the mammalian clock and energy metabolism. Nature 447, 477–481 (2007). 17476214 10.1038/nature05767

[b43] Charoensuksai P. & Xu W. PPARs in Rhythmic Metabolic Regulation and Implications in Health and Disease. PPAR Res 2010, (2010). 10.1155/2010/243643 PMC2943104 20871864

[b44] Contreras A. V., Torres N. & Tovar A. R. PPAR-α as a Key Nutritional and Environmental Sensor for Metabolic Adaptation. Adv. Nutr. Int. Rev. J. 4, 439–452 (2013). 10.3945/an.113.003798 PMC3941823 23858092

[b45] Cantó C. & Auwerx J. PGC-1α, SIRT1 and AMPK, an energy sensing network that controls energy expenditure: Curr. Opin. Lipidol. 20, 98–105 (2009). 19276888 10.1097/MOL.0b013e328328d0a4 PMC3627054

[b46] Dibner C., Schibler U. & Albrecht U. The mammalian circadian timing system: organization and coordination of central and peripheral clocks. Annu Rev Physiol 72, 517–549 (2010). 20148687 10.1146/annurev-physiol-021909-135821

[b47] Ishida A. . Light activates the adrenal gland: timing of gene expression and glucocorticoid release. Cell Metab. 2, 297–307 (2005). 16271530 10.1016/j.cmet.2005.09.009

[b48] Zhu H. . Integrative Gene Regulatory Network Analysis Reveals Light-Induced Regional Gene Expression Phase Shift Programs in the Mouse Suprachiasmatic Nucleus. PLos ONE 7, e37833 (2012). 22662235 10.1371/journal.pone.0037833 PMC3360606

[b49] Chasman D. & Adams R. M. Predicting the functional consequences of non-synonymous single nucleotide polymorphisms: structure-based assessment of amino acid variation. J. Mol. Biol. 307, 683–706 (2001). 11254390 10.1006/jmbi.2001.4510

[b50] Zurita E. . Genetic polymorphisms among C57BL/6 mouse inbred strains. Transgenic Res. 20, 481–489 (2010). 20506040 10.1007/s11248-010-9403-8

[b51] Sujino M. . Differential entrainment of peripheral clocks in the rat by glucocorticoid and feeding. Endocrinology 153, 2277–2286 (2012). 22434077 10.1210/en.2011-1794

[b52] Berglund E. D. . Glucose metabolism *in vivo* in four commonly used inbred mouse strains. Diabetes 57, 1790–1799 (2008). 18398139 10.2337/db07-1615 PMC2453626

[b53] Hatanaka F. . Genome-wide profiling of the core clock protein BMAL1 targets reveals a strict relationship with metabolism. Mol Cell Biol 30, 5636–5648 (2010). 20937769 10.1128/MCB.00781-10 PMC3004277

[b54] Kosir R. . Circadian expression of steroidogenic cytochromes P450 in the mouse adrenal gland–involvement of cAMP-responsive element modulator in epigenetic regulation of Cyp17a1. FEBS J 279, 1584–1593 (2012). 21883931 10.1111/j.1742-4658.2011.08317.x

[b55] Lightfoot J. T. . Strain screen and haplotype association mapping of wheel running in inbred mouse strains. J. Appl. Physiol. 109, 623–634 (2010). 20538847 10.1152/japplphysiol.00525.2010 PMC2944645

[b56] Yamazaki S., Kerbeshian M. C., Hocker C. G., Block G. D. & Menaker M. Rhythmic Properties of the Hamster Suprachiasmatic NucleusIn Vivo. J. Neurosci. 18, 10709–10723 (1998). 9852606 10.1523/JNEUROSCI.18-24-10709.1998 PMC6793356

[b57] van Oosterhout F. . Amplitude of the SCN Clock Enhanced by the Behavioral Activity Rhythm. PLos ONE 7, e39693 (2012). 22761873 10.1371/journal.pone.0039693 PMC3386260

[b58] Connolly M. S. & Lynch C. B. Circadian variation of strain differences in body temperature and activity in mice. Physiol. Behav. 27, 1045–1049 (1981). 7335804 10.1016/0031-9384(81)90368-1

[b59] Buhr E. D., Yoo S.-H. & Takahashi J. S. Temperature as a Universal Resetting Cue for Mammalian Circadian Oscillators. Science 330, 379–385 (2010). 20947768 10.1126/science.1195262 PMC3625727

[b60] Reinke H. . Differential display of DNA-binding proteins reveals heat-shock factor 1 as a circadian transcription factor. Genes Dev. 22, 331–345 (2008). 18245447 10.1101/gad.453808 PMC2216693

[b61] Kornmann B., Schaad O., Bujard H., Takahashi J. S. & Schibler U. System-Driven and Oscillator-Dependent Circadian Transcription in Mice with a Conditionally Active Liver Clock. PLos Biol 5, e34 (2007). 17298173 10.1371/journal.pbio.0050034 PMC1783671

[b62] Chappuis S. . Role of the circadian clock gene Per2 in adaptation to cold temperature. Mol. Metab. 2, 184–193 (2013). 24049733 10.1016/j.molmet.2013.05.002 PMC3773826

[b63] Tamaru T. . Synchronization of Circadian Per2 Rhythms and HSF1-BMAL1:CLOCK Interaction in Mouse Fibroblasts after Short-Term Heat Shock Pulse. PLos ONE 6, e24521 (2011). 21915348 10.1371/journal.pone.0024521 PMC3168500

[b64] Kosir R. . Determination of reference genes for circadian studies in different tissues and mouse strains. BMC Mol Biol 11, 60 (2010). 20712867 10.1186/1471-2199-11-60 PMC2928770

[b65] Nelson W., Tong Y. L., Lee J. K. & Halberg F. Methods for cosinor-rhythmometry. Chronobiologia 6, 305–323 (1979). 548245

[b66] Wickham H. Ggplot2: elegant graphics for data analysis. (Springer, 2009).

[b67] Rudd M. F. . The predicted impact of coding single nucleotide polymorphisms database. Cancer Epidemiol Biomark. Prev 14, 2598–2604 (2005). 10.1158/1055-9965.EPI-05-0469 16284384

