# Supplementary material for: Mouse genotypes drive the liver and adrenal gland clocks
Source: Sci Rep. 2016 Aug 18;6:31955. doi: 10.1038/srep31955 (PMC4989183; doi:10.1038/srep31955)
Supplement: Supplementary Information [file srep31955-s3.doc]

## Supplementary information for manuscript:

# Mouse genotypes drive the liver and adrenal gland clocks

Rok Košir^1+^, Uršula Prosenc Zmrzljak^1+^, Anja Korenčič ^1^, Peter Juvan ^1^, Jure Ačimovič ^2^ and Damjana Rozman^1,2, *^

## Supplementary Methods

## Supplementary Figures

**Supplementary Figure S1:** Gene expression analysis of core clock and metabolic genes in liver samples

**Supplementary Figure S2:** Gene expression analysis of core clock and metabolic genes in adrenal gland samples

## Supplementary Tables

**Supplementary Table S1:** Information for primers used in qRT-PCR measurements

**Supplementary Table S2:** Determination of circadian expression of genes under LD and DD conditions in liver and adrenal glands of both strains

**Supplementary Table S3:** Differences in amplitude and phase found between 129Pas and C57BL6/OlaHsd strains

**Supplementary Table S4:** Whole Exome Sequencing raw and cleaned data statistics

**Supplementary Table S5:** Alignment statistics

**Supplementary Table S6:** Ensembl Variant Effect Predictor (VEP) results for the 42 SNV discovered by exome sequencing

**Supplementary Table S7:** List of genes involved in circadian entrainment of adrenal glands based on scientific publications.

**Supplementary Table S8:** Single nucleotide variants (SNVs) discovered by whole exome sequencing

**Supplementary Table S9:** A list of SNVs present in the insulin signaling pathway genes.

**Supplementary Table S10:** A list of SNPs present in binding regions of core clock proteins.

**Supplementary Table S11**: Analysis of SNPs from binding regions of clock core genes in open chromatin regions.

## Supplementary References

## Supplementary Methods

### Animal experiments

65 C57BL/6JOlaHsd and 69 strain 129S2/SvPasCrlf×C57BL/6JRj male mice were maintained in a temperature and humidity controlled room under a 12:12 h light:dark cycle (light on at 7:00 am, light off at 7:00 pm) with free access to food (Harlan Teklad 2916) and acidified water (pH = 3). The experiment was approved by the Veterinary Administration of the Republic of Slovenia (license number 34401-9/2008/4, 34401-38/2009/2, 34401-44/2009/2) and was conducted in accordance with the European Convention for the protection of vertebrate animals used for experimental and other scientific purposes (ETS 123) as well as in accordance with National Institutes of Health guidelines for work with laboratory animals. In the continuation of the article the full names of strain are abbreviated: C57BL/6JOlaHsd as C57BL/6J and 129S2/SvPasCrlf×C57BL/6JRj as 129S2. For the 129S2 mixed strain the genomic background was composed of 75% 129S2/SvPas and 25% C57BL/6J based on the original strain received from Nantel *et al* ^1^.

### Circadian Collection of Samples and RNA Isolation

Mice were sacrificed every 4h over a 24h period with cervical dislocation under light-dark (LD) and dark-dark (DD) conditions. Mice sacrificed under DD conditions were kept in complete darkness for 36 h prior to scarification. Liver and adrenal glands were excised, snap frozen in liquid nitrogen and stored at -80°C. Total RNA from homogenized liver and adrenal gland samples was isolated according to the manufactures instructions using 1000 μl and 500 μl of TRI reagent (Sigma) respectively. Quality and quantity of RNA was determined using NanoDrop 1100.

Genomic DNA from both strains was isolated from liver using the Phenol:Chloroform:Isoamyl Alcohol (Sigma) and according to the manufactures instructions. Prior to extraction 20-30 mg of pulverized liver tissue was homogenised in tissue lysis buffer. 5 μl of (10mg/ml) proteinase-K and 10 μl of RNaseA (10mg/ml) were added and the mixture was incubated over night at 56°C on a heating shaking drybath at 500 rpm. Quality and quantity of isolated DNA was determined using gel electrophoresis and NanoDrop 1000 respectively.

### Primer Design and RT-qPCR

Wherever possible, intron spanning primers were designed based on publicly available sequences (Supplementary TableS1). Primer specificity and amplification efficiency were validated empirically using a melting curve and standard curve analysis of a six fold dilution series.

DNAse treatment was performed on all samples using DNAse I (Roche Applied Bioscience) according to the manufacturer's instructions. cDNA synthesis was carried out using SuperScript III reverse transcriptase (Invitrogen). 3 μg of liver RNA was mixed together with 20 μl of reverse transcriptase master mix which contained 8 μl of 5 × first strand buffer, 2 μl of 100 mM DTT, 2 μl of 10 mM dNTP mix, 1 μl of random primers (Promega 500 ng/μl), 0.75 μl of SuperScript III (200 U/μl), 0.75 μl of RNAse OUT (Invitrogen) and 5.5 μl of RNAse free water in a final volume of 40 μl. 1 μg of adrenal gland RNA was mixed together with 10 μl of reverse transcriptase master mix which contained 5 μl of 5x first strand buffer, 1.25 μl of 100 mM DTT, 1.25 μl of 10 mM dNTP mix, 0.65 μl of random primers (Promega 500 ng/μl), 0.5 μl of SuperScript III (200 U/μl), 0.5 μl of RNAse OUT (Invitrogen) and 0.85 μl of RNAse free water in a final volume of 25 μl. The reaction mixtures were incubated at 25°C for 5 minutes, 50°C for 60 minutes and 70°C for 10 minutes.

Real time quantitative PCR was performed in a 384 well format on LightCycler 480 (Roche Applied Science) using LightCycler 480 SYBR Green I Master (Roche Applied Science). The PCR reaction consisted of 2.5 μl of SYBR Green I Master, 1.15 μl of RNAse free water, 0.6 μl of 300 nM primer mix and 0.75 μl of cDNA in a total volume of 5 μl. Three technical replicates were performed for each sample. Cycling conditions were as follows: 10 min at 95°C followed by 40 rounds of 10 s at 95°C, 20 s at 60°C and 20 s at 72 °C. Melting curve analysis for determining the dissociation of PCR products was performed from 65°C to 95°C.

The Cp values of expressed genes were transformed into quantities by taking into account primer efficiencies. These quantities were then normalised by a normalisation factor, i.e. the geometric mean of the expression of the reference genes for liver (*Utp6c*, *Hprt1*) and adrenal gland (*Rn18s*, *Ppib*, *Rplp0*) as determined in a prior study^2^.

### Gene Expression Data Analysis

Normalized gene expression values were analysed using the Cosinor analysis to determine the presence of circadian rhythmicity. Cosinor analysis is based on the least squares approximation of time series data with a cosine function of known period (24h in our case)^3^. It provides information about mesor (mean of expression), amplitude (difference between peak and trough of expression), acrophase (time of peak expression) and a statistical value for a null hypothesis of zero amplitude. p values of 0.01 or smaller were considered to represent circadian (DD) or diurnal (LD) expression. The Cosinor analysis was performed in the R statistical programming language (version 3.1.2) with the use of package “cosinor” (version 1.1). All graphical presentations were done in the R package ggplot2^4^.

### Whole Exome Sequencing

Whole exome sequencing of both strains was performed on liver extracted DNA with the help of Agilent SureSelect exome capture protocol on Illumina Hiseq2000 platform system according to the manufacturer's instructions. In brief, genomic DNA was randomly fragmented by Covaris to a size between 150 to 200bp. Adapters were ligated to both ends of the resulting fragments and purified using Agencourt AMPure SPRI beads and fragments with insert size about 176bp were excised. Extracted DNA was amplified by ligation-mediated PCR (LM-PCR), purified, and hybridized to the SureSelect Biotiny lated RNA Library (BAITS) for enrichment. Captured LM-PCR products were subjected to Agilent 2100 Bioanalyzer. Each captured library was loaded on Hiseq2000 platform for high-throughput sequencing. Raw image files were processed by Illumina basecalling Software 1.7 for base-calling with default parameters and the sequences of each individual were generated as 90/100bp pair-end reads.

### Whole Exome Data Analysis

The raw data generated from the Illumina pipeline was firstly cleaned of adapter sequences; low quality reads (reads where half of bases have a read quality of < 5) and reads with more than 10% of unknown bases (Supplementary Table S4). The Burrows-Wheeler Aligner (BWA) was used to align the strains sequences to the NCBI reference sequence (NCBI37/mm9). The final BAM files (produced by the BWA) were used as input for SOAPsnp in order to identify single nucleotide variation present in sequenced strains. After SNV identification AnnoDB was used to annotate and classify found variation. Single nucleotide variation results can be downloaded from Supplementary data as csv files (Supplementary Data S1 and Supplementary Data S2). In order to predict which of the variants could have the most deleterious effect on the protein activity the Grantham Matrix Score (GMS) was determined for each nonsynonymous SNV (Figure 4B). The GMS is based on changes in chemical properties of amino acids that are substituted. Depending on the GMS, SNVs were classified into conservative (GMS 0-50), moderately conservative (GMS 51-100), moderately radical (GMS 101-150) and radical (GMS > 151) group^5^. Database for Annotation Visualization and Integrated Discovery DAVID was used to annotate genes belonging to the radical group. All additional procedures from the csv files onward were conducted using the R statistical programming language (version 3.1.2), Bioconductor (3.0) and the R package ggplot2 for graphical presentation.

### *In silico* analysis of promoter regions of genes of interest

Genomic location of core clock protein binding sites for BMAL1, PER1, PER2, CRY1, CRY2, CLOCK and NPAS2 in genes whose circadian or diurnal expression was measured by RT-qPCR was extracted from ChiP-seq data obtained from Koike et al^6^. These data contained information of the start and end location of the binding region, chromosome number, peak location, peak height and corresponding gene symbol. After obtaining genomic locations of binding sites we determined the presence of single nucleotide polymorphisms in these regions. In order to do this we obtained data of genomic locations of SNPs present in six 129 strains from the Imputed Mouse SNP Resource^7^, which stores information on SNPs for 88 inbred mouse strains. Data were downloaded for each chromosome and each strain separately. We determined the presence of imputed SNPs in the binding sites of core clock proteins (Supplementary Table S8). The NCBI Build 37 (mm9) was used as a reference for genomic locations for both databases. The determination of SNP density in binding sites of tissue important transcription factor s in Per1 promoter region was carried out as follows: sequence was obtained from NCBI, transcription start site was determined according to RefSeq NM_001159367 and 20kb upstream sequence was analysed. The prediction of binding sites of tissue important transcription factors was performed with the Tansfac tool ^8^. SNP density was compared between six 129 strains and C57BL/6J. All statistical analyses were performed with the statistical language R.

## Supplementary Figures

**Supplementary Figure S1:** Gene expression analysis of core clock and metabolic genes in liver samples. X axis shows the circadian time (CT); Y axis shows relative gene expression.
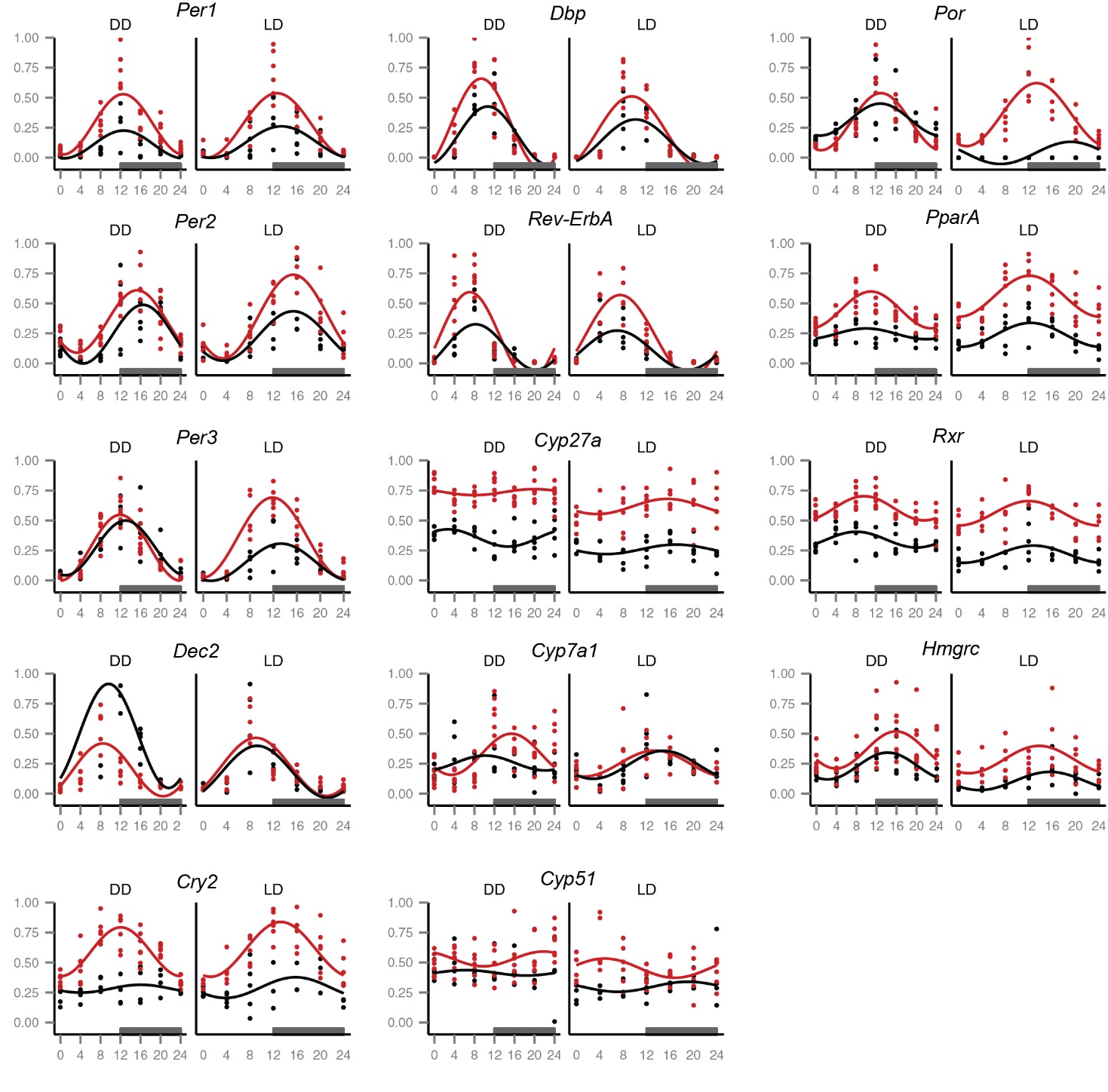


**Supplementary Figure S2:** Gene expression analysis of core clock and metabolic genes in adrenal gland samples. X axis shows the circadian time (CT); Y axis shows relative gene expression.

#
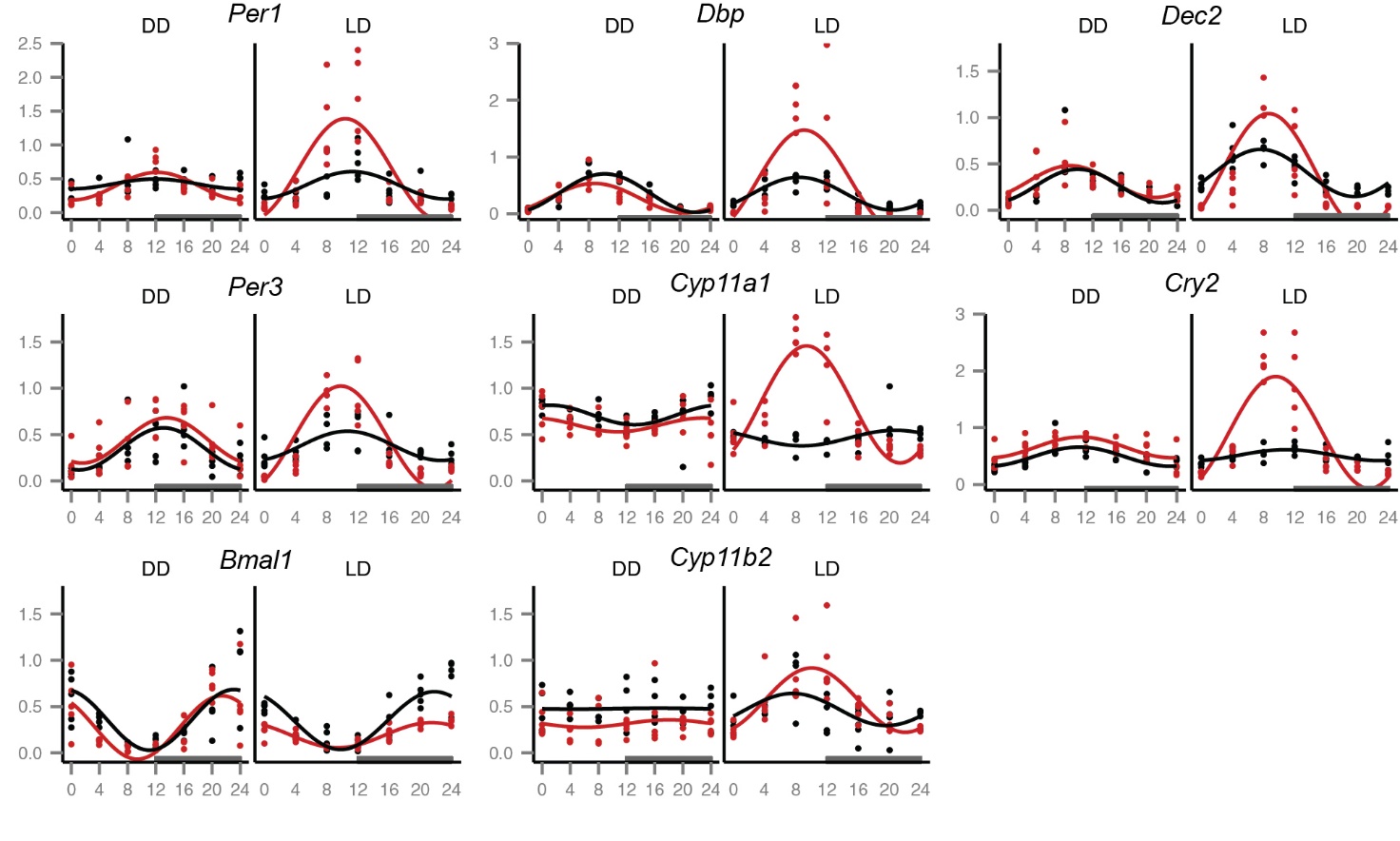


# Supplementary Tables

**Supplementary Table S1: Information for primers used in qRT-**PCR measurements.

| **Gene** | **NCBI ID** |  | **Sequence** | **Lenght of primer in [bp]** | **Amplification**  **factor** |
| --- | --- | --- | --- | --- | --- |
| ***Reference genes*** | |  |  |  |  |
| *Rplp0* | NM_007475.4 | fw | *CACTGGTCTAGGACCCGAGAAG* | 22 | 1,98 |
|  |  | rv | *GGTGCCTCTGGAGATTTTCG* | 20 |  |
| *Ppib* | NM_011149.2 | fw | *GGAGATGGCACAGGAGGAAA* | 20 | 1,93 |
|  |  | rv | *CCGTAGTGCTTCAGTTTGAAGTTCT* | 25 |  |
| *Hprt1* | NM_013556.2 | fw | *TCCTCCTCAGACCGCTTTT* | 19 | 1,89 |
|  |  | rv | *CCTGGTTCATCATCGCTAATC* | 21 |  |
| *Rn18s* | NR_003278.1 | fw | *CGCCGCTAGAGGTGAAATTC* | 20 | 1,79 |
|  |  | rv | *TTGGCAAATGCTTTCGCTC* | 19 |  |
| *Utp6* | NM_144826.3 | fw | *TTTCGGTTGAGTTTTTCAGGA* | 21 | 1,82 |
|  |  | rv | *CCCTCAGGTTTACCATCTTGC* | 21 |  |
| ***Circadian genes – core clock*** | |  |  |  |  |
| *Arntl – Bmal1* | NM_007489 | fw | *GCAGTGCCACTGACTACCAAGA* | 22 | 1.94 |
|  |  | rv | *TCCTGGACATTGCATTGCAT* | 20 |  |
| *Dbp* | NM_016974.2 | fw | *AATGACCTTTGAACCTGATCCCGCT* | 25 | 1.93 |
|  |  | rv | *GCTCCAGTACTTCTCATCCTTCTGT* | 25 |  |
| *Per1* | NM_011065.3 | fw | *TCCTCCTCCTACACTGCCTCT* | 21 | 1.90 |
|  |  | rv | *TTGCTGACGACGGATCTTT* | 19 |  |
| *Per2* | NM_011066.3 | fw | *CAACACAGACGACAGCATCA* | 20 | 1.94 |
|  |  | rv | *TCCTGGTCCTCCTTCAACAC* | 20 |  |
| *Per3* | NM_011067.1 | fw | *CTGCTCCAACTCAGCTTCCTTT* | 22 | 2.00 |
|  |  | rv | *TTAGACAGCAAGGCTCTGGTTCT* | 23 |  |
| *Cry1* | NM_007771 | fw | *CCCAGGCTTTTCAAGGAATGGAACA* | 25 | 1.98 |
|  |  | rv | *TCTCATCATGGTCATCAGACAGAGG* | 25 |  |
| *Cry2* | NM_009963 | fw | *AGGGCTGCCAAGTGCATCAT* | 20 | 2.00 |
|  |  | rv | *AGGAAGGGACAGATGCCAATAG* | 22 |  |
| *Bhlhe40 – Dec1* | NM_011498.4 | fw | *TCTCCTACCCGAACATCTCAA* | 21 | 1.95 |
|  |  | rv | *AATGCTTTCACGTGCTTCAA* | 20 |  |
| *Bhlhe41 – Dec2* | NM_024469.1 | fw | *ATTGCTTTACAGAATGGGGAGCG* | 23 | 1.89 |
|  |  | rv | *AAAGCGCGCGAGGTATTGCAAGAC* | 24 |  |
| *Nr1d1 - RevErba* | NM_145434.3 | fw | *ACGACCCTGGACTCCAATAA* | 20 | 2.00 |
|  |  | rv | *CCATTGGAGCTGTCACTGTAGA* | 22 |  |
| ***Metabolic genes*** | |  |  |  |  |
| *Rxra* | NM_011305.3 | fw | *GCTTCGGGACTGGTAGCC* | 18 | 1,99 |
|  |  | rv | *GCGGCTTGATATCCTCAGTG* | 20 |  |
| *Cyp51a1* | NM_020010.2 | fw | *ACGCTGCCTGGCTATTGC* | 18 | 1,86 |
|  |  | rv | *TTGATCTCTCGATGGGCTCTATC* | 23 |  |
| *Cyp11a1* | NM_019779.3 | fw | *AAGTATGGCCCCATTTACAGG* | 21 | 1,99 |
|  |  | rv | *TGGGGTCCACGATGTAAACT* | 20 |  |
| *Cyp39a1* | NM_018887.3 | fw | *ACCTATGATGAGGGCTTTGAGTA* | 23 | 1,84 |
|  |  | rv | *CCATCTTTTGGATTTTGACCA* | 21 |  |
| *Hmgcr* | NM_008255.2 | fw | *CTTGTGGAATGCCTTGTGATTG* | *22* | 1.90 |
|  |  | rv | *AGCCGAAGCAGCACATGAT* | *19* |  |
| *Por* | NM_008898.1 | fw | *GGGAACTTGGAAGAGGATTTCA* | *22* | 2.00 |
|  |  | rv | *CCCGAAGAACTCGCACACA* | *19* |  |
| *Ppara* | NM_011144.6 | fw | *CCTCTTCCCAAAGCTCCTTCA* | *21* | 2.00 |
|  |  | rv | *CGTCGGACTCGGTCTTCTTG* | *20* |  |
| *Cyp7a1* | NM_007824.2 | fw | *TCTCAAGCAAACACCATTCCT* | *21* | 1.94 |
|  |  | rv | *GGCTGCTTTCATTGCTTCA* | *19* |  |
| *Cyp8b1* | NM_010012.2 | fw | *GGAAGTGAGCCAGTTGCAG* | *19* | 1.70 |
|  |  | rv | *TTTTCCAGGTTTTGCTCCAC* | *20* |  |
| *Cyp11b2* | NM_009991.3 | fw | *GCACCAGGTGGAGAGTATGC* | *20* | 1.78 |
|  |  | rv | *GCCATTCTGGCCCATTTAG* | *19* |  |
| *Cyp17a1* | NM_007809.3 | fw | *CATCCCACACAAGGCTAACA* | *20* | 1.97 |
|  |  | rv | *CAGTGCCCAGAGATTGATGA* | *20* |  |
| *Cyp21a1* | NM_009995.2 | fw | *AGGAATTCTCCTTCCTCACTTGT* | *23* | 1.99 |
|  |  | rv | *TCTGTACCAACGTGCTGTCC* | *20* |  |
| *Nr1i3 - Car* | NM_009803.4 | fw | *CAGGGTTCCAGTACGAGTTTTG* | 22 | 2,00 |
|  |  | rv | *AGGCTCCTGGAGATGCAGTC* | 20 |  |
| *Ppargc1a* | NM_008904.2 | fw | *CATTTGATGCACTGACAGATGGA* | 23 | 1,94 |
|  |  | rv | *CCGTCAGGCATGGAGGAA* | 18 |  |
| *Cyp27a1* | NM_024264.4 | fw | *CCTCACCTATGGGATCTTCATC* | 22 | 1,87 |
|  |  | rv | *TTTAAGGCATCCGTGTAGAGC* | 21 |  |

**Supplementary Table S2: Determination of circadian expression of genes under LD and DD conditions in liver and adrenal glands of both strains.** Circadian rhythmicity was determined with cosinor analysis (period=24h). A p value of 0.01 was selected as a cutoff and genes with p values above this were defined as not circadian. YES: gene is circadian; NO: gene is not circadian. Red boxes indicate genes where under the same conditions the circadian oscillation is present in one but not the other genotype.

|  |  | **Liver** | | | | **Adrenal gland** | | | |
| --- | --- | --- | --- | --- | --- | --- | --- | --- | --- |
|  |  | **DD** | | **LD** | | **DD** | | **LD** | |
|  | **Gene** | 129Pas | C57BL6 | 129Pas | C57BL6 | 129Pas | C57BL6 | 129Pas | C57BL6 |
| 1 | *Arntl* | YES | YES | YES | YES | YES | YES | YES | YES |
| 2 | *Cry1* | YES | YES | YES | YES | YES | YES | YES | YES |
| 3 | *Cry2* | YES | NO | YES | YES | YES | YES | YES | YES |
| 4 | *Dbp* | YES | YES | YES | YES | YES | YES | YES | YES |
| 5 | *Bhlhe40* | YES | YES | YES | YES | YES | YES | YES | YES |
| 6 | *Bhlhe41* | YES | NO | YES | YES | YES | YES | YES | YES |
| 7 | *Per1* | YES | YES | YES | YES | YES | NO | YES | YES |
| 8 | *Per2* | YES | YES | YES | YES | YES | YES | YES | YES |
| 9 | *Per3* | YES | YES | YES | YES | YES | YES | YES | YES |
| 10 | *Nr1d1* | YES | YES | YES | YES | YES | YES | YES | YES |
| 11 | *Nr1i3* | YES | NO | YES | YES |  |  |  |  |
| 12 | *Cyp11a1* |  |  |  |  | NO | YES | YES | YES |
| 13 | *Cyp11b2* |  |  |  |  | NO | NO | YES | YES |
| 14 | *Cyp17a1* |  |  |  |  | NO | NO | YES | YES |
| 15 | *Cyp21a1* |  |  |  |  | NO | YES | YES | NO |
| 16 | *Cyp27a1* | NO | YES | NO | NO |  |  |  |  |
| 17 | *Cyp39a1* |  |  |  |  | NO | NO | YES | NO |
| 18 | *Cyp51* | NO | NO | NO | NO | YES | YES | YES | YES |
| 19 | *Cyp7a1* | YES | NO | YES | YES |  |  |  |  |
| 20 | *Cyp8b1* | YES | YES | YES | NO |  |  |  |  |
| 21 | *Hmgcr* | YES | YES | YES | YES |  |  |  |  |
| 22 | *Pgc1a* | YES | NO | *YES* | YES |  |  |  |  |
| 23 | *Por* | YES | YES | YES | NO |  |  |  |  |
| 24 | *PparA* | YES | NO | YES | YES |  |  |  |  |
| 25 | *Rxr* | YES | YES | YES | YES |  |  |  |  |

**Supplementary Table S3: Differences in amplitude and phase found between 129Pas and C57BL6/OlaHsd strains.** Yes indicates a statistically significant difference with a p value of less than 0.01. Cosinor analysis was applied for analysis of circadian data as described in methods and materials.

| **Liver** |  |  |  |  |
| --- | --- | --- | --- | --- |
|  | **DD** | | **LD** | |
| **Gene** | **Amplitude** | **Phase** | **Amplitude** | **Phase** |
| *Arntl* | YES | YES | YES | - |
| *Per3* | - | - | YES | - |
| *Per2* | - | - | YES | - |
| *Per1* | YES | - | YES | - |
| *Bhlhe40* | - | YES | YES | - |
| *Bhlhe41* | - | - | - | - |
| *Cry1* | YES | YES | - | - |
| *Cry2* | YES | - | YES | - |
| *Dbp* | YES | - | YES | - |
| *Nr1d1* | YES | - | YES | - |
| *Por* | YES | - | - | - |
| *PparA* | YES | - | - | - |
| *Rxr* | - | - | - | - |
| *Hmgcr* | - | - | - | - |
| *Pgc1a* | - | - | - | YES |
| *Cyp8b1* | - | YES | - | - |
| *Cyp27a1* | - | - | - | - |
| *Cyp7a1* | - | - | - | - |
| *Nr1i3* | - | - | - | YES |
| *Cyp51* | - | - | - | - |
| % of circadian genes | | | | |
| Circadian | 60% | 30% | 80% | 0% |
| Metabolic | 20% | 10% | 0% | 20% |
| All | 40% | 20% | 40% | 10% |
|  |  |  |  |  |
| **Adrenal gland** |  |  |  |  |
|  | **DD** | | **LD** | |
| **Gene** | **Amplitude** | **Phase** | **Amplitude** | **Phase** |
| *Arntl* | - | - | YES | - |
| *Per3* | - | - | YES | - |
| *Per2* | - | - | YES | YES |
| *Per1* | - | - | YES | - |
| *Bhlhe40* | - | - | YES | YES |
| *Bhlhe41* | - | - | YES | - |
| *Cry1* | - | - | - | YES |
| *Cry2* | - | - | YES | - |
| *Dbp* | - | - | YES | - |
| *Nr1d1* | - | YES | YES | YES |
| *Cyp51a1* | - | YES | YES | YES |
| *Cyp11a1* | - | - | YES | - |
| *Cyp11b2* | - | - | - | - |
| *Cyp17a1* | - | - | YES | YES |
| *Cyp21a1* | - | YES | - | - |
| *Cyp39a1* | - | - | YES | YES |
| % of circadian genes | | | | |
| Circadian | 0% | 10% | 90% | 40% |
| Metabolic | 0% | 20% | 40% | 30% |
| All | 0% | 19% | 81% | 44% |

**Supplementary Table S4: Whole Exome Sequencing raw and cleaned data statistics.**

| **Strain: C57BL6/OlaHsd** |  | |
| --- | --- | --- |
| **Type** | **Raw data** | **Clean data** |
| Number of Reads | 90,455,078 | 85,956,146 |
| Data Size | 9,045,507,800 | 8,595,614,600 |
| N of fq1 | 670,931 | 224,926 |
| N of fq2 | 8,636,041 | 713,446 |
| GC(%) of fq1 | 47.84 % | 47.73 % |
| GC(%) of fq2 | 47.89 % | 47.82 % |
| Q20(%) of fq1 | 96.34 % | 97.28 % |
| Q20(%) of fq2 | 93.16 % | 96.22 % |
| Q30(%) of fq1 | 90.00 % | 91.20 % |
| Q30(%) of fq2 | 86.37 % | 89.41 % |
| **Discard Reads from raw data due to:** |  | |
| Unknown bases | 240,200 | |
| Low quality | 3,812,596 | |
| Adapter | 446,136 | |
|  |  | |
| Clean data/Raw data | 0.95 % | |

| **Strain: 129S2/SvPasCrlf × C57BL/6JRj** | | |
| --- | --- | --- |
| **Type** | **Raw data** | **Clean data** |
| Number of Reads | 88,363,954 | 84,084,924 |
| Data Size | 8,836,395,400 | 8,408,492,400 |
| N of fq1 | 631,309 | 218,313 |
| N of fq2 | 8,247,681 | 691,093 |
| GC(%) of fq1 | 47.64 % | 47.53 % |
| GC(%) of fq2 | 47.74 % | 47.67 % |
| Q20(%) of fq1 | 96.18 % | 97.16 % |
| Q20(%) of fq2 | 93.23 % | 96.11 % |
| Q30(%) of fq1 | 89.67 % | 90.92 % |
| Q30(%) of fq2 | 86.29 % | 89.16 % |
| **Discard Reads from raw data due to:** |  | |
| Unknown bases | 229,054 | |
| Low quality | 3,604,848 | |
| Adapter | 445,128 | |
|  |  | |
| Clean data/Raw data | 0.95 % | |

**Supplementary** Table S5: Alignment statistics

| **Sample** | **C57BL6/OlaHsd** | **129S2/SvPasCrlf × C57BL/6JRj** |
| --- | --- | --- |
| Initial bases on target | 51734315 | 51734315 |
| Initial bases near target | 71994574 | 71994574 |
| Initial bases on or near target | 123728889 | 123728889 |
| Total effective reads | 76994687 | 75457786 |
| Total effective yield( Mb) | 7327.35 | 7176.51 |
| Average read length (bp) | 95.17 | 95.11 |
| Effective sequences on target(Mb) | 4488.69 | 3965.84 |
| Effective sequences near target(Mb) | 1533.8 | 1428.54 |
| Effective sequences on or near target(Mb) | 6022.49 | 5394.38 |
| Number of reads uniquely mapped to target | 49423386 | 43785379 |
| Number of reads uniquely mapped to genome | 66772998 | 65254798 |
| Fraction of effective bases on target | 61.30 % | 55.30% |
| Fraction of uniquely mapped on target | 74.00 % | 67.10% |
| Fraction of effective bases on or near target | 82.20 % | 75.20% |
| Average sequencing depth on target | 86.76 | 76.66 |
| Average sequencing depth near target | 21.3 | 19.84 |
| Mismatch rate in target region | 0.18 % | 0.26 % |
| Mismatch rate in all effective sequence | 0.18 % | 0.27 % |
| Base covered on target | 51680016 | 51656335 |
| Coverage of target region | 99.90 % | 99.80 % |
| Base covered near target | 69115196 | 69508914 |
| Coverage of flanking region | 96.00 % | 96.50 % |
| Fraction of target covered with at least 20x | 92.00 % | 89.40 % |
| Fraction of target covered with at least 10x | 97.90 % | 97.10 % |
| Fraction of target covered with at least 4x | 99.50 % | 99.30 % |
| Fraction of flanking region covered with at least 20x | 31.60 % | 29.80 % |
| Fraction of flanking region covered with at least 10x | 52.80 % | 52.20 % |
| Fraction of flanking region covered with at least 4x | 78.20 % | 79.40 % |
| Mapping rate | 98.62 % | 97.99 % |
| Duplicate rate | 9.17 % | 8.42 % |

**Supplementary** Table S6: Ensembl Variant Effect Predictor (VEP) results for the 42 SNV discovered by exome sequencing.

| **Symbol** | **Uploaded variation** | **Allele** | **Gene** | **Feature** | **Feature type** | **Consequence** | **cDNA position** | **CDS position** | **Protein position** | **Amino acids** | **Codons** | **Existing variation** | **Feature strand** | **Symbol source** | **Biotype** |
| --- | --- | --- | --- | --- | --- | --- | --- | --- | --- | --- | --- | --- | --- | --- | --- |
| Olfr421-ps1 | 1_174152324_A/G | G | ENSMUSG00000091950 | ENSMUST00000179386 | Transcript | stop_lost | 844 | 807 | 269 | */W | TGA/TGG | rs32233283 | 1 | MGI | polymorphic_pseudogene |
| Bpnt1 | 1_185352356_C/T | T | ENSMUSG00000026617 | ENSMUST00000027916 | Transcript | stop_gained | 771 | 649 | 217 | R/* | CGA/TGA | - | 1 | MGI | protein_coding |
| Ctdsp1 | 1_74394966_C/T | T | ENSMUSG00000026176 | ENSMUST00000027367 | Transcript | stop_gained | 990 | 601 | 201 | R/* | CGA/TGA | - | 1 | MGI | protein_coding |
| Znhit3 | 11_84911469_A/C | C | ENSMUSG00000020526 | ENSMUST00000103195 | Transcript | stop_lost | 479 | 454 | 152 | */E | TAA/GAA | - | -1 | MGI | protein_coding |
| Serpina3i | 12_104266573_G/T | T | ENSMUSG00000079014 | ENSMUST00000109958 | Transcript | stop_gained | 975 | 736 | 246 | E/* | GAG/TAG | rs29190168 | 1 | MGI | protein_coding |
| Gm5800 | 14_51711958_C/A | A | ENSMUSG00000068506 | ENSMUST00000095916 | Transcript | stop_gained | 588 | 529 | 177 | E/* | GAG/TAG | rs50996648 | -1 | MGI | protein_coding |
| Dok2 | 14_70778068_A/T | T | ENSMUSG00000022102 | ENSMUST00000022698 | Transcript | stop_gained | 1368 | 1234 | 412 | K/* | AAG/TAG | rs30442595 | 1 | MGI | protein_coding |
| Gfra2 | 14_70978491_C/T | T | ENSMUSG00000022103 | ENSMUST00000022699 | Transcript | stop_gained | 2115 | 1390 | 464 | Q/* | CAG/TAG | rs217095385 | 1 | MGI | protein_coding |
| Sub1 | 15_11991015_T/A | A | ENSMUSG00000022205 | ENSMUST00000110504 | Transcript | stop_gained | 253 | 139 | 47 | R/* | AGA/TGA | - | -1 | MGI | protein_coding |
| 2410089E03Rik | 15_8247033_T/A | A | ENSMUSG00000039801 | ENSMUST00000110617 | Transcript | stop_gained | 8681 | 8160 | 2720 | C/* | TGT/TGA | - | 1 | MGI | protein_coding |
| Vmn2r114 | 17_23308041_C/A | A | ENSMUSG00000091945 | ENSMUST00000168033 | Transcript | stop_gained | 1516 | 1516 | 506 | G/* | GGA/TGA | - | -1 | MGI | protein_coding |
| Vmn2r115 | 17_23359627_C/A | A | ENSMUSG00000091076 | ENSMUST00000168175 | Transcript | stop_gained | 2073 | 2073 | 691 | Y/* | TAC/TAA | - | 1 | MGI | protein_coding |
| Vmn2r117 | 17_23478356_G/A | A | ENSMUSG00000091407 | ENSMUST00000171996 | Transcript | stop_gained | 361 | 361 | 121 | Q/* | CAA/TAA | - | -1 | MGI | protein_coding |
| March5 | 19_37221485_T/A | A | ENSMUSG00000023307 | ENSMUST00000128530 | Transcript | stop_gained | 541 | 543 | 181 | Y/* | TAT/TAA | - | 1 | MGI | protein_coding |
| Hoga1 | 19_42070335_T/A | A | ENSMUSG00000025176 | ENSMUST00000081714 | Transcript | stop_lost | 1050 | 964 | 322 | */K | TAA/AAA | rs30403166 | 1 | MGI | protein_coding |
| A430105I19Rik | 2_118762625_T/A | A | ENSMUSG00000045838 | ENSMUST00000059997 | Transcript | stop_gained | 37 | 28 | 10 | R/* | AGA/TGA | rs32920032 | -1 | MGI | protein_coding |
| Sycp1 | 3_102841035_G/A | A | ENSMUSG00000027855 | ENSMUST00000029448 | Transcript | stop_gained | 2569 | 2440 | 814 | Q/* | CAG/TAG | - | -1 | MGI | protein_coding |
| Ankrd35 | 3_96683592_T/G | G | ENSMUSG00000038354 | ENSMUST00000048427 | Transcript | stop_gained | 1473 | 1193 | 398 | L/* | TTA/TGA | rs108544930 | 1 | MGI | protein_coding |
| Ermap | 4_119188896_C/T | T | ENSMUSG00000028644 | ENSMUST00000141227 | Transcript | stop_gained,   NMD_transcript_variant | 239 | 36 | 12 | W/* | TGG/TGA | rs27526098 | -1 | MGI | nonsense_mediated_decay |
| Iqcc | 4_129616471_C/T | T | ENSMUSG00000040795 | ENSMUST00000046675 | Transcript | stop_gained | 1294 | 1250 | 417 | W/* | TGG/TAG | rs27503438 | -1 | MGI | protein_coding |
| Nudt9 | 5_104050699_C/T | T | ENSMUSG00000029310 | ENSMUST00000150226 | Transcript | stop_gained | 435 | 79 | 27 | R/* | CGA/TGA | - | 1 | MGI | protein_coding |
| Hscb | 5_110829121_T/A | A | ENSMUSG00000043510 | ENSMUST00000056937 | Transcript | stop_lost | 753 | 704 | 235 | */L | TAG/TTG | rs216609005 | -1 | MGI | protein_coding |
| Mmp17 | 5_129606538_A/G | G | ENSMUSG00000029436 | ENSMUST00000031390 | Transcript | stop_lost | 1861 | 1737 | 579 | */W | TGA/TGG | rs29636438 | 1 | MGI | protein_coding |
| Cyp3a44 | 5_145803723_G/A | A | ENSMUSG00000054417 | ENSMUST00000067479 | Transcript | stop_gained | 191 | 106 | 36 | Q/* | CAG/TAG | - | -1 | MGI | protein_coding |
| Mug2 | 6_122085838_T/C | C | ENSMUSG00000030131 | ENSMUST00000081777 | Transcript | stop_lost | 4415 | 4354 | 1452 | */R | TGA/CGA | rs30311216 | 1 | MGI | protein_coding |
| Olfr67 | 7_103787315_A/G | G | ENSMUSG00000047535 | ENSMUST00000183254 | Transcript | stop_lost | 961 | 961 | 321 | */Q | TAA/CAA | rs46474932 | -1 | MGI | protein_coding |
| Trim12c | 7_104340787_C/T | T | ENSMUSG00000057143 | ENSMUST00000059037 | Transcript | stop_gained | 1707 | 1482 | 494 | W/* | TGG/TGA | rs31820711 | -1 | MGI | protein_coding |
| Vmn2r79 | 7_87037967_G/T | T | ENSMUSG00000090362 | ENSMUST00000164462 | Transcript | stop_lost | 2555 | 2555 | 852 | */L | TGA/TTA | rs32071141 | 1 | MGI | protein_coding |
| Mcf2l | 8_13018964_G/T | T | ENSMUSG00000031442 | ENSMUST00000110867 | Transcript | stop_lost | 3553 | 3305 | 1102 | */L | TGA/TTA | rs33032580 | 1 | MGI | protein_coding |
| Defb46 | 8_19242065_C/A | A | ENSMUSG00000071169 | ENSMUST00000095436 | Transcript | stop_gained | 96 | 96 | 32 | C/* | TGC/TGA | rs47398835 | 1 | MGI | protein_coding |
| Adam3 | 8_24680719_T/G | G | ENSMUSG00000031553 | ENSMUST00000033958 | Transcript | stop_lost | 2564 | 2468 | 823 | */S | TAA/TCA | rs47374095 | -1 | MGI | protein_coding |
| Cd209e | 8_3849087_C/T | T | ENSMUSG00000040197 | ENSMUST00000033888 | Transcript | stop_gained | 680 | 624 | 208 | W/* | TGG/TGA | rs32973852 | -1 | MGI | protein_coding |
| Podnl1 | 8_84132225_T/C | C | ENSMUSG00000012889 | ENSMUST00000093380 | Transcript | stop_lost | 1788 | 1678 | 560 | */Q | TAG/CAG | rs50121794 | 1 | MGI | protein_coding |
| Peak1 | 9_56260385_A/T | T | ENSMUSG00000074305 | ENSMUST00000061552 | Transcript | stop_gained | 727 | 258 | 86 | C/* | TGT/TGA | - | -1 | MGI | protein_coding |
| Mroh2a | 1_88235214_C/T | T | ENSMUSG00000079429 | ENSMUST00000113130 | Transcript | stop_gained | 1281 | 1069 | 357 | Q/* | CAG/TAG | rs48492920 | 1 | MGI | protein_coding |
| Mroh2a | 1_88237586_C/A | A | ENSMUSG00000079429 | ENSMUST00000113130 | Transcript | stop_gained | 1640 | 1428 | 476 | Y/* | TAC/TAA | rs49147968 | 1 | MGI | protein_coding |
| Hjurp | 1_88266526_A/T | T | ENSMUSG00000044783 | ENSMUST00000065420 | Transcript | stop_gained | 519 | 431 | 144 | L/* | TTA/TAA | - | -1 | MGI | protein_coding |
| Skint7 | 4_111982261_C/T | T | ENSMUSG00000049214 | ENSMUST00000142162 | Transcript | upstream_gene_variant | - | - | - | - | - | rs27484814 | 1 | MGI | retained_intron |
| Klra4 | 6_130065245_G/A | A | ENSMUSG00000079852 | ENSMUST00000119096 | Transcript | stop_gained | 203 | 58 | 20 | Q/* | CAG/TAG | - | -1 | MGI | protein_coding |
| Klra8 | 6_130128151_G/A | A | ENSMUSG00000089727 | ENSMUST00000014476 | Transcript | stop_gained | 155 | 10 | 4 | Q/* | CAG/TAG | - | -1 | MGI | protein_coding |
| Art2a-ps | 7_101554849_A/G | G | ENSMUSG00000092517 | ENSMUST00000173420 | Transcript | stop_lost | 551 | 481 | 161 | */R | TGA/CGA | rs31290728 | -1 | MGI | polymorphic_pseudogene |
| Olfr332 | 11_58489748_C/A | A | ENSMUSG00000050813 | ENSMUST00000180165 | Transcript | stop_gained | 1572 | 1006 | 336 | E/* | GAA/TAA | rs29484442 | -1 | MGI | protein_coding |

**Supplementary Table S7**

List of genes involved in circadian entrainment of adrenal glands based on scientific publications.

| **EntrezID** | **Symbol** | **Chr** | **Ensenbl** | **Gene Name** |
| --- | --- | --- | --- | --- |
| 18143 | *Npas2* | 1 | ENSMUSG00000026077 | neuronal PAS domain protein 2 |
| 18627 | *Per2* | 1 | ENSMUSG00000055866 | period homolog 2 (Drosophila) |
| 12227 | *Btg2* | 1 | ENSMUSG00000020423 | B cell translocation gene 2, anti-proliferative |
| 12912 | *Creb1* | 1 | ENSMUSG00000025958 | cAMP responsive element binding protein 1 |
| 12790 | *Cnga3* | 1 | ENSMUSG00000026114 | cyclic nucleotide gated channel alpha 3 |
| 18514 | *Pbx1* | 1 | ENSMUSG00000052534 | pre B cell leukemia homeobox 1 |
| 26424 | *Nr5a2* | 1 | ENSMUSG00000026398 | nuclear receptor subfamily 5, group A, member 2 |
| 12952 | *Cry1* | 10 | ENSMUSG00000020038 | cryptochrome 1 (photolyase-like) |
| 22353 | *Vip* | 10 | ENSMUSG00000019772 | vasoactive intestinal polypeptide |
| 13654 | *Egr2* | 10 | ENSMUSG00000037868 | early growth response 2 |
| 17873 | *Gadd45b* | 10 | ENSMUSG00000015312 | growth arrest and DNA-damage-inducible 45 beta |
| 22288 | *Utrn* | 10 | ENSMUSG00000019820 | utrophin |
| 54140 | *Avpr1a* | 10 | ENSMUSG00000020123 | arginine vasopressin receptor 1A |
| 14816 | *Grm1* | 10 | ENSMUSG00000019828 | glutamate receptor, metabotropic 1 |
| 18101 | *Nmbr* | 10 | ENSMUSG00000019865 | neuromedin B receptor |
| 216343 | *Tph2* | 10 | ENSMUSG00000006764 | tryptophan hydroxylase 2 |
| 76142 | *Ppp1r14c* | 10 | ENSMUSG00000040653 | protein phosphatase 1, regulatory (inhibitor) subunit 14c |
| 18626 | *Per1* | 11 | ENSMUSG00000020893 | period homolog 1 (Drosophila) |
| 217166 | *Nr1d1* | 11 | ENSMUSG00000020889 | nuclear receptor subfamily 1, group D, member 1 |
| 104318 | *Csnk1d* | 11 | ENSMUSG00000025162 | casein kinase 1, delta |
| 216850 | *Kdm6b* | 11 | ENSMUSG00000018476 | KDM1 lysine (K)-specific demethylase 6B |
| 14394 | *Gabra1* | 11 | ENSMUSG00000010803 | gamma-aminobutyric acid (GABA) A receptor, subunit alpha 1 |
| 14799 | *Gria1* | 11 | ENSMUSG00000020524 | glutamate receptor, ionotropic, AMPA1 (alpha 1) |
| 14813 | *Grin2c* | 11 | ENSMUSG00000020734 | glutamate receptor, ionotropic, NMDA2C (epsilon 3) |
| 12323 | *Camk2b* | 11 | ENSMUSG00000057897 | calcium/calmodulin-dependent protein kinase II, beta |
| 19052 | *Ppp2ca* | 11 | ENSMUSG00000020349 | protein phosphatase 2 (formerly 2A), catalytic subunit, alpha isoform |
| 18750 | *Prkca* | 11 | ENSMUSG00000050965 | protein kinase C, alpha |
| 19416 | *Rasd1* | 11 | ENSMUSG00000049892 | RAS, dexamethasone-induced 1 |
| 18588 | *Pde6g* | 11 | ENSMUSG00000025386 | phosphodiesterase 6G, cGMP-specific, rod, gamma |
| 12921 | *Crhr1* | 11 | ENSMUSG00000018634 | corticotropin releasing hormone receptor 1 |
| 13195 | *Ddc* | 11 | ENSMUSG00000020182 | dopa decarboxylase |
| 11298 | *Aanat* | 11 | ENSMUSG00000020804 | arylalkylamine N-acetyltransferase |
| 11548 | *Adra1b* | 11 | ENSMUSG00000050541 | adrenergic receptor, alpha 1b |
| 15567 | *Slc6a4* | 11 | ENSMUSG00000020838 | solute carrier family 6 (neurotransmitter transporter, serotonin), member 4 |
| 14674 | *Gna13* | 11 | ENSMUSG00000020611 | guanine nucleotide binding protein, alpha 13 |
| 19084 | *Prkar1a* | 11 | ENSMUSG00000020612 | protein kinase, cAMP dependent regulatory, type I, alpha |
| 27376 | *Slc25a10* | 11 | ENSMUSG00000025792 | solute carrier family 25 (mitochondrial carrier, dicarboxylate transporter), member 10 |
| 18948 | *Pnmt* | 11 | ENSMUSG00000038216 | phenylethanolamine-N-methyltransferase |
| 14281 | *Fos* | 12 | ENSMUSG00000021250 | FBJ osteosarcoma oncogene |
| 22355 | *Vipr2* | 12 | ENSMUSG00000011171 | vasoactive intestinal peptide receptor 2 |
| 15519 | *Hsp90aa1* | 12 | ENSMUSG00000021270 | heat shock protein 90, alpha (cytosolic), class A member 1 |
| 18976 | *Pomc* | 12 | ENSMUSG00000020660 | pro-opiomelanocortin-alpha |
| 15902 | *Id2* | 12 | ENSMUSG00000020644 | inhibitor of DNA binding 2 |
| 19088 | *Prkar2b* | 12 | ENSMUSG00000002997 | protein kinase, cAMP dependent regulatory, type II beta |
| 20620 | *Plk2* | 13 | ENSMUSG00000021701 | polo-like kinase 2 |
| 20499 | *Slc12a7* | 13 | ENSMUSG00000017756 | solute carrier family 12, member 7 |
| 26401 | *Map3k1* | 13 | ENSMUSG00000021754 | mitogen-activated protein kinase kinase kinase 1 |
| 218397 | *Rasa1* | 13 | ENSMUSG00000021549 | RAS p21 protein activator 1 |
| 15550 | *Htr1a* | 13 | ENSMUSG00000021721 | 5-hydroxytryptamine (serotonin) receptor 1A |
| 353187 | *Nr1d2* | 14 | ENSMUSG00000021775 | nuclear receptor subfamily 1, group D, member 2 |
| 30044 | *Opn4* | 14 | ENSMUSG00000021799 | opsin 4 (melanopsin) |
| 14702 | *Gng2* | 14 | ENSMUSG00000043004 | guanine nucleotide binding protein (G protein), gamma 2 |
| 12647 | *Chat* | 14 | ENSMUSG00000021919 | choline acetyltransferase |
| 27373 | *Csnk1e* | 15 | ENSMUSG00000022433 | casein kinase 1, epsilon |
| 15370 | *Nr4a1* | 15 | ENSMUSG00000023034 | nuclear receptor subfamily 4, group A, member 1 |
| 223775 | *Pim3* | 15 | ENSMUSG00000035828 | proviral integration site 3 |
| 15499 | *Hsf1* | 15 | ENSMUSG00000022556 | heat shock factor 1 |
| 110115 | *Cyp11b1* | 15 | ENSMUSG00000075604 | cytochrome P450, family 11, subfamily b, polypeptide 1 |
| 20512 | *Slc1a3* | 15 | ENSMUSG00000005360 | solute carrier family 1 (glial high affinity glutamate transporter), member 3 |
| 12257 | *Tspo* | 15 | ENSMUSG00000041736 | translocator protein |
| 53357 | *Pla2g6* | 15 | ENSMUSG00000042632 | phospholipase A2, group VI |
| 20683 | *Sp1* | 15 | ENSMUSG00000001280 | trans-acting transcription factor 1 |
| 56637 | *Gsk3b* | 16 | ENSMUSG00000022812 | glycogen synthase kinase 3 beta |
| 12914 | *Crebbp* | 16 | ENSMUSG00000022521 | CREB binding protein |
| 26413 | *Mapk1* | 16 | ENSMUSG00000063358 | mitogen-activated protein kinase 1 |
| 224129 | *Adcy5* | 16 | ENSMUSG00000022840 | adenylate cyclase 5 |
| 19252 | *Dusp1* | 17 | ENSMUSG00000024190 | dual specificity phosphatase 1 |
| 11516 | *Adcyap1* | 17 | ENSMUSG00000024256 | adenylate cyclase activating polypeptide 1 |
| 15516 | *Hsp90ab1* | 17 | ENSMUSG00000023944 | heat shock protein 90 alpha (cytosolic), class B member 1 |
| 13079 | *Cyp21a1* | 17 | ENSMUSG00000024365 | cytochrome P450, family 21, subfamily a, polypeptide 1 |
| 16440 | *Itpr3* | 17 | ENSMUSG00000042644 | inositol 1,4,5-triphosphate receptor 3 |
| 18754 | *Prkce* | 17 | ENSMUSG00000045038 | protein kinase C, epsilon |
| 225642 | *Grp* | 18 | ENSMUSG00000024517 | gastrin releasing peptide |
| 13653 | *Egr1* | 18 | ENSMUSG00000038418 | early growth response 1 |
| 106957 | *Slc39a6* | 18 | ENSMUSG00000024270 | solute carrier family 39 (metal ion transporter), member 6 |
| 12322 | *Camk2a* | 18 | ENSMUSG00000024617 | calcium/calmodulin-dependent protein kinase II alpha |
| 225600 | *Pde6a* | 18 | ENSMUSG00000024575 | phosphodiesterase 6A, cGMP-specific, rod, alpha |
| 17200 | *Mc2r* | 18 | ENSMUSG00000045569 | melanocortin 2 receptor |
| 12916 | *Crem* | 18 | ENSMUSG00000063889 | cAMP responsive element modulator |
| 14815 | *Nr3c1* | 18 | ENSMUSG00000024431 | nuclear receptor subfamily 3, group C, member 1 |
| 170459 | *Stard4* | 18 | ENSMUSG00000024378 | StAR-related lipid transfer (START) domain containing 4 |
| 225998 | *Rorb* | 19 | ENSMUSG00000036192 | RAR-related orphan receptor beta |
| 19045 | *Ppp1ca* | 19 | ENSMUSG00000040385 | protein phosphatase 1, catalytic subunit, alpha isoform |
| 19091 | *Prkg1* | 19 | ENSMUSG00000052920 | protein kinase, cGMP-dependent, type I |
| 15566 | *Htr7* | 19 | ENSMUSG00000024798 | 5-hydroxytryptamine (serotonin) receptor 7 |
| 13074 | *Cyp17a1* | 19 | ENSMUSG00000003555 | cytochrome P450, family 17, subfamily a, polypeptide 1 |
| 11554 | *Adrb1* | 19 | ENSMUSG00000035283 | adrenergic receptor, beta 1 |
| 14675 | *Gna14* | 19 | ENSMUSG00000024697 | guanine nucleotide binding protein, alpha 14 |
| 14682 | *Gnaq* | 19 | ENSMUSG00000024639 | guanine nucleotide binding protein, alpha q polypeptide |
| 12953 | *Cry2* | 2 | ENSMUSG00000068742 | cryptochrome 2 (photolyase-like) |
| 11998 | *Avp* | 2 | ENSMUSG00000037727 | arginine vasopressin |
| 18111 | *Nnat* | 2 | ENSMUSG00000067786 | neuronatin |
| 18227 | *Nr4a2* | 2 | ENSMUSG00000026826 | nuclear receptor subfamily 4, group A, member 2 |
| 70599 | *Ssfa2* | 2 | ENSMUSG00000027007 | sperm specific antigen 2 |
| 14810 | *Grin1* | 2 | ENSMUSG00000026959 | glutamate receptor, ionotropic, NMDA1 (zeta 1) |
| 57138 | *Slc12a5* | 2 | ENSMUSG00000017740 | solute carrier family 12, member 5 |
| 72425 | *slc12a6* | 2 | ENSMUSG00000027132 | RIKEN cDNA 2410042D21 gene |
| 246313 | *Prokr2* | 2 | ENSMUSG00000050558 | prokineticin receptor 2 |
| 26423 | *Nr5a1* | 2 | ENSMUSG00000026751 | nuclear receptor subfamily 5, group A, member 1 |
| 22431 | *Wt1* | 2 | ENSMUSG00000016458 | Wilms tumor 1 homolog |
| 12608 | *Cebpb* | 2 | ENSMUSG00000056501 | CCAAT/enhancer binding protein (C/EBP), beta |
| 22259 | *Nr1h3* | 2 | ENSMUSG00000002108 | nuclear receptor subfamily 1, group H, member 3 |
| 20192 | *Ryr3* | 2 | ENSMUSG00000057378 | ryanodine receptor 3 |
| 18549 | *Pcsk2* | 2 | ENSMUSG00000027419 | proprotein convertase subtilisin/kexin type 2 |
| 11438 | *Chrna4* | 2 | ENSMUSG00000027577 | cholinergic receptor, nicotinic, alpha polypeptide 4 |
| 13166 | *Dbh* | 2 | ENSMUSG00000000889 | dopamine beta hydroxylase |
| 19885 | *Rorc* | 3 | ENSMUSG00000028150 | RAR-related orphan receptor gamma |
| 56706 | *Ccnl1* | 3 | ENSMUSG00000027829 | cyclin L1 |
| 99929 | *Tiparp* | 3 | ENSMUSG00000034640 | TCDD-inducible poly(ADP-ribose) polymerase |
| 14800 | *Gria2* | 3 | ENSMUSG00000033981 | glutamate receptor, ionotropic, AMPA2 (alpha 2) |
| 18176 | *Nras* | 3 | ENSMUSG00000027852 | neuroblastoma ras oncogene |
| 18749 | *Prkacb* | 3 | ENSMUSG00000005034 | protein kinase, cAMP dependent, catalytic, beta |
| 14686 | *Gnat2* | 3 | ENSMUSG00000009108 | guanine nucleotide binding protein, alpha transducing 2 |
| 12918 | *Crh* | 3 | ENSMUSG00000049796 | corticotropin releasing hormone |
| 14696 | *Gnb4* | 3 | ENSMUSG00000027669 | guanine nucleotide binding protein (G protein), beta 4 |
| 18759 | *Prkci* | 3 | ENSMUSG00000037643 | protein kinase C, iota |
| 11444 | *Chrnb2* | 3 | ENSMUSG00000027950 | cholinergic receptor, nicotinic, beta polypeptide 2 (neuronal) |
| 18628 | *Per3* | 4 | ENSMUSG00000028957 | period homolog 3 (Drosophila) |
| 16476 | *Jun* | 4 | ENSMUSG00000052684 | Jun oncogene |
| 16600 | *Klf4* | 4 | ENSMUSG00000003032 | Kruppel-like factor 4 (gut) |
| 74178 | *Stk40* | 4 | ENSMUSG00000042608 | serine/threonine kinase 40 |
| 242425 | *Gabbr2* | 4 | ENSMUSG00000039809 | gamma-aminobutyric acid (GABA) B receptor, 2 |
| 12307 | *Calb1* | 4 | ENSMUSG00000028222 | calbindin 1 |
| 22417 | *Wnt4* | 4 | ENSMUSG00000036856 | wingless-related MMTV integration site 4 |
| 26971 | *Pla2g2f* | 4 | ENSMUSG00000028749 | phospholipase A2, group IIF |
| 12753 | *Clock* | 5 | ENSMUSG00000029238 | circadian locomotor output cycles kaput |
| 19092 | *Prkg2* | 5 | ENSMUSG00000029334 | protein kinase, cGMP-dependent, type II |
| 242851 | *Gnat3* | 5 | ENSMUSG00000028777 | guanine nucleotide binding protein, alpha transducing 3 |
| 18587 | *Pde6b* | 5 | ENSMUSG00000029491 | phosphodiesterase 6B, cGMP, rod receptor, beta polypeptide |
| 18125 | *Nos1* | 5 | ENSMUSG00000029361 | nitric oxide synthase 1, neuronal |
| 20778 | *Scarb1* | 5 | ENSMUSG00000037936 | scavenger receptor class B, member 1 |
| 18984 | *Por* | 5 | ENSMUSG00000005514 | P450 (cytochrome) oxidoreductase |
| 14677 | *Gnai1* | 5 | ENSMUSG00000057614 | guanine nucleotide binding protein (G protein), alpha inhibiting 1 |
| 19085 | *Prkar1b* | 5 | ENSMUSG00000025855 | protein kinase, cAMP dependent regulatory, type I beta |
| 243362 | *Stard13* | 5 | ENSMUSG00000016128 | StAR-related lipid transfer (START) domain containing 13 |
| 15563 | *Htr5a* | 5 | ENSMUSG00000039106 | 5-hydroxytryptamine (serotonin) receptor 5A |
| 11423 | *Ache* | 5 | ENSMUSG00000023328 | acetylcholinesterase |
| 20893 | *Bhlhe40* | 6 | ENSMUSG00000030103 | basic helix-loop-helix family, member e40 |
| 79362 | *Bhlhe41* | 6 | ENSMUSG00000030256 | basic helix-loop-helix family, member e41 |
| 212541 | *Rho* | 6 | ENSMUSG00000030324 | rhodopsin |
| 12057 | *Opn1sw* | 6 | ENSMUSG00000058831 | opsin 1 (cone pigments), short-wave-sensitive (color blindness, tritan) |
| 78600 | *Pde6h* | 6 | ENSMUSG00000064330 | phosphodiesterase 6H, cGMP-specific, cone, gamma |
| 109648 | *Npy* | 6 | ENSMUSG00000029819 | neuropeptide Y |
| 11517 | *Adcyap1r1* | 6 | ENSMUSG00000029778 | adenylate cyclase activating polypeptide 1 receptor 1 |
| 50501 | *Prok2* | 6 | ENSMUSG00000030069 | prokineticin 2 |
| 16846 | *Lep* | 6 | ENSMUSG00000059201 | leptin |
| 12922 | *Crhr2* | 6 | ENSMUSG00000003476 | corticotropin releasing hormone receptor 2 |
| 21802 | *Tgfa* | 6 | ENSMUSG00000029999 | transforming growth factor alpha |
| 50799 | *Slc25a13* | 6 | ENSMUSG00000015112 | solute carrier family 25 (mitochondrial carrier, adenine nucleotide translocator), member 13 |
| 11865 | *Arntl* | 7 | ENSMUSG00000055116 | aryl hydrocarbon receptor nuclear translocator-like |
| 108071 | *Grm5* | 7 | ENSMUSG00000049583 | glutamate receptor, metabotropic 5 |
| 26417 | *Mapk3* | 7 | ENSMUSG00000063065 | mitogen-activated protein kinase 3 |
| 18751 | *Prkcb* | 7 | ENSMUSG00000052889 | protein kinase C, beta |
| 18752 | *Prkcg* | 7 | ENSMUSG00000078816 | protein kinase C, gamma |
| 16890 | *Lipe* | 7 | ENSMUSG00000003123 | lipase, hormone sensitive |
| 13491 | *Drd4* | 7 | ENSMUSG00000025496 | dopamine receptor D4 |
| 21823 | *Th* | 7 | ENSMUSG00000000214 | tyrosine hydroxylase |
| 232807 | *Ppp1r12c* | 7 | ENSMUSG00000019254 | protein phosphatase 1, regulatory (inhibitor) subunit 12C |
| 170460 | *Stard5* | 7 | ENSMUSG00000046027 | StAR-related lipid transfer (START) domain containing 5 |
| 11441 | *Chrna7* | 7 | ENSMUSG00000030525 | cholinergic receptor, nicotinic, alpha polypeptide 7 |
| 319520 | *Dusp4* | 8 | ENSMUSG00000031530 | dual specificity phosphatase 4 |
| 16477 | *Junb* | 8 | ENSMUSG00000052837 | Jun-B oncogene |
| 56437 | *Rrad* | 8 | ENSMUSG00000031880 | Ras-related associated with diabetes |
| 20498 | *Slc12a4* | 8 | ENSMUSG00000017765 | solute carrier family 12, member 4 |
| 18747 | *Prkaca* | 8 | ENSMUSG00000005469 | protein kinase, cAMP dependent, catalytic, alpha |
| 19414 | *Rasa3* | 8 | ENSMUSG00000031453 | RAS p21 protein activator 3 |
| 20845 | *Star* | 8 | ENSMUSG00000031574 | steroidogenic acute regulatory protein |
| 110784 | *Nr3c2* | 8 | ENSMUSG00000031618 | nuclear receptor subfamily 3, group C, member 2 |
| 17773 | *Mtnr1a* | 8 | ENSMUSG00000054764 | melatonin receptor 1A |
| 14681 | *Gnao1* | 8 | ENSMUSG00000031748 | guanine nucleotide binding protein, alpha O |
| 19883 | *Rora* | 9 | ENSMUSG00000032238 | RAR-related orphan receptor alpha |
| 23806 | *Arih1* | 9 | ENSMUSG00000025234 | ariadne ubiquitin-conjugating enzyme E2 binding protein homolog 1 (Drosophila) |
| 215418 | *Csrnp1* | 9 | ENSMUSG00000032515 | cysteine-serine-rich nuclear protein 1 |
| 75717 | *Cul5* | 9 | ENSMUSG00000032030 | cullin 5 |
| 14802 | *Gria4* | 9 | ENSMUSG00000025892 | glutamate receptor, ionotropic, AMPA4 (alpha 4) |
| 26395 | *Map2k1* | 9 | ENSMUSG00000004936 | mitogen-activated protein kinase kinase 1 |
| 114713 | *Rasa2* | 9 | ENSMUSG00000032413 | RAS p21 protein activator 2 |
| 14685 | *Gnat1* | 9 | ENSMUSG00000034837 | guanine nucleotide binding protein, alpha transducing 1 |
| 15551 | *Htr1b* | 9 | ENSMUSG00000049511 | 5-hydroxytryptamine (serotonin) receptor 1B |
| 16835 | *Ldlr* | 9 | ENSMUSG00000032193 | low density lipoprotein receptor |
| 13070 | *Cyp11a1* | 9 | ENSMUSG00000032323 | cytochrome P450, family 11, subfamily a, polypeptide 1 |
| 13075 | *Cyp19a1* | 9 | ENSMUSG00000032274 | cytochrome P450, family 19, subfamily a, polypeptide 1 |
| 110835 | *Chrna5* | 9 | ENSMUSG00000035594 | cholinergic receptor, nicotinic, alpha polypeptide 5 |
| 108015 | *Chrnb4* | 9 | ENSMUSG00000035200 | cholinergic receptor, nicotinic, beta polypeptide 4 |
| 12209 | *Brs3* | X | ENSMUSG00000031130 | bombesin-like receptor 3 |
| 14829 | *Grpr* | X | ENSMUSG00000031364 | gastrin releasing peptide receptor |
| 14539 | *Opn1mw* | X | ENSMUSG00000031394 | opsin 1 (cone pigments), medium-wave-sensitive (color blindness, deutan) |
| 20164 | *Opn1lw* | X | NA | opsin 1 (cone pigments), long-wave-sensitive |
| 107626 | *Asmt* | X | ENSMUSG00000093806 | acetylserotonin O-methyltransferase |
| 17161 | *Maoa* | X | ENSMUSG00000025037 | monoamine oxidase A |
| 11609 | *Agtr2* | X | ENSMUSG00000068122 | angiotensin II receptor, type 2 |
| 11740 | *Slc25a5* | X | ENSMUSG00000016319 | solute carrier family 25 (mitochondrial carrier, adenine nucleotide translocator), member 5 |
| 13170 | *Dbp* | 7 | ENSMUSG00000059824 | D site albumin promoter binding protein |
| 21685 | *Tef* | 15 | ENSMUSG00000022389 | thyrotroph embryonic factor |
| 217082 | *Hlf* | 11 | ENSMUSG00000003949 | hepatic leukemia factor |
| 56183 | *Nmu* | 5 | ENSMUSG00000029236 | neuromedin U |
| 216749 | *Nmur2* | 11 | ENSMUSG00000037393 | neuromedin U receptor 2 |
| 14767 | *Nmur1* | 1 | ENSMUSG00000026237 | neuromedin U receptor 1 |
| 21926 | *Tnf* | 17 | ENSMUSG00000024401 | tumor necrosis factor |
| 93759 | *Sirt1* | 10 | ENSMUSG00000020063 | sirtuin 1 |
| 26361 | *Avpr1b* | 1 | ENSMUSG00000026432 | arginine vasopressin receptor 1B |
| 18030 | *Nfil3* | 13 | ENSMUSG00000056749 | nuclear factor, interleukin 3, regulated |
| 56708 | *Clcf1* | 19 | ENSMUSG00000040663 | cardiotrophin-like cytokine factor 1 |
| 16531 | *Kcnma1* | 7 | ENSMUSG00000063142 | potassium large conductance calcium-activated channel, subfamily M, alpha member 1 |
| 12000 | *Avpr2* | X | ENSMUSG00000031390 | arginine vasopressin receptor 2 |
| 14415 | *Gad1* | 2 | ENSMUSG00000070880 | glutamate decarboxylase 1 |
| 14417 | *Gad2* | 2 | ENSMUSG00000026787 | glutamic acid decarboxylase 2 |
| 11614 | *Nr0b1* | X | ENSMUSG00000025056 | nuclear receptor subfamily 0, group B, member 1 |
| 13121 | *Cyp51a1* | 5 | ENSMUSG00000001467 | cytochrome P450, family 51 |
| 53610 | *Nono* | X | ENSMUSG00000031311 | non-POU-domain-containing, octamer binding protein |
| 21853 | *Timeless* | 10 | ENSMUSG00000039994 | timeless circadian clock 1 |
| 13122 | *Cyp7a1* | 4 | ENSMUSG00000028240 | cytochrome P450, family 7, subfamily a, polypeptide 1 |
| 19013 | *Ppara* | 15 |  | peroxisome proliferator activated receptor alpha |
| 19017 | *Ppargc1a* | 5 | ENSMUSG00000029167 | peroxisome proliferative activated receptor, gamma, coactivator 1 alpha |
| 13124 | *Cyp8b1* | 9 | ENSMUSG00000050445 | cytochrome P450, family 8, subfamily b, polypeptide 1 |
| 56050 | *Cyp39a1* | 17 | ENSMUSG00000023963 | cytochrome P450, family 39, subfamily a, polypeptide 1 |
| 104086 | *Cyp27a1* | 1 | ENSMUSG00000026170 | cytochrome P450, family 27, subfamily a, polypeptide 1 |
| 12355 | *Nr1i3* | 1 | ENSMUSG00000005677 | nuclear receptor subfamily 1, group I, member 3 |
| 15357 | *Hmgcr* | 13 | ENSMUSG00000021670 | 3-hydroxy-3-methylglutaryl-Coenzyme A reductase |
| 20181 | *Rxra* | 2 | ENSMUSG00000015846 | retinoid X receptor alpha |
| 13072 | *Cyp11b2* | 15 | ENSMUSG00000022589 | cytochrome P450, family 11, subfamily b, polypeptide 2 |

**Supplementary Table S8: Single nucleotide variants (SNVs) discovered by whole exome sequencing.**

A list of SNVs discovered to be different between the two strains in the genes whose circadian expression patterns were affected by genotype.

| **Location** | **Gene** | | **Type of SNV** | | **Amino acid change** | | **Chr** | | **Chr. position** | | **Ref** | | **Obs** | |  |
| --- | --- | --- | --- | --- | --- | --- | --- | --- | --- | --- | --- | --- | --- | --- | --- |
| intronic | | *Per2* | |  | |  | | chr1 | | 93324200 | | T | | C | |
| intronic | | *Per2* | |  | |  | | chr1 | | 93324254 | | T | | C | |
| exonic | | *Per2* | | synonymous SNV | | NM_011066:c.C2109T:p.G703G | | chr1 | | 93324503 | | G | | A | |
| exonic | | *Per2* | | synonymous SNV | | NM_011066:c.G2061A:p.T687T | | chr1 | | 93324551 | | C | | T | |
| intronic | | *Per2* | |  | |  | | chr1 | | 93327298 | | T | | C | |
| intronic | | *Per2* | |  | |  | | chr1 | | 93329485 | | T | | C | |
| intronic | | *Per2* | |  | |  | | chr1 | | 93329572 | | G | | A | |
| exonic | | *Per2* | | synonymous SNV | | NM_011066:c.A1401G:p.L467L | | chr1 | | 93331208 | | T | | C | |
| intronic | | *Per2* | |  | |  | | chr1 | | 93332405 | | A | | G | |
| intronic | | *Per2* | |  | |  | | chr1 | | 93334844 | | C | | T | |
| intronic | | *Per2* | |  | |  | | chr1 | | 93337174 | | G | | A | |
| intronic | | *Per2* | |  | |  | | chr1 | | 93337211 | | T | | C | |
| exonic | | *Per2* | | nonsynonymous SNV | | NM_011066:c.G515C:p.S172T | | chr1 | | 93342127 | | C | | G | |
| intronic | | *Nr1i3* | |  | |  | | chr1 | | 173144355 | | G | | A | |
| intronic | | *Nr1i3* | |  | |  | | chr1 | | 173144361 | | G | | T | |
| intronic | | *Nr1i3* | |  | |  | | chr1 | | 173144441 | | T | | C | |
| intronic | | *Nr1i3* | |  | |  | | chr1 | | 173144864 | | T | | C | |
| intronic | | *Nr1i3* | |  | |  | | chr1 | | 173144873 | | C | | G | |
| intronic | | *Nr1i3* | |  | |  | | chr1 | | 173144946 | | C | | A | |
| intronic | | *Nr1i3* | |  | |  | | chr1 | | 173144995 | | T | | C | |
| intronic | | *Nr1i3* | |  | |  | | chr1 | | 173145003 | | T | | C | |
| intronic | | *Nr1i3* | |  | |  | | chr1 | | 173145278 | | A | | G | |
| intronic | | *Nr1i3* | |  | |  | | chr1 | | 173146350 | | A | | T | |
| intronic | | *Nr1i3* | |  | |  | | chr1 | | 173146358 | | T | | C | |
| intronic | | *Nr1i3* | |  | |  | | chr1 | | 173146473 | | G | | T | |
| exonic | | *Nr1i3* | | synonymous SNV | | NM_001243062:c.G384C:p.L128L | | chr1 | | 173146625 | | G | | C | |
| intronic | | *Nr1i3* | |  | |  | | chr1 | | 173146807 | | T | | C | |
| exonic | | *Nr1i3* | | synonymous SNV | | NM_001243062:c.G444A:p.P148P | | chr1 | | 173146882 | | G | | A | |
| intronic | | *Per1* | |  | |  | | chr11 | | 68919241 | | T | | C | |
| intronic | | *Per1* | |  | |  | | chr11 | | 68920580 | | C | | T | |
| intronic | | *Per1* | |  | |  | | chr11 | | 68920968 | | C | | G | |
| exonic | | *Per1* | | synonymous SNV | | NM_001159367:c.C3138A:p.L1046L | | chr11 | | 68921103 | | C | | A | |
| intronic | | *Per1* | |  | |  | | chr11 | | 68921235 | | T | | G | |
| intronic | | *Per1* | |  | |  | | chr11 | | 68921725 | | A | | G | |
| intronic | | *Per1* | |  | |  | | chr11 | | 68921745 | | G | | A | |
| exonic | | *Per1* | | synonymous SNV | | NM_001159367:c.C3651G:p.P1217P | | chr11 | | 68922636 | | C | | G | |
| exonic | | *Per1* | | synonymous SNV | | NM_001159367:c.T3732C:p.G1244G | | chr11 | | 68922717 | | T | | C | |
| intronic | | *Ppara* | |  | |  | | chr15 | | 85608256 | | C | | T | |
| intronic | | *Ppara* | |  | |  | | chr15 | | 85619638 | | T | | C | |
| intronic | | *Ppara* | |  | |  | | chr15 | | 85621215 | | G | | A | |
| intergenic | | *Cyp21a1*  *(dist=1868)* | |  | |  | | chr17 | | 34936425 | | T | | C | |
| intronic | | *Cyp17a1* | |  | |  | | chr19 | | 46742116 | | G | | A | |
| intronic | | *Cyp7a1* | |  | |  | | chr4 | | 6198168 | | G | | T | |
| intronic | | *Cyp7a1* | |  | |  | | chr4 | | 6198196 | | G | | A | |
| intronic | | *Cyp7a1* | |  | |  | | chr4 | | 6198212 | | G | | A | |
| exonic | | *Cyp7a1* | | nonsynonymous SNV | | NM_007824:c.G952T:p.A318S | | chr4 | | 6198350 | | C | | A | |
| exonic | | *Cyp7a1* | | synonymous SNV | | NM_007824:c.C840T:p.L280L | | chr4 | | 6199519 | | G | | A | |
| exonic | | *Cyp7a1* | | synonymous SNV | | NM_007824:c.T795C:p.D265D | | chr4 | | 6199564 | | A | | G | |
| exonic | | *Cyp7a1* | | nonsynonymous SNV | | NM_007824:c.T589A:p.S197T | | chr4 | | 6199770 | | A | | T | |
| intronic | | *Cyp7a1* | |  | |  | | chr4 | | 6200070 | | G | | A | |
| exonic | | *Cyp7a1* | | synonymous SNV | | NM_007824:c.T195C:p.H65H | | chr4 | | 6200857 | | A | | G | |
| intronic | | *Cyp7a1* | |  | |  | | chr4 | | 6201025 | | G | | C | |
| UTR3 | | *Cyp51* | |  | |  | | chr5 | | 4083072 | | T | | C | |
| intronic | | *Cyp51* | |  | |  | | chr5 | | 4091805 | | A | | C | |
| intergenic | | *Ppargc1a* | | *(dist=401548)* | |  | | chr5 | | 51443940 | | T | | C | |
| intergenic | | *Ppargc1a* | | *(dist=401471)* | |  | | chr5 | | 51444017 | | A | | G | |
| intergenic | | *Ppargc1a* | | *(dist=401416)* | |  | | chr5 | | 51444072 | | G | | A | |
| intronic | | *Ppargc1a* | |  | |  | | chr5 | | 51852464 | | C | | T | |
| exonic | | *Ppargc1a* | | nonsynonymous SNV | | NM_008904:c.G2024A:p.R675H | | chr5 | | 51854559 | | C | | T | |
| intronic | | *Ppargc1a* | |  | |  | | chr5 | | 51863788 | | A | | G | |
| intronic | | *Ppargc1a* | |  | |  | | chr5 | | 51863966 | | T | | C | |
| intronic | | *Ppargc1a* | |  | |  | | chr5 | | 51865708 | | T | | G | |
| intronic | | *Ppargc1a* | |  | |  | | chr5 | | 51881550 | | A | | G | |
| intronic | | *Ppargc1a* | |  | |  | | chr5 | | 51885794 | | T | | C | |
| intronic | | *Ppargc1a* | |  | |  | | chr5 | | 51886829 | | T | | A | |
| intronic | | *Ppargc1a* | |  | |  | | chr5 | | 51889088 | | A | | G | |
| exonic | | *Ppargc1a* | | synonymous SNV | | NM_008904:c.T267C:p.V89V | | chr5 | | 51889429 | | A | | G | |
| intronic | | *Ppargc1a* | |  | |  | | chr5 | | 51939709 | | T | | C | |
| intronic | | *Ppargc1a* | |  | |  | | chr5 | | 51939725 | | C | | T | |
| intronic | | *Ppargc1a* | |  | |  | | chr5 | | 51939743 | | A | | G | |
| upstream | | *Ppargc1a* | |  | |  | | chr5 | | 51945249 | | A | | G | |
| intergenic | | *Arntl*  *(dist=235162)* | |  | |  | | chr7 | | 120115817 | | T | | C | |
| intergenic | | *Arntl*  *(dist=235120)* | |  | |  | | chr7 | | 120115859 | | G | | A | |
| intronic | | *Arntl* | |  | |  | | chr7 | | 120384571 | | G | | A | |
| intronic | | *Arntl* | |  | |  | | chr7 | | 120384931 | | G | | T | |
| intronic | | *Arntl* | |  | |  | | chr7 | | 120386575 | | A | | C | |
| intronic | | *Arntl* | |  | |  | | chr7 | | 120386615 | | T | | C | |
| intronic | | *Arntl* | |  | |  | | chr7 | | 120447747 | | C | | T | |
| exonic | | *Arntl* | | synonymous SNV | | NM_001243048:c.C1404T:p.V468V | | chr7 | | 120449560 | | C | | T | |
| intronic | | *Arntl* | |  | |  | | chr7 | | 120449694 | | C | | T | |
| intronic | | *Arntl* | |  | |  | | chr7 | | 120452055 | | G | | C | |
| intronic | | *Arntl* | |  | |  | | chr7 | | 120452070 | | C | | T | |
| intronic | | *Arntl* | |  | |  | | chr7 | | 120452250 | | G | | C | |
| intronic | | *Arntl* | |  | |  | | chr7 | | 120456443 | | G | | A | |
| UTR5 | | *Dbp* | |  | |  | | chr7 | | 52960865 | | C | | T | |
| exonic | | *Dbp* | | synonymous SNV | | NM_016974:c.C66T:p.P22P | | chr7 | | 52961059 | | C | | T | |
| intronic | | *Dbp* | |  | |  | | chr7 | | 52963542 | | T | | C | |

**Supplementary Table S9**

A list of SNVs present in the insulin signaling pathway genes.

The table is appended as an additional Supplementary file with the name: **Table S9.xlsx**

**Supplementary Table S10**

A list of SNPs present in binding regions of core clock proteins. Data on genomic location of binding sites was taken from Koike et al and data on SNPs was taken from Imputed Mouse SNP resource.

| **SNV position** | **Confidence** | **Chr** | **CS_Start** | **CS_End** | **Gene** | **ChipSeq** | **Strain** |
| --- | --- | --- | --- | --- | --- | --- | --- |
| 68908310 | 2 | chr11 | 68908211 | 68908501 | *Per1* | BMAL1 | 129P1 |
| 68908614 | 2 | chr11 | 68908511 | 68908830 | *Per1* | BMAL1 | 129P1 |
| 68908620 | 2 | chr11 | 68908511 | 68908830 | *Per1* | BMAL1 | 129P1 |
| 68908750 | 2 | chr11 | 68908511 | 68908830 | *Per1* | BMAL1 | 129P1 |
| 68908813 | 2 | chr11 | 68908511 | 68908830 | *Per1* | BMAL1 | 129P1 |
| 98644843 | 2 | chr11 | 98644571 | 98645021 | *Nr1d1* | BMAL1 | 129P1 |
| 98644918 | 2 | chr11 | 98644571 | 98645021 | *Nr1d1* | BMAL1 | 129P1 |
| 98637109 | 2 | chr11 | 98636841 | 98637181 | *Nr1d1* | BMAL1 | 129P1 |
| 98634168 | 2 | chr11 | 98634141 | 98634561 | *Nr1d1* | BMAL1 | 129P1 |
| 98643374 | 2 | chr11 | 98643181 | 98643480 | *Nr1d1* | BMAL1 | 129P1 |
| 98643452 | 2 | chr11 | 98643181 | 98643480 | *Nr1d1* | BMAL1 | 129P1 |
| 98643456 | 1 | chr11 | 98643181 | 98643480 | *Nr1d1* | BMAL1 | 129P1 |
| 93355989 | 2 | chr1 | 93355801 | 93356370 | *Per2* | BMAL1 | 129P1 |
| 93356260 | 2 | chr1 | 93355801 | 93356370 | *Per2* | BMAL1 | 129P1 |
| 93356260 | 2 | chr1 | 93356181 | 93356600 | *Per2* | BMAL1 | 129P1 |
| 93356397 | 2 | chr1 | 93356181 | 93356600 | *Per2* | BMAL1 | 129P1 |
| 150419182 | 2 | chr4 | 150418941 | 150419210 | *Per3* | BMAL1 | 129P1 |
| 150418904 | 2 | chr4 | 150418731 | 150418911 | *Per3* | BMAL1 | 129P1 |
| 27480259 | 2 | chr2 | 27480231 | 27480580 | *Rxra* | BMAL1 | 129P1 |
| 46716220 | 2 | chr19 | 46716101 | 46716281 | *Cyp17a1* | BMAL1 | 129P1 |
| 51943955 | 2 | chr5 | 51943791 | 51944090 | *Ppargc1a* | BMAL1 | 129P1 |
| 68908614 | 2 | chr11 | 68908511 | 68908970 | *Per1* | CLOCK | 129P1 |
| 68908620 | 2 | chr11 | 68908511 | 68908970 | *Per1* | CLOCK | 129P1 |
| 68908750 | 2 | chr11 | 68908511 | 68908970 | *Per1* | CLOCK | 129P1 |
| 68908813 | 2 | chr11 | 68908511 | 68908970 | *Per1* | CLOCK | 129P1 |
| 68908960 | 2 | chr11 | 68908511 | 68908970 | *Per1* | CLOCK | 129P1 |
| 68908968 | 2 | chr11 | 68908511 | 68908970 | *Per1* | CLOCK | 129P1 |
| 68908310 | 2 | chr11 | 68908231 | 68908501 | *Per1* | CLOCK | 129P1 |
| 98637109 | 2 | chr11 | 98636441 | 98637151 | *Nr1d1* | CLOCK | 129P1 |
| 98644565 | 2 | chr11 | 98644521 | 98645140 | *Nr1d1* | CLOCK | 129P1 |
| 98644843 | 2 | chr11 | 98644521 | 98645140 | *Nr1d1* | CLOCK | 129P1 |
| 98644918 | 2 | chr11 | 98644521 | 98645140 | *Nr1d1* | CLOCK | 129P1 |
| 98644508 | 2 | chr11 | 98644421 | 98644671 | *Nr1d1* | CLOCK | 129P1 |
| 98644565 | 2 | chr11 | 98644421 | 98644671 | *Nr1d1* | CLOCK | 129P1 |
| 93355989 | 2 | chr1 | 93355841 | 93356161 | *Per2* | CLOCK | 129P1 |
| 93356260 | 2 | chr1 | 93356221 | 93356440 | *Per2* | CLOCK | 129P1 |
| 93356397 | 2 | chr1 | 93356221 | 93356440 | *Per2* | CLOCK | 129P1 |
| 46716220 | 2 | chr19 | 46716061 | 46716420 | *Cyp17a1* | CLOCK | 129P1 |
| 51943736 | 2 | chr5 | 51943691 | 51944020 | *Ppargc1a* | CLOCK | 129P1 |
| 51943955 | 2 | chr5 | 51943691 | 51944020 | *Ppargc1a* | CLOCK | 129P1 |
| 68908614 | 2 | chr11 | 68908501 | 68908880 | *Per1* | PER1 | 129P1 |
| 68908620 | 2 | chr11 | 68908501 | 68908880 | *Per1* | PER1 | 129P1 |
| 68908750 | 2 | chr11 | 68908501 | 68908880 | *Per1* | PER1 | 129P1 |
| 68908813 | 2 | chr11 | 68908501 | 68908880 | *Per1* | PER1 | 129P1 |
| 68908310 | 2 | chr11 | 68908231 | 68908471 | *Per1* | PER1 | 129P1 |
| 98637109 | 2 | chr11 | 98636511 | 98637140 | *Nr1d1* | PER1 | 129P1 |
| 98644508 | 2 | chr11 | 98644481 | 98645140 | *Nr1d1* | PER1 | 129P1 |
| 98644565 | 2 | chr11 | 98644481 | 98645140 | *Nr1d1* | PER1 | 129P1 |
| 98644843 | 2 | chr11 | 98644481 | 98645140 | *Nr1d1* | PER1 | 129P1 |
| 98644918 | 2 | chr11 | 98644481 | 98645140 | *Nr1d1* | PER1 | 129P1 |
| 93355989 | 2 | chr1 | 93355871 | 93356151 | *Per2* | PER1 | 129P1 |
| 46716220 | 2 | chr19 | 46716091 | 46716440 | *Cyp17a1* | PER1 | 129P1 |
| 46720943 | 2 | chr19 | 46720841 | 46721100 | *Cyp17a1* | PER1 | 129P1 |
| 68908614 | 2 | chr11 | 68908521 | 68908990 | *Per1* | PER2 | 129P1 |
| 68908620 | 2 | chr11 | 68908521 | 68908990 | *Per1* | PER2 | 129P1 |
| 68908750 | 2 | chr11 | 68908521 | 68908990 | *Per1* | PER2 | 129P1 |
| 68908813 | 2 | chr11 | 68908521 | 68908990 | *Per1* | PER2 | 129P1 |
| 68908960 | 2 | chr11 | 68908521 | 68908990 | *Per1* | PER2 | 129P1 |
| 68908968 | 2 | chr11 | 68908521 | 68908990 | *Per1* | PER2 | 129P1 |
| 68908310 | 2 | chr11 | 68908161 | 68908531 | *Per1* | PER2 | 129P1 |
| 98637109 | 2 | chr11 | 98636471 | 98637170 | *Nr1d1* | PER2 | 129P1 |
| 98644843 | 2 | chr11 | 98644661 | 98645150 | *Nr1d1* | PER2 | 129P1 |
| 98644918 | 2 | chr11 | 98644661 | 98645150 | *Nr1d1* | PER2 | 129P1 |
| 98634168 | 2 | chr11 | 98634141 | 98634530 | *Nr1d1* | PER2 | 129P1 |
| 98644508 | 2 | chr11 | 98644501 | 98644651 | *Nr1d1* | PER2 | 129P1 |
| 98644565 | 2 | chr11 | 98644501 | 98644651 | *Nr1d1* | PER2 | 129P1 |
| 93355989 | 2 | chr1 | 93355831 | 93356161 | *Per2* | PER2 | 129P1 |
| 93356260 | 2 | chr1 | 93356201 | 93356470 | *Per2* | PER2 | 129P1 |
| 93356397 | 2 | chr1 | 93356201 | 93356470 | *Per2* | PER2 | 129P1 |
| 93333537 | 2 | chr1 | 93333251 | 93333610 | *Per2* | PER2 | 129P1 |
| 150419182 | 2 | chr4 | 150418961 | 150419360 | *Per3* | PER2 | 129P1 |
| 150419349 | 2 | chr4 | 150418961 | 150419360 | *Per3* | PER2 | 129P1 |
| 150417652 | 2 | chr4 | 150417461 | 150417711 | *Per3* | PER2 | 129P1 |
| 46720828 | 2 | chr19 | 46720731 | 46721050 | *Cyp17a1* | PER2 | 129P1 |
| 46720829 | 2 | chr19 | 46720731 | 46721050 | *Cyp17a1* | PER2 | 129P1 |
| 46720943 | 2 | chr19 | 46720731 | 46721050 | *Cyp17a1* | PER2 | 129P1 |
| 46716220 | 2 | chr19 | 46716131 | 46716430 | *Cyp17a1* | PER2 | 129P1 |
| 120350590 | 2 | chr7 | 120350461 | 120351050 | *Arntl* | CRY1 | 129P1 |
| 68908310 | 2 | chr11 | 68907811 | 68908521 | *Per1* | CRY1 | 129P1 |
| 68908614 | 2 | chr11 | 68908531 | 68908941 | *Per1* | CRY1 | 129P1 |
| 68908620 | 2 | chr11 | 68908531 | 68908941 | *Per1* | CRY1 | 129P1 |
| 68908750 | 2 | chr11 | 68908531 | 68908941 | *Per1* | CRY1 | 129P1 |
| 68908813 | 2 | chr11 | 68908531 | 68908941 | *Per1* | CRY1 | 129P1 |
| 98637109 | 2 | chr11 | 98636401 | 98637420 | *Nr1d1* | CRY1 | 129P1 |
| 98644565 | 2 | chr11 | 98644521 | 98645050 | *Nr1d1* | CRY1 | 129P1 |
| 98644843 | 2 | chr11 | 98644521 | 98645050 | *Nr1d1* | CRY1 | 129P1 |
| 98644918 | 2 | chr11 | 98644521 | 98645050 | *Nr1d1* | CRY1 | 129P1 |
| 98634168 | 2 | chr11 | 98634161 | 98634611 | *Nr1d1* | CRY1 | 129P1 |
| 98644508 | 2 | chr11 | 98644481 | 98644651 | *Nr1d1* | CRY1 | 129P1 |
| 98644565 | 2 | chr11 | 98644481 | 98644651 | *Nr1d1* | CRY1 | 129P1 |
| 98636167 | 2 | chr11 | 98635971 | 98636171 | *Nr1d1* | CRY1 | 129P1 |
| 93355989 | 2 | chr1 | 93355741 | 93356191 | *Per2* | CRY1 | 129P1 |
| 93356260 | 2 | chr1 | 93356201 | 93356490 | *Per2* | CRY1 | 129P1 |
| 93356397 | 2 | chr1 | 93356201 | 93356490 | *Per2* | CRY1 | 129P1 |
| 93353564 | 2 | chr1 | 93353041 | 93353620 | *Per2* | CRY1 | 129P1 |
| 93352816 | 2 | chr1 | 93352511 | 93352900 | *Per2* | CRY1 | 129P1 |
| 93337174 | 2 | chr1 | 93336811 | 93337230 | *Per2* | CRY1 | 129P1 |
| 93337211 | 2 | chr1 | 93336811 | 93337230 | *Per2* | CRY1 | 129P1 |
| 93333537 | 2 | chr1 | 93333191 | 93333720 | *Per2* | CRY1 | 129P1 |
| 150418904 | 2 | chr4 | 150418811 | 150419271 | *Per3* | CRY1 | 129P1 |
| 150419182 | 2 | chr4 | 150418811 | 150419271 | *Per3* | CRY1 | 129P1 |
| 150417652 | 2 | chr4 | 150417501 | 150417850 | *Per3* | CRY1 | 129P1 |
| 150417822 | 2 | chr4 | 150417501 | 150417850 | *Per3* | CRY1 | 129P1 |
| 27490820 | 2 | chr2 | 27490731 | 27491100 | *Rxra* | CRY1 | 129P1 |
| 27490957 | 2 | chr2 | 27490731 | 27491100 | *Rxra* | CRY1 | 129P1 |
| 27490970 | 2 | chr2 | 27490731 | 27491100 | *Rxra* | CRY1 | 129P1 |
| 27490972 | 2 | chr2 | 27490731 | 27491100 | *Rxra* | CRY1 | 129P1 |
| 27490974 | 2 | chr2 | 27490731 | 27491100 | *Rxra* | CRY1 | 129P1 |
| 18068970 | 2 | chr3 | 18068881 | 18069180 | *Cyp7b1* | CRY1 | 129P1 |
| 18151353 | 2 | chr3 | 18151181 | 18151490 | *Cyp7b1* | CRY1 | 129P1 |
| 46716220 | 2 | chr19 | 46716091 | 46716450 | *Cyp17a1* | CRY1 | 129P1 |
| 46720828 | 2 | chr19 | 46720771 | 46721040 | *Cyp17a1* | CRY1 | 129P1 |
| 46720829 | 2 | chr19 | 46720771 | 46721040 | *Cyp17a1* | CRY1 | 129P1 |
| 46720943 | 2 | chr19 | 46720771 | 46721040 | *Cyp17a1* | CRY1 | 129P1 |
| 68908614 | 2 | chr11 | 68908521 | 68908920 | *Per1* | CRY2 | 129P1 |
| 68908620 | 2 | chr11 | 68908521 | 68908920 | *Per1* | CRY2 | 129P1 |
| 68908750 | 2 | chr11 | 68908521 | 68908920 | *Per1* | CRY2 | 129P1 |
| 68908813 | 2 | chr11 | 68908521 | 68908920 | *Per1* | CRY2 | 129P1 |
| 68908310 | 2 | chr11 | 68908221 | 68908511 | *Per1* | CRY2 | 129P1 |
| 98637109 | 2 | chr11 | 98636431 | 98637110 | *Nr1d1* | CRY2 | 129P1 |
| 98644843 | 2 | chr11 | 98644651 | 98645060 | *Nr1d1* | CRY2 | 129P1 |
| 98644918 | 2 | chr11 | 98644651 | 98645060 | *Nr1d1* | CRY2 | 129P1 |
| 98634168 | 2 | chr11 | 98634151 | 98634541 | *Nr1d1* | CRY2 | 129P1 |
| 98637109 | 2 | chr11 | 98636911 | 98637150 | *Nr1d1* | CRY2 | 129P1 |
| 98644508 | 2 | chr11 | 98644471 | 98644661 | *Nr1d1* | CRY2 | 129P1 |
| 98644565 | 2 | chr11 | 98644471 | 98644661 | *Nr1d1* | CRY2 | 129P1 |
| 93355989 | 2 | chr1 | 93355861 | 93356171 | *Per2* | CRY2 | 129P1 |
| 93356260 | 2 | chr1 | 93356181 | 93356411 | *Per2* | CRY2 | 129P1 |
| 93356397 | 2 | chr1 | 93356181 | 93356411 | *Per2* | CRY2 | 129P1 |
| 150419182 | 2 | chr4 | 150418961 | 150419191 | *Per3* | CRY2 | 129P1 |
| 27482311 | 2 | chr2 | 27482231 | 27482470 | *Rxra* | CRY2 | 129P1 |
| 27482313 | 2 | chr2 | 27482231 | 27482470 | *Rxra* | CRY2 | 129P1 |
| 27482370 | 2 | chr2 | 27482231 | 27482470 | *Rxra* | CRY2 | 129P1 |
| 46720828 | 2 | chr19 | 46720721 | 46721040 | *Cyp17a1* | CRY2 | 129P1 |
| 46720829 | 2 | chr19 | 46720721 | 46721040 | *Cyp17a1* | CRY2 | 129P1 |
| 46720943 | 2 | chr19 | 46720721 | 46721040 | *Cyp17a1* | CRY2 | 129P1 |
| 46716220 | 2 | chr19 | 46716111 | 46716590 | *Cyp17a1* | CRY2 | 129P1 |
| 68908614 | 2 | chr11 | 68908481 | 68908800 | *Per1* | NPAS2 | 129P1 |
| 68908620 | 2 | chr11 | 68908481 | 68908800 | *Per1* | NPAS2 | 129P1 |
| 68908750 | 2 | chr11 | 68908481 | 68908800 | *Per1* | NPAS2 | 129P1 |
| 68908310 | 2 | chr11 | 68908221 | 68908471 | *Per1* | NPAS2 | 129P1 |
| 98637109 | 2 | chr11 | 98636601 | 98637151 | *Nr1d1* | NPAS2 | 129P1 |
| 98644843 | 2 | chr11 | 98644621 | 98645190 | *Nr1d1* | NPAS2 | 129P1 |
| 98644918 | 2 | chr11 | 98644621 | 98645190 | *Nr1d1* | NPAS2 | 129P1 |
| 98637109 | 2 | chr11 | 98636841 | 98637150 | *Nr1d1* | NPAS2 | 129P1 |
| 98644918 | 2 | chr11 | 98644871 | 98645040 | *Nr1d1* | NPAS2 | 129P1 |
| 98644508 | 2 | chr11 | 98644481 | 98644871 | *Nr1d1* | NPAS2 | 129P1 |
| 98644565 | 2 | chr11 | 98644481 | 98644871 | *Nr1d1* | NPAS2 | 129P1 |
| 98644843 | 2 | chr11 | 98644481 | 98644871 | *Nr1d1* | NPAS2 | 129P1 |
| 93355989 | 2 | chr1 | 93355851 | 93356161 | *Per2* | NPAS2 | 129P1 |
| 93356260 | 2 | chr1 | 93356201 | 93356420 | *Per2* | NPAS2 | 129P1 |
| 93356397 | 2 | chr1 | 93356201 | 93356420 | *Per2* | NPAS2 | 129P1 |
| 68908310 | 2 | chr11 | 68908211 | 68908501 | *Per1* | BMAL1 | 129P3 |
| 68908614 | 2 | chr11 | 68908511 | 68908830 | *Per1* | BMAL1 | 129P3 |
| 68908620 | 2 | chr11 | 68908511 | 68908830 | *Per1* | BMAL1 | 129P3 |
| 68908750 | 2 | chr11 | 68908511 | 68908830 | *Per1* | BMAL1 | 129P3 |
| 68908813 | 2 | chr11 | 68908511 | 68908830 | *Per1* | BMAL1 | 129P3 |
| 98644843 | 2 | chr11 | 98644571 | 98645021 | *Nr1d1* | BMAL1 | 129P3 |
| 98644918 | 2 | chr11 | 98644571 | 98645021 | *Nr1d1* | BMAL1 | 129P3 |
| 98637109 | 2 | chr11 | 98636841 | 98637181 | *Nr1d1* | BMAL1 | 129P3 |
| 98634168 | 2 | chr11 | 98634141 | 98634561 | *Nr1d1* | BMAL1 | 129P3 |
| 98643374 | 2 | chr11 | 98643181 | 98643480 | *Nr1d1* | BMAL1 | 129P3 |
| 98643452 | 2 | chr11 | 98643181 | 98643480 | *Nr1d1* | BMAL1 | 129P3 |
| 98643456 | 1 | chr11 | 98643181 | 98643480 | *Nr1d1* | BMAL1 | 129P3 |
| 93355989 | 2 | chr1 | 93355801 | 93356370 | *Per2* | BMAL1 | 129P3 |
| 93356260 | 2 | chr1 | 93355801 | 93356370 | *Per2* | BMAL1 | 129P3 |
| 93356260 | 2 | chr1 | 93356181 | 93356600 | *Per2* | BMAL1 | 129P3 |
| 93356397 | 2 | chr1 | 93356181 | 93356600 | *Per2* | BMAL1 | 129P3 |
| 150419182 | 2 | chr4 | 150418941 | 150419210 | *Per3* | BMAL1 | 129P3 |
| 150418904 | 2 | chr4 | 150418731 | 150418911 | *Per3* | BMAL1 | 129P3 |
| 27480259 | 2 | chr2 | 27480231 | 27480580 | *Rxra* | BMAL1 | 129P3 |
| 46716220 | 2 | chr19 | 46716101 | 46716281 | *Cyp17a1* | BMAL1 | 129P3 |
| 51943955 | 2 | chr5 | 51943791 | 51944090 | *Ppargc1a* | BMAL1 | 129P3 |
| 68908614 | 2 | chr11 | 68908511 | 68908970 | *Per1* | CLOCK | 129P3 |
| 68908620 | 2 | chr11 | 68908511 | 68908970 | *Per1* | CLOCK | 129P3 |
| 68908750 | 2 | chr11 | 68908511 | 68908970 | *Per1* | CLOCK | 129P3 |
| 68908813 | 2 | chr11 | 68908511 | 68908970 | *Per1* | CLOCK | 129P3 |
| 68908960 | 2 | chr11 | 68908511 | 68908970 | *Per1* | CLOCK | 129P3 |
| 68908968 | 2 | chr11 | 68908511 | 68908970 | *Per1* | CLOCK | 129P3 |
| 68908310 | 2 | chr11 | 68908231 | 68908501 | *Per1* | CLOCK | 129P3 |
| 98637109 | 2 | chr11 | 98636441 | 98637151 | *Nr1d1* | CLOCK | 129P3 |
| 98644565 | 2 | chr11 | 98644521 | 98645140 | *Nr1d1* | CLOCK | 129P3 |
| 98644843 | 2 | chr11 | 98644521 | 98645140 | *Nr1d1* | CLOCK | 129P3 |
| 98644918 | 2 | chr11 | 98644521 | 98645140 | *Nr1d1* | CLOCK | 129P3 |
| 98644508 | 2 | chr11 | 98644421 | 98644671 | *Nr1d1* | CLOCK | 129P3 |
| 98644565 | 2 | chr11 | 98644421 | 98644671 | *Nr1d1* | CLOCK | 129P3 |
| 93355989 | 2 | chr1 | 93355841 | 93356161 | *Per2* | CLOCK | 129P3 |
| 93356260 | 2 | chr1 | 93356221 | 93356440 | *Per2* | CLOCK | 129P3 |
| 93356397 | 2 | chr1 | 93356221 | 93356440 | *Per2* | CLOCK | 129P3 |
| 46716220 | 2 | chr19 | 46716061 | 46716420 | *Cyp17a1* | CLOCK | 129P3 |
| 51943736 | 2 | chr5 | 51943691 | 51944020 | *Ppargc1a* | CLOCK | 129P3 |
| 51943955 | 2 | chr5 | 51943691 | 51944020 | *Ppargc1a* | CLOCK | 129P3 |
| 68908614 | 2 | chr11 | 68908501 | 68908880 | *Per1* | PER1 | 129P3 |
| 68908620 | 2 | chr11 | 68908501 | 68908880 | *Per1* | PER1 | 129P3 |
| 68908750 | 2 | chr11 | 68908501 | 68908880 | *Per1* | PER1 | 129P3 |
| 68908813 | 2 | chr11 | 68908501 | 68908880 | *Per1* | PER1 | 129P3 |
| 68908310 | 2 | chr11 | 68908231 | 68908471 | *Per1* | PER1 | 129P3 |
| 98637109 | 2 | chr11 | 98636511 | 98637140 | *Nr1d1* | PER1 | 129P3 |
| 98644508 | 2 | chr11 | 98644481 | 98645140 | *Nr1d1* | PER1 | 129P3 |
| 98644565 | 2 | chr11 | 98644481 | 98645140 | *Nr1d1* | PER1 | 129P3 |
| 98644843 | 2 | chr11 | 98644481 | 98645140 | *Nr1d1* | PER1 | 129P3 |
| 98644918 | 2 | chr11 | 98644481 | 98645140 | *Nr1d1* | PER1 | 129P3 |
| 93355989 | 2 | chr1 | 93355871 | 93356151 | *Per2* | PER1 | 129P3 |
| 46716220 | 2 | chr19 | 46716091 | 46716440 | *Cyp17a1* | PER1 | 129P3 |
| 46720943 | 2 | chr19 | 46720841 | 46721100 | *Cyp17a1* | PER1 | 129P3 |
| 68908614 | 2 | chr11 | 68908521 | 68908990 | *Per1* | PER2 | 129P3 |
| 68908620 | 2 | chr11 | 68908521 | 68908990 | *Per1* | PER2 | 129P3 |
| 68908750 | 2 | chr11 | 68908521 | 68908990 | *Per1* | PER2 | 129P3 |
| 68908813 | 2 | chr11 | 68908521 | 68908990 | *Per1* | PER2 | 129P3 |
| 68908960 | 2 | chr11 | 68908521 | 68908990 | *Per1* | PER2 | 129P3 |
| 68908968 | 2 | chr11 | 68908521 | 68908990 | *Per1* | PER2 | 129P3 |
| 68908310 | 2 | chr11 | 68908161 | 68908531 | *Per1* | PER2 | 129P3 |
| 98637109 | 2 | chr11 | 98636471 | 98637170 | *Nr1d1* | PER2 | 129P3 |
| 98644843 | 2 | chr11 | 98644661 | 98645150 | *Nr1d1* | PER2 | 129P3 |
| 98644918 | 2 | chr11 | 98644661 | 98645150 | *Nr1d1* | PER2 | 129P3 |
| 98634168 | 2 | chr11 | 98634141 | 98634530 | *Nr1d1* | PER2 | 129P3 |
| 98644508 | 2 | chr11 | 98644501 | 98644651 | *Nr1d1* | PER2 | 129P3 |
| 98644565 | 2 | chr11 | 98644501 | 98644651 | *Nr1d1* | PER2 | 129P3 |
| 93355989 | 2 | chr1 | 93355831 | 93356161 | *Per2* | PER2 | 129P3 |
| 93356260 | 2 | chr1 | 93356201 | 93356470 | *Per2* | PER2 | 129P3 |
| 93356397 | 2 | chr1 | 93356201 | 93356470 | *Per2* | PER2 | 129P3 |
| 93333537 | 2 | chr1 | 93333251 | 93333610 | *Per2* | PER2 | 129P3 |
| 150419182 | 2 | chr4 | 150418961 | 150419360 | *Per3* | PER2 | 129P3 |
| 150419349 | 2 | chr4 | 150418961 | 150419360 | *Per3* | PER2 | 129P3 |
| 150417652 | 2 | chr4 | 150417461 | 150417711 | *Per3* | PER2 | 129P3 |
| 46720828 | 2 | chr19 | 46720731 | 46721050 | *Cyp17a1* | PER2 | 129P3 |
| 46720829 | 2 | chr19 | 46720731 | 46721050 | *Cyp17a1* | PER2 | 129P3 |
| 46720943 | 2 | chr19 | 46720731 | 46721050 | *Cyp17a1* | PER2 | 129P3 |
| 46716220 | 2 | chr19 | 46716131 | 46716430 | *Cyp17a1* | PER2 | 129P3 |
| 120350590 | 2 | chr7 | 120350461 | 120351050 | *Arntl* | CRY1 | 129P3 |
| 68908310 | 2 | chr11 | 68907811 | 68908521 | *Per1* | CRY1 | 129P3 |
| 68908614 | 2 | chr11 | 68908531 | 68908941 | *Per1* | CRY1 | 129P3 |
| 68908620 | 2 | chr11 | 68908531 | 68908941 | *Per1* | CRY1 | 129P3 |
| 68908750 | 2 | chr11 | 68908531 | 68908941 | *Per1* | CRY1 | 129P3 |
| 68908813 | 2 | chr11 | 68908531 | 68908941 | *Per1* | CRY1 | 129P3 |
| 98637109 | 2 | chr11 | 98636401 | 98637420 | *Nr1d1* | CRY1 | 129P3 |
| 98644565 | 2 | chr11 | 98644521 | 98645050 | *Nr1d1* | CRY1 | 129P3 |
| 98644843 | 2 | chr11 | 98644521 | 98645050 | *Nr1d1* | CRY1 | 129P3 |
| 98644918 | 2 | chr11 | 98644521 | 98645050 | *Nr1d1* | CRY1 | 129P3 |
| 98634168 | 2 | chr11 | 98634161 | 98634611 | *Nr1d1* | CRY1 | 129P3 |
| 98644508 | 2 | chr11 | 98644481 | 98644651 | *Nr1d1* | CRY1 | 129P3 |
| 98644565 | 2 | chr11 | 98644481 | 98644651 | *Nr1d1* | CRY1 | 129P3 |
| 98636167 | 2 | chr11 | 98635971 | 98636171 | *Nr1d1* | CRY1 | 129P3 |
| 93355989 | 2 | chr1 | 93355741 | 93356191 | *Per2* | CRY1 | 129P3 |
| 93356260 | 2 | chr1 | 93356201 | 93356490 | *Per2* | CRY1 | 129P3 |
| 93356397 | 2 | chr1 | 93356201 | 93356490 | *Per2* | CRY1 | 129P3 |
| 93353564 | 2 | chr1 | 93353041 | 93353620 | *Per2* | CRY1 | 129P3 |
| 93352816 | 2 | chr1 | 93352511 | 93352900 | *Per2* | CRY1 | 129P3 |
| 93337174 | 2 | chr1 | 93336811 | 93337230 | *Per2* | CRY1 | 129P3 |
| 93337211 | 2 | chr1 | 93336811 | 93337230 | *Per2* | CRY1 | 129P3 |
| 93333537 | 2 | chr1 | 93333191 | 93333720 | *Per2* | CRY1 | 129P3 |
| 150418904 | 2 | chr4 | 150418811 | 150419271 | *Per3* | CRY1 | 129P3 |
| 150419182 | 2 | chr4 | 150418811 | 150419271 | *Per3* | CRY1 | 129P3 |
| 150417652 | 2 | chr4 | 150417501 | 150417850 | *Per3* | CRY1 | 129P3 |
| 150417822 | 2 | chr4 | 150417501 | 150417850 | *Per3* | CRY1 | 129P3 |
| 27490820 | 2 | chr2 | 27490731 | 27491100 | *Rxra* | CRY1 | 129P3 |
| 27490957 | 2 | chr2 | 27490731 | 27491100 | *Rxra* | CRY1 | 129P3 |
| 27490970 | 2 | chr2 | 27490731 | 27491100 | *Rxra* | CRY1 | 129P3 |
| 27490972 | 2 | chr2 | 27490731 | 27491100 | *Rxra* | CRY1 | 129P3 |
| 27490974 | 2 | chr2 | 27490731 | 27491100 | *Rxra* | CRY1 | 129P3 |
| 18068970 | 2 | chr3 | 18068881 | 18069180 | *Cyp7b1* | CRY1 | 129P3 |
| 18151353 | 2 | chr3 | 18151181 | 18151490 | *Cyp7b1* | CRY1 | 129P3 |
| 46716220 | 2 | chr19 | 46716091 | 46716450 | *Cyp17a1* | CRY1 | 129P3 |
| 46720828 | 2 | chr19 | 46720771 | 46721040 | *Cyp17a1* | CRY1 | 129P3 |
| 46720829 | 2 | chr19 | 46720771 | 46721040 | *Cyp17a1* | CRY1 | 129P3 |
| 46720943 | 2 | chr19 | 46720771 | 46721040 | *Cyp17a1* | CRY1 | 129P3 |
| 68908614 | 2 | chr11 | 68908521 | 68908920 | *Per1* | CRY2 | 129P3 |
| 68908620 | 2 | chr11 | 68908521 | 68908920 | *Per1* | CRY2 | 129P3 |
| 68908750 | 2 | chr11 | 68908521 | 68908920 | *Per1* | CRY2 | 129P3 |
| 68908813 | 2 | chr11 | 68908521 | 68908920 | *Per1* | CRY2 | 129P3 |
| 68908310 | 2 | chr11 | 68908221 | 68908511 | *Per1* | CRY2 | 129P3 |
| 98637109 | 2 | chr11 | 98636431 | 98637110 | *Nr1d1* | CRY2 | 129P3 |
| 98644843 | 2 | chr11 | 98644651 | 98645060 | *Nr1d1* | CRY2 | 129P3 |
| 98644918 | 2 | chr11 | 98644651 | 98645060 | *Nr1d1* | CRY2 | 129P3 |
| 98634168 | 2 | chr11 | 98634151 | 98634541 | *Nr1d1* | CRY2 | 129P3 |
| 98637109 | 2 | chr11 | 98636911 | 98637150 | *Nr1d1* | CRY2 | 129P3 |
| 98644508 | 2 | chr11 | 98644471 | 98644661 | *Nr1d1* | CRY2 | 129P3 |
| 98644565 | 2 | chr11 | 98644471 | 98644661 | *Nr1d1* | CRY2 | 129P3 |
| 93355989 | 2 | chr1 | 93355861 | 93356171 | *Per2* | CRY2 | 129P3 |
| 93356260 | 2 | chr1 | 93356181 | 93356411 | *Per2* | CRY2 | 129P3 |
| 93356397 | 2 | chr1 | 93356181 | 93356411 | *Per2* | CRY2 | 129P3 |
| 150419182 | 2 | chr4 | 150418961 | 150419191 | *Per3* | CRY2 | 129P3 |
| 27482311 | 2 | chr2 | 27482231 | 27482470 | *Rxra* | CRY2 | 129P3 |
| 27482313 | 2 | chr2 | 27482231 | 27482470 | *Rxra* | CRY2 | 129P3 |
| 27482370 | 2 | chr2 | 27482231 | 27482470 | *Rxra* | CRY2 | 129P3 |
| 46720828 | 2 | chr19 | 46720721 | 46721040 | *Cyp17a1* | CRY2 | 129P3 |
| 46720829 | 2 | chr19 | 46720721 | 46721040 | *Cyp17a1* | CRY2 | 129P3 |
| 46720943 | 2 | chr19 | 46720721 | 46721040 | *Cyp17a1* | CRY2 | 129P3 |
| 46716220 | 2 | chr19 | 46716111 | 46716590 | *Cyp17a1* | CRY2 | 129P3 |
| 68908614 | 2 | chr11 | 68908481 | 68908800 | *Per1* | NPAS2 | 129P3 |
| 68908620 | 2 | chr11 | 68908481 | 68908800 | *Per1* | NPAS2 | 129P3 |
| 68908750 | 2 | chr11 | 68908481 | 68908800 | *Per1* | NPAS2 | 129P3 |
| 68908310 | 2 | chr11 | 68908221 | 68908471 | *Per1* | NPAS2 | 129P3 |
| 98637109 | 2 | chr11 | 98636601 | 98637151 | *Nr1d1* | NPAS2 | 129P3 |
| 98644843 | 2 | chr11 | 98644621 | 98645190 | *Nr1d1* | NPAS2 | 129P3 |
| 98644918 | 2 | chr11 | 98644621 | 98645190 | *Nr1d1* | NPAS2 | 129P3 |
| 98637109 | 2 | chr11 | 98636841 | 98637150 | *Nr1d1* | NPAS2 | 129P3 |
| 98644918 | 2 | chr11 | 98644871 | 98645040 | *Nr1d1* | NPAS2 | 129P3 |
| 98644508 | 2 | chr11 | 98644481 | 98644871 | *Nr1d1* | NPAS2 | 129P3 |
| 98644565 | 2 | chr11 | 98644481 | 98644871 | *Nr1d1* | NPAS2 | 129P3 |
| 98644843 | 2 | chr11 | 98644481 | 98644871 | *Nr1d1* | NPAS2 | 129P3 |
| 93355989 | 2 | chr1 | 93355851 | 93356161 | *Per2* | NPAS2 | 129P3 |
| 93356260 | 2 | chr1 | 93356201 | 93356420 | *Per2* | NPAS2 | 129P3 |
| 93356397 | 2 | chr1 | 93356201 | 93356420 | *Per2* | NPAS2 | 129P3 |
| 68908310 | 3 | chr11 | 68908211 | 68908501 | *Per1* | BMAL1 | 129S1 |
| 68908614 | 3 | chr11 | 68908511 | 68908830 | *Per1* | BMAL1 | 129S1 |
| 68908620 | 3 | chr11 | 68908511 | 68908830 | *Per1* | BMAL1 | 129S1 |
| 68908750 | 3 | chr11 | 68908511 | 68908830 | *Per1* | BMAL1 | 129S1 |
| 68908813 | 3 | chr11 | 68908511 | 68908830 | *Per1* | BMAL1 | 129S1 |
| 98644843 | 3 | chr11 | 98644571 | 98645021 | *Nr1d1* | BMAL1 | 129S1 |
| 98644918 | 3 | chr11 | 98644571 | 98645021 | *Nr1d1* | BMAL1 | 129S1 |
| 98637109 | 3 | chr11 | 98636841 | 98637181 | *Nr1d1* | BMAL1 | 129S1 |
| 98634168 | 3 | chr11 | 98634141 | 98634561 | *Nr1d1* | BMAL1 | 129S1 |
| 98643374 | 3 | chr11 | 98643181 | 98643480 | *Nr1d1* | BMAL1 | 129S1 |
| 98643452 | 3 | chr11 | 98643181 | 98643480 | *Nr1d1* | BMAL1 | 129S1 |
| 98643456 | 3 | chr11 | 98643181 | 98643480 | *Nr1d1* | BMAL1 | 129S1 |
| 93355989 | 3 | chr1 | 93355801 | 93356370 | *Per2* | BMAL1 | 129S1 |
| 93356260 | 3 | chr1 | 93355801 | 93356370 | *Per2* | BMAL1 | 129S1 |
| 93356260 | 3 | chr1 | 93356181 | 93356600 | *Per2* | BMAL1 | 129S1 |
| 93356397 | 3 | chr1 | 93356181 | 93356600 | *Per2* | BMAL1 | 129S1 |
| 150419182 | 3 | chr4 | 150418941 | 150419210 | *Per3* | BMAL1 | 129S1 |
| 150418904 | 3 | chr4 | 150418731 | 150418911 | *Per3* | BMAL1 | 129S1 |
| 27480259 | 3 | chr2 | 27480231 | 27480580 | *Rxra* | BMAL1 | 129S1 |
| 52960092 | 3 | chr7 | 52960041 | 52960760 | *Dbp* | BMAL1 | 129S1 |
| 52960117 | 3 | chr7 | 52960041 | 52960760 | *Dbp* | BMAL1 | 129S1 |
| 52960169 | 3 | chr7 | 52960041 | 52960760 | *Dbp* | BMAL1 | 129S1 |
| 52960311 | 3 | chr7 | 52960041 | 52960760 | *Dbp* | BMAL1 | 129S1 |
| 52960312 | 3 | chr7 | 52960041 | 52960760 | *Dbp* | BMAL1 | 129S1 |
| 52960341 | 3 | chr7 | 52960041 | 52960760 | *Dbp* | BMAL1 | 129S1 |
| 52960671 | 3 | chr7 | 52960041 | 52960760 | *Dbp* | BMAL1 | 129S1 |
| 52962826 | 3 | chr7 | 52962811 | 52963430 | *Dbp* | BMAL1 | 129S1 |
| 52963101 | 3 | chr7 | 52962811 | 52963430 | *Dbp* | BMAL1 | 129S1 |
| 52963127 | 3 | chr7 | 52962811 | 52963430 | *Dbp* | BMAL1 | 129S1 |
| 52963230 | 3 | chr7 | 52962811 | 52963430 | *Dbp* | BMAL1 | 129S1 |
| 52963234 | 3 | chr7 | 52962811 | 52963430 | *Dbp* | BMAL1 | 129S1 |
| 52963367 | 3 | chr7 | 52962811 | 52963430 | *Dbp* | BMAL1 | 129S1 |
| 52963415 | 3 | chr7 | 52962811 | 52963430 | *Dbp* | BMAL1 | 129S1 |
| 52963419 | 3 | chr7 | 52962811 | 52963430 | *Dbp* | BMAL1 | 129S1 |
| 52961354 | 3 | chr7 | 52961201 | 52961730 | *Dbp* | BMAL1 | 129S1 |
| 52961679 | 3 | chr7 | 52961201 | 52961730 | *Dbp* | BMAL1 | 129S1 |
| 52961720 | 3 | chr7 | 52961201 | 52961730 | *Dbp* | BMAL1 | 129S1 |
| 46716220 | 3 | chr19 | 46716101 | 46716281 | *Cyp17a1* | BMAL1 | 129S1 |
| 51943955 | 3 | chr5 | 51943791 | 51944090 | *Ppargc1a* | BMAL1 | 129S1 |
| 68908614 | 3 | chr11 | 68908511 | 68908970 | *Per1* | CLOCK | 129S1 |
| 68908620 | 3 | chr11 | 68908511 | 68908970 | *Per1* | CLOCK | 129S1 |
| 68908750 | 3 | chr11 | 68908511 | 68908970 | *Per1* | CLOCK | 129S1 |
| 68908813 | 3 | chr11 | 68908511 | 68908970 | *Per1* | CLOCK | 129S1 |
| 68908960 | 3 | chr11 | 68908511 | 68908970 | *Per1* | CLOCK | 129S1 |
| 68908968 | 3 | chr11 | 68908511 | 68908970 | *Per1* | CLOCK | 129S1 |
| 68908310 | 3 | chr11 | 68908231 | 68908501 | *Per1* | CLOCK | 129S1 |
| 98637109 | 3 | chr11 | 98636441 | 98637151 | *Nr1d1* | CLOCK | 129S1 |
| 98644565 | 3 | chr11 | 98644521 | 98645140 | *Nr1d1* | CLOCK | 129S1 |
| 98644843 | 3 | chr11 | 98644521 | 98645140 | *Nr1d1* | CLOCK | 129S1 |
| 98644918 | 3 | chr11 | 98644521 | 98645140 | *Nr1d1* | CLOCK | 129S1 |
| 98644508 | 3 | chr11 | 98644421 | 98644671 | *Nr1d1* | CLOCK | 129S1 |
| 98644565 | 3 | chr11 | 98644421 | 98644671 | *Nr1d1* | CLOCK | 129S1 |
| 93355989 | 3 | chr1 | 93355841 | 93356161 | *Per2* | CLOCK | 129S1 |
| 93356260 | 3 | chr1 | 93356221 | 93356440 | *Per2* | CLOCK | 129S1 |
| 93356397 | 3 | chr1 | 93356221 | 93356440 | *Per2* | CLOCK | 129S1 |
| 52960092 | 3 | chr7 | 52960041 | 52960800 | *Dbp* | CLOCK | 129S1 |
| 52960117 | 3 | chr7 | 52960041 | 52960800 | *Dbp* | CLOCK | 129S1 |
| 52960169 | 3 | chr7 | 52960041 | 52960800 | *Dbp* | CLOCK | 129S1 |
| 52960311 | 3 | chr7 | 52960041 | 52960800 | *Dbp* | CLOCK | 129S1 |
| 52960312 | 3 | chr7 | 52960041 | 52960800 | *Dbp* | CLOCK | 129S1 |
| 52960341 | 3 | chr7 | 52960041 | 52960800 | *Dbp* | CLOCK | 129S1 |
| 52960671 | 3 | chr7 | 52960041 | 52960800 | *Dbp* | CLOCK | 129S1 |
| 52961354 | 3 | chr7 | 52961331 | 52961720 | *Dbp* | CLOCK | 129S1 |
| 52961679 | 3 | chr7 | 52961331 | 52961720 | *Dbp* | CLOCK | 129S1 |
| 52963101 | 3 | chr7 | 52962911 | 52963280 | *Dbp* | CLOCK | 129S1 |
| 52963127 | 3 | chr7 | 52962911 | 52963280 | *Dbp* | CLOCK | 129S1 |
| 52963230 | 3 | chr7 | 52962911 | 52963280 | *Dbp* | CLOCK | 129S1 |
| 52963234 | 3 | chr7 | 52962911 | 52963280 | *Dbp* | CLOCK | 129S1 |
| 52960092 | 3 | chr7 | 52960071 | 52960211 | *Dbp* | CLOCK | 129S1 |
| 52960117 | 3 | chr7 | 52960071 | 52960211 | *Dbp* | CLOCK | 129S1 |
| 52960169 | 3 | chr7 | 52960071 | 52960211 | *Dbp* | CLOCK | 129S1 |
| 52960671 | 3 | chr7 | 52960581 | 52960840 | *Dbp* | CLOCK | 129S1 |
| 46716220 | 3 | chr19 | 46716061 | 46716420 | *Cyp17a1* | CLOCK | 129S1 |
| 51943736 | 3 | chr5 | 51943691 | 51944020 | *Ppargc1a* | CLOCK | 129S1 |
| 51943955 | 3 | chr5 | 51943691 | 51944020 | *Ppargc1a* | CLOCK | 129S1 |
| 68908614 | 3 | chr11 | 68908501 | 68908880 | *Per1* | PER1 | 129S1 |
| 68908620 | 3 | chr11 | 68908501 | 68908880 | *Per1* | PER1 | 129S1 |
| 68908750 | 3 | chr11 | 68908501 | 68908880 | *Per1* | PER1 | 129S1 |
| 68908813 | 3 | chr11 | 68908501 | 68908880 | *Per1* | PER1 | 129S1 |
| 68908310 | 3 | chr11 | 68908231 | 68908471 | *Per1* | PER1 | 129S1 |
| 98637109 | 3 | chr11 | 98636511 | 98637140 | *Nr1d1* | PER1 | 129S1 |
| 98644508 | 3 | chr11 | 98644481 | 98645140 | *Nr1d1* | PER1 | 129S1 |
| 98644565 | 3 | chr11 | 98644481 | 98645140 | *Nr1d1* | PER1 | 129S1 |
| 98644843 | 3 | chr11 | 98644481 | 98645140 | *Nr1d1* | PER1 | 129S1 |
| 98644918 | 3 | chr11 | 98644481 | 98645140 | *Nr1d1* | PER1 | 129S1 |
| 93355989 | 3 | chr1 | 93355871 | 93356151 | *Per2* | PER1 | 129S1 |
| 52960092 | 3 | chr7 | 52960021 | 52960541 | *Dbp* | PER1 | 129S1 |
| 52960117 | 3 | chr7 | 52960021 | 52960541 | *Dbp* | PER1 | 129S1 |
| 52960169 | 3 | chr7 | 52960021 | 52960541 | *Dbp* | PER1 | 129S1 |
| 52960311 | 3 | chr7 | 52960021 | 52960541 | *Dbp* | PER1 | 129S1 |
| 52960312 | 3 | chr7 | 52960021 | 52960541 | *Dbp* | PER1 | 129S1 |
| 52960341 | 3 | chr7 | 52960021 | 52960541 | *Dbp* | PER1 | 129S1 |
| 52963101 | 3 | chr7 | 52963041 | 52963300 | *Dbp* | PER1 | 129S1 |
| 52963127 | 3 | chr7 | 52963041 | 52963300 | *Dbp* | PER1 | 129S1 |
| 52963230 | 3 | chr7 | 52963041 | 52963300 | *Dbp* | PER1 | 129S1 |
| 52963234 | 3 | chr7 | 52963041 | 52963300 | *Dbp* | PER1 | 129S1 |
| 52961354 | 3 | chr7 | 52961341 | 52961610 | *Dbp* | PER1 | 129S1 |
| 46716220 | 3 | chr19 | 46716091 | 46716440 | *Cyp17a1* | PER1 | 129S1 |
| 46720943 | 3 | chr19 | 46720841 | 46721100 | *Cyp17a1* | PER1 | 129S1 |
| 68908614 | 3 | chr11 | 68908521 | 68908990 | *Per1* | PER2 | 129S1 |
| 68908620 | 3 | chr11 | 68908521 | 68908990 | *Per1* | PER2 | 129S1 |
| 68908750 | 3 | chr11 | 68908521 | 68908990 | *Per1* | PER2 | 129S1 |
| 68908813 | 3 | chr11 | 68908521 | 68908990 | *Per1* | PER2 | 129S1 |
| 68908960 | 3 | chr11 | 68908521 | 68908990 | *Per1* | PER2 | 129S1 |
| 68908968 | 3 | chr11 | 68908521 | 68908990 | *Per1* | PER2 | 129S1 |
| 68908310 | 3 | chr11 | 68908161 | 68908531 | *Per1* | PER2 | 129S1 |
| 98637109 | 3 | chr11 | 98636471 | 98637170 | *Nr1d1* | PER2 | 129S1 |
| 98644843 | 3 | chr11 | 98644661 | 98645150 | *Nr1d1* | PER2 | 129S1 |
| 98644918 | 3 | chr11 | 98644661 | 98645150 | *Nr1d1* | PER2 | 129S1 |
| 98634168 | 3 | chr11 | 98634141 | 98634530 | *Nr1d1* | PER2 | 129S1 |
| 98644508 | 3 | chr11 | 98644501 | 98644651 | *Nr1d1* | PER2 | 129S1 |
| 98644565 | 3 | chr11 | 98644501 | 98644651 | *Nr1d1* | PER2 | 129S1 |
| 93355989 | 3 | chr1 | 93355831 | 93356161 | *Per2* | PER2 | 129S1 |
| 93356260 | 3 | chr1 | 93356201 | 93356470 | *Per2* | PER2 | 129S1 |
| 93356397 | 3 | chr1 | 93356201 | 93356470 | *Per2* | PER2 | 129S1 |
| 93333537 | 3 | chr1 | 93333251 | 93333610 | *Per2* | PER2 | 129S1 |
| 150419182 | 3 | chr4 | 150418961 | 150419360 | *Per3* | PER2 | 129S1 |
| 150419349 | 3 | chr4 | 150418961 | 150419360 | *Per3* | PER2 | 129S1 |
| 150417652 | 3 | chr4 | 150417461 | 150417711 | *Per3* | PER2 | 129S1 |
| 52960092 | 3 | chr7 | 52960051 | 52960661 | *Dbp* | PER2 | 129S1 |
| 52960117 | 3 | chr7 | 52960051 | 52960661 | *Dbp* | PER2 | 129S1 |
| 52960169 | 3 | chr7 | 52960051 | 52960661 | *Dbp* | PER2 | 129S1 |
| 52960311 | 3 | chr7 | 52960051 | 52960661 | *Dbp* | PER2 | 129S1 |
| 52960312 | 3 | chr7 | 52960051 | 52960661 | *Dbp* | PER2 | 129S1 |
| 52960341 | 3 | chr7 | 52960051 | 52960661 | *Dbp* | PER2 | 129S1 |
| 52963101 | 3 | chr7 | 52962941 | 52963510 | *Dbp* | PER2 | 129S1 |
| 52963127 | 3 | chr7 | 52962941 | 52963510 | *Dbp* | PER2 | 129S1 |
| 52963230 | 3 | chr7 | 52962941 | 52963510 | *Dbp* | PER2 | 129S1 |
| 52963234 | 3 | chr7 | 52962941 | 52963510 | *Dbp* | PER2 | 129S1 |
| 52963367 | 3 | chr7 | 52962941 | 52963510 | *Dbp* | PER2 | 129S1 |
| 52963415 | 3 | chr7 | 52962941 | 52963510 | *Dbp* | PER2 | 129S1 |
| 52963419 | 3 | chr7 | 52962941 | 52963510 | *Dbp* | PER2 | 129S1 |
| 52961354 | 3 | chr7 | 52961311 | 52961650 | *Dbp* | PER2 | 129S1 |
| 46720828 | 3 | chr19 | 46720731 | 46721050 | *Cyp17a1* | PER2 | 129S1 |
| 46720829 | 3 | chr19 | 46720731 | 46721050 | *Cyp17a1* | PER2 | 129S1 |
| 46720943 | 3 | chr19 | 46720731 | 46721050 | *Cyp17a1* | PER2 | 129S1 |
| 46716220 | 3 | chr19 | 46716131 | 46716430 | *Cyp17a1* | PER2 | 129S1 |
| 120350590 | 3 | chr7 | 120350461 | 120351050 | *Arntl* | CRY1 | 129S1 |
| 52960092 | 3 | chr7 | 52959971 | 52960760 | *Dbp* | CRY1 | 129S1 |
| 52960117 | 3 | chr7 | 52959971 | 52960760 | *Dbp* | CRY1 | 129S1 |
| 52960169 | 3 | chr7 | 52959971 | 52960760 | *Dbp* | CRY1 | 129S1 |
| 52960311 | 3 | chr7 | 52959971 | 52960760 | *Dbp* | CRY1 | 129S1 |
| 52960312 | 3 | chr7 | 52959971 | 52960760 | *Dbp* | CRY1 | 129S1 |
| 52960341 | 3 | chr7 | 52959971 | 52960760 | *Dbp* | CRY1 | 129S1 |
| 52960671 | 3 | chr7 | 52959971 | 52960760 | *Dbp* | CRY1 | 129S1 |
| 52963101 | 3 | chr7 | 52962941 | 52963361 | *Dbp* | CRY1 | 129S1 |
| 52963127 | 3 | chr7 | 52962941 | 52963361 | *Dbp* | CRY1 | 129S1 |
| 52963230 | 3 | chr7 | 52962941 | 52963361 | *Dbp* | CRY1 | 129S1 |
| 52963234 | 3 | chr7 | 52962941 | 52963361 | *Dbp* | CRY1 | 129S1 |
| 52961354 | 3 | chr7 | 52961341 | 52961720 | *Dbp* | CRY1 | 129S1 |
| 52961679 | 3 | chr7 | 52961341 | 52961720 | *Dbp* | CRY1 | 129S1 |
| 52963415 | 3 | chr7 | 52963371 | 52963971 | *Dbp* | CRY1 | 129S1 |
| 52963419 | 3 | chr7 | 52963371 | 52963971 | *Dbp* | CRY1 | 129S1 |
| 52963542 | 3 | chr7 | 52963371 | 52963971 | *Dbp* | CRY1 | 129S1 |
| 52960671 | 3 | chr7 | 52960521 | 52960701 | *Dbp* | CRY1 | 129S1 |
| 52959755 | 3 | chr7 | 52959651 | 52959951 | *Dbp* | CRY1 | 129S1 |
| 52959760 | 3 | chr7 | 52959651 | 52959951 | *Dbp* | CRY1 | 129S1 |
| 52959795 | 3 | chr7 | 52959651 | 52959951 | *Dbp* | CRY1 | 129S1 |
| 52959486 | 3 | chr7 | 52959301 | 52959631 | *Dbp* | CRY1 | 129S1 |
| 52959594 | 3 | chr7 | 52959301 | 52959631 | *Dbp* | CRY1 | 129S1 |
| 52959619 | 3 | chr7 | 52959301 | 52959631 | *Dbp* | CRY1 | 129S1 |
| 68908310 | 3 | chr11 | 68907811 | 68908521 | *Per1* | CRY1 | 129S1 |
| 68908614 | 3 | chr11 | 68908531 | 68908941 | *Per1* | CRY1 | 129S1 |
| 68908620 | 3 | chr11 | 68908531 | 68908941 | *Per1* | CRY1 | 129S1 |
| 68908750 | 3 | chr11 | 68908531 | 68908941 | *Per1* | CRY1 | 129S1 |
| 68908813 | 3 | chr11 | 68908531 | 68908941 | *Per1* | CRY1 | 129S1 |
| 98637109 | 3 | chr11 | 98636401 | 98637420 | *Nr1d1* | CRY1 | 129S1 |
| 98644565 | 3 | chr11 | 98644521 | 98645050 | *Nr1d1* | CRY1 | 129S1 |
| 98644843 | 3 | chr11 | 98644521 | 98645050 | *Nr1d1* | CRY1 | 129S1 |
| 98644918 | 3 | chr11 | 98644521 | 98645050 | *Nr1d1* | CRY1 | 129S1 |
| 98634168 | 3 | chr11 | 98634161 | 98634611 | *Nr1d1* | CRY1 | 129S1 |
| 98644508 | 3 | chr11 | 98644481 | 98644651 | *Nr1d1* | CRY1 | 129S1 |
| 98644565 | 3 | chr11 | 98644481 | 98644651 | *Nr1d1* | CRY1 | 129S1 |
| 98636167 | 3 | chr11 | 98635971 | 98636171 | *Nr1d1* | CRY1 | 129S1 |
| 93355989 | 3 | chr1 | 93355741 | 93356191 | *Per2* | CRY1 | 129S1 |
| 93356260 | 3 | chr1 | 93356201 | 93356490 | *Per2* | CRY1 | 129S1 |
| 93356397 | 3 | chr1 | 93356201 | 93356490 | *Per2* | CRY1 | 129S1 |
| 93353564 | 3 | chr1 | 93353041 | 93353620 | *Per2* | CRY1 | 129S1 |
| 93352816 | 3 | chr1 | 93352511 | 93352900 | *Per2* | CRY1 | 129S1 |
| 93337174 | 3 | chr1 | 93336811 | 93337230 | *Per2* | CRY1 | 129S1 |
| 93337211 | 3 | chr1 | 93336811 | 93337230 | *Per2* | CRY1 | 129S1 |
| 93333537 | 3 | chr1 | 93333191 | 93333720 | *Per2* | CRY1 | 129S1 |
| 150418904 | 3 | chr4 | 150418811 | 150419271 | *Per3* | CRY1 | 129S1 |
| 150419182 | 3 | chr4 | 150418811 | 150419271 | *Per3* | CRY1 | 129S1 |
| 150417652 | 3 | chr4 | 150417501 | 150417850 | *Per3* | CRY1 | 129S1 |
| 150417822 | 3 | chr4 | 150417501 | 150417850 | *Per3* | CRY1 | 129S1 |
| 27490820 | 3 | chr2 | 27490731 | 27491100 | *Rxra* | CRY1 | 129S1 |
| 27490957 | 3 | chr2 | 27490731 | 27491100 | *Rxra* | CRY1 | 129S1 |
| 27490970 | 3 | chr2 | 27490731 | 27491100 | *Rxra* | CRY1 | 129S1 |
| 27490972 | 3 | chr2 | 27490731 | 27491100 | *Rxra* | CRY1 | 129S1 |
| 27490974 | 3 | chr2 | 27490731 | 27491100 | *Rxra* | CRY1 | 129S1 |
| 18068970 | 3 | chr3 | 18068881 | 18069180 | *Cyp7b1* | CRY1 | 129S1 |
| 18151353 | 3 | chr3 | 18151181 | 18151490 | *Cyp7b1* | CRY1 | 129S1 |
| 46716220 | 3 | chr19 | 46716091 | 46716450 | *Cyp17a1* | CRY1 | 129S1 |
| 46720828 | 3 | chr19 | 46720771 | 46721040 | *Cyp17a1* | CRY1 | 129S1 |
| 46720829 | 3 | chr19 | 46720771 | 46721040 | *Cyp17a1* | CRY1 | 129S1 |
| 46720943 | 3 | chr19 | 46720771 | 46721040 | *Cyp17a1* | CRY1 | 129S1 |
| 68908614 | 3 | chr11 | 68908521 | 68908920 | *Per1* | CRY2 | 129S1 |
| 68908620 | 3 | chr11 | 68908521 | 68908920 | *Per1* | CRY2 | 129S1 |
| 68908750 | 3 | chr11 | 68908521 | 68908920 | *Per1* | CRY2 | 129S1 |
| 68908813 | 3 | chr11 | 68908521 | 68908920 | *Per1* | CRY2 | 129S1 |
| 68908310 | 3 | chr11 | 68908221 | 68908511 | *Per1* | CRY2 | 129S1 |
| 98637109 | 3 | chr11 | 98636431 | 98637110 | *Nr1d1* | CRY2 | 129S1 |
| 98644843 | 3 | chr11 | 98644651 | 98645060 | *Nr1d1* | CRY2 | 129S1 |
| 98644918 | 3 | chr11 | 98644651 | 98645060 | *Nr1d1* | CRY2 | 129S1 |
| 98634168 | 3 | chr11 | 98634151 | 98634541 | *Nr1d1* | CRY2 | 129S1 |
| 98637109 | 3 | chr11 | 98636911 | 98637150 | *Nr1d1* | CRY2 | 129S1 |
| 98644508 | 3 | chr11 | 98644471 | 98644661 | *Nr1d1* | CRY2 | 129S1 |
| 98644565 | 3 | chr11 | 98644471 | 98644661 | *Nr1d1* | CRY2 | 129S1 |
| 93355989 | 3 | chr1 | 93355861 | 93356171 | *Per2* | CRY2 | 129S1 |
| 93356260 | 3 | chr1 | 93356181 | 93356411 | *Per2* | CRY2 | 129S1 |
| 93356397 | 3 | chr1 | 93356181 | 93356411 | *Per2* | CRY2 | 129S1 |
| 150419182 | 3 | chr4 | 150418961 | 150419191 | *Per3* | CRY2 | 129S1 |
| 27482311 | 3 | chr2 | 27482231 | 27482470 | *Rxra* | CRY2 | 129S1 |
| 27482313 | 3 | chr2 | 27482231 | 27482470 | *Rxra* | CRY2 | 129S1 |
| 27482370 | 3 | chr2 | 27482231 | 27482470 | *Rxra* | CRY2 | 129S1 |
| 52960092 | 3 | chr7 | 52959961 | 52960611 | *Dbp* | CRY2 | 129S1 |
| 52960117 | 3 | chr7 | 52959961 | 52960611 | *Dbp* | CRY2 | 129S1 |
| 52960169 | 3 | chr7 | 52959961 | 52960611 | *Dbp* | CRY2 | 129S1 |
| 52960311 | 3 | chr7 | 52959961 | 52960611 | *Dbp* | CRY2 | 129S1 |
| 52960312 | 3 | chr7 | 52959961 | 52960611 | *Dbp* | CRY2 | 129S1 |
| 52960341 | 3 | chr7 | 52959961 | 52960611 | *Dbp* | CRY2 | 129S1 |
| 52963101 | 3 | chr7 | 52962841 | 52963420 | *Dbp* | CRY2 | 129S1 |
| 52963127 | 3 | chr7 | 52962841 | 52963420 | *Dbp* | CRY2 | 129S1 |
| 52963230 | 3 | chr7 | 52962841 | 52963420 | *Dbp* | CRY2 | 129S1 |
| 52963234 | 3 | chr7 | 52962841 | 52963420 | *Dbp* | CRY2 | 129S1 |
| 52963367 | 3 | chr7 | 52962841 | 52963420 | *Dbp* | CRY2 | 129S1 |
| 52963415 | 3 | chr7 | 52962841 | 52963420 | *Dbp* | CRY2 | 129S1 |
| 52963419 | 3 | chr7 | 52962841 | 52963420 | *Dbp* | CRY2 | 129S1 |
| 52960671 | 3 | chr7 | 52960481 | 52960721 | *Dbp* | CRY2 | 129S1 |
| 52961354 | 3 | chr7 | 52961281 | 52961581 | *Dbp* | CRY2 | 129S1 |
| 46720828 | 3 | chr19 | 46720721 | 46721040 | *Cyp17a1* | CRY2 | 129S1 |
| 46720829 | 3 | chr19 | 46720721 | 46721040 | *Cyp17a1* | CRY2 | 129S1 |
| 46720943 | 3 | chr19 | 46720721 | 46721040 | *Cyp17a1* | CRY2 | 129S1 |
| 46716220 | 3 | chr19 | 46716111 | 46716590 | *Cyp17a1* | CRY2 | 129S1 |
| 68908614 | 3 | chr11 | 68908481 | 68908800 | *Per1* | NPAS2 | 129S1 |
| 68908620 | 3 | chr11 | 68908481 | 68908800 | *Per1* | NPAS2 | 129S1 |
| 68908750 | 3 | chr11 | 68908481 | 68908800 | *Per1* | NPAS2 | 129S1 |
| 68908310 | 3 | chr11 | 68908221 | 68908471 | *Per1* | NPAS2 | 129S1 |
| 98637109 | 3 | chr11 | 98636601 | 98637151 | *Nr1d1* | NPAS2 | 129S1 |
| 98644843 | 3 | chr11 | 98644621 | 98645190 | *Nr1d1* | NPAS2 | 129S1 |
| 98644918 | 3 | chr11 | 98644621 | 98645190 | *Nr1d1* | NPAS2 | 129S1 |
| 98637109 | 3 | chr11 | 98636841 | 98637150 | *Nr1d1* | NPAS2 | 129S1 |
| 98644918 | 3 | chr11 | 98644871 | 98645040 | *Nr1d1* | NPAS2 | 129S1 |
| 98644508 | 3 | chr11 | 98644481 | 98644871 | *Nr1d1* | NPAS2 | 129S1 |
| 98644565 | 3 | chr11 | 98644481 | 98644871 | *Nr1d1* | NPAS2 | 129S1 |
| 98644843 | 3 | chr11 | 98644481 | 98644871 | *Nr1d1* | NPAS2 | 129S1 |
| 93355989 | 3 | chr1 | 93355851 | 93356161 | *Per2* | NPAS2 | 129S1 |
| 93356260 | 3 | chr1 | 93356201 | 93356420 | *Per2* | NPAS2 | 129S1 |
| 93356397 | 3 | chr1 | 93356201 | 93356420 | *Per2* | NPAS2 | 129S1 |
| 52960092 | 3 | chr7 | 52960041 | 52960561 | *Dbp* | NPAS2 | 129S1 |
| 52960117 | 3 | chr7 | 52960041 | 52960561 | *Dbp* | NPAS2 | 129S1 |
| 52960169 | 3 | chr7 | 52960041 | 52960561 | *Dbp* | NPAS2 | 129S1 |
| 52960311 | 3 | chr7 | 52960041 | 52960561 | *Dbp* | NPAS2 | 129S1 |
| 52960312 | 3 | chr7 | 52960041 | 52960561 | *Dbp* | NPAS2 | 129S1 |
| 52960341 | 3 | chr7 | 52960041 | 52960561 | *Dbp* | NPAS2 | 129S1 |
| 52961354 | 3 | chr7 | 52961241 | 52961601 | *Dbp* | NPAS2 | 129S1 |
| 52963101 | 3 | chr7 | 52963061 | 52963370 | *Dbp* | NPAS2 | 129S1 |
| 52963127 | 3 | chr7 | 52963061 | 52963370 | *Dbp* | NPAS2 | 129S1 |
| 52963230 | 3 | chr7 | 52963061 | 52963370 | *Dbp* | NPAS2 | 129S1 |
| 52963234 | 3 | chr7 | 52963061 | 52963370 | *Dbp* | NPAS2 | 129S1 |
| 52963367 | 3 | chr7 | 52963061 | 52963370 | *Dbp* | NPAS2 | 129S1 |
| 52963542 | 3 | chr7 | 52963541 | 52963900 | *Dbp* | NPAS2 | 129S1 |
| 68908310 | 2 | chr11 | 68908211 | 68908501 | *Per1* | BMAL1 | 129S6 |
| 68908614 | 2 | chr11 | 68908511 | 68908830 | *Per1* | BMAL1 | 129S6 |
| 68908620 | 2 | chr11 | 68908511 | 68908830 | *Per1* | BMAL1 | 129S6 |
| 68908750 | 2 | chr11 | 68908511 | 68908830 | *Per1* | BMAL1 | 129S6 |
| 68908813 | 2 | chr11 | 68908511 | 68908830 | *Per1* | BMAL1 | 129S6 |
| 98644843 | 2 | chr11 | 98644571 | 98645021 | *Nr1d1* | BMAL1 | 129S6 |
| 98644918 | 2 | chr11 | 98644571 | 98645021 | *Nr1d1* | BMAL1 | 129S6 |
| 98637109 | 2 | chr11 | 98636841 | 98637181 | *Nr1d1* | BMAL1 | 129S6 |
| 98634168 | 2 | chr11 | 98634141 | 98634561 | *Nr1d1* | BMAL1 | 129S6 |
| 98643374 | 2 | chr11 | 98643181 | 98643480 | *Nr1d1* | BMAL1 | 129S6 |
| 98643452 | 2 | chr11 | 98643181 | 98643480 | *Nr1d1* | BMAL1 | 129S6 |
| 98643456 | 1 | chr11 | 98643181 | 98643480 | *Nr1d1* | BMAL1 | 129S6 |
| 93355989 | 2 | chr1 | 93355801 | 93356370 | *Per2* | BMAL1 | 129S6 |
| 93356260 | 2 | chr1 | 93355801 | 93356370 | *Per2* | BMAL1 | 129S6 |
| 93356260 | 2 | chr1 | 93356181 | 93356600 | *Per2* | BMAL1 | 129S6 |
| 93356397 | 2 | chr1 | 93356181 | 93356600 | *Per2* | BMAL1 | 129S6 |
| 150419182 | 2 | chr4 | 150418941 | 150419210 | *Per3* | BMAL1 | 129S6 |
| 150418904 | 2 | chr4 | 150418731 | 150418911 | *Per3* | BMAL1 | 129S6 |
| 27480259 | 2 | chr2 | 27480231 | 27480580 | *Rxra* | BMAL1 | 129S6 |
| 52960092 | 2 | chr7 | 52960041 | 52960760 | *Dbp* | BMAL1 | 129S6 |
| 52960117 | 2 | chr7 | 52960041 | 52960760 | *Dbp* | BMAL1 | 129S6 |
| 52960169 | 2 | chr7 | 52960041 | 52960760 | *Dbp* | BMAL1 | 129S6 |
| 52960311 | 2 | chr7 | 52960041 | 52960760 | *Dbp* | BMAL1 | 129S6 |
| 52960312 | 2 | chr7 | 52960041 | 52960760 | *Dbp* | BMAL1 | 129S6 |
| 52960341 | 2 | chr7 | 52960041 | 52960760 | *Dbp* | BMAL1 | 129S6 |
| 52960671 | 2 | chr7 | 52960041 | 52960760 | *Dbp* | BMAL1 | 129S6 |
| 52962826 | 2 | chr7 | 52962811 | 52963430 | *Dbp* | BMAL1 | 129S6 |
| 52963101 | 2 | chr7 | 52962811 | 52963430 | *Dbp* | BMAL1 | 129S6 |
| 52963127 | 2 | chr7 | 52962811 | 52963430 | *Dbp* | BMAL1 | 129S6 |
| 52963230 | 2 | chr7 | 52962811 | 52963430 | *Dbp* | BMAL1 | 129S6 |
| 52963234 | 2 | chr7 | 52962811 | 52963430 | *Dbp* | BMAL1 | 129S6 |
| 52963367 | 2 | chr7 | 52962811 | 52963430 | *Dbp* | BMAL1 | 129S6 |
| 52963415 | 2 | chr7 | 52962811 | 52963430 | *Dbp* | BMAL1 | 129S6 |
| 52963419 | 2 | chr7 | 52962811 | 52963430 | *Dbp* | BMAL1 | 129S6 |
| 52961354 | 2 | chr7 | 52961201 | 52961730 | *Dbp* | BMAL1 | 129S6 |
| 52961679 | 2 | chr7 | 52961201 | 52961730 | *Dbp* | BMAL1 | 129S6 |
| 52961720 | 2 | chr7 | 52961201 | 52961730 | *Dbp* | BMAL1 | 129S6 |
| 46716220 | 2 | chr19 | 46716101 | 46716281 | *Cyp17a1* | BMAL1 | 129S6 |
| 51943955 | 2 | chr5 | 51943791 | 51944090 | *Ppargc1a* | BMAL1 | 129S6 |
| 68908614 | 2 | chr11 | 68908511 | 68908970 | *Per1* | CLOCK | 129S6 |
| 68908620 | 2 | chr11 | 68908511 | 68908970 | *Per1* | CLOCK | 129S6 |
| 68908750 | 2 | chr11 | 68908511 | 68908970 | *Per1* | CLOCK | 129S6 |
| 68908813 | 2 | chr11 | 68908511 | 68908970 | *Per1* | CLOCK | 129S6 |
| 68908960 | 2 | chr11 | 68908511 | 68908970 | *Per1* | CLOCK | 129S6 |
| 68908968 | 2 | chr11 | 68908511 | 68908970 | *Per1* | CLOCK | 129S6 |
| 68908310 | 2 | chr11 | 68908231 | 68908501 | *Per1* | CLOCK | 129S6 |
| 98637109 | 2 | chr11 | 98636441 | 98637151 | *Nr1d1* | CLOCK | 129S6 |
| 98644565 | 2 | chr11 | 98644521 | 98645140 | *Nr1d1* | CLOCK | 129S6 |
| 98644843 | 2 | chr11 | 98644521 | 98645140 | *Nr1d1* | CLOCK | 129S6 |
| 98644918 | 2 | chr11 | 98644521 | 98645140 | *Nr1d1* | CLOCK | 129S6 |
| 98644508 | 2 | chr11 | 98644421 | 98644671 | *Nr1d1* | CLOCK | 129S6 |
| 98644565 | 2 | chr11 | 98644421 | 98644671 | *Nr1d1* | CLOCK | 129S6 |
| 93355989 | 2 | chr1 | 93355841 | 93356161 | *Per2* | CLOCK | 129S6 |
| 93356260 | 2 | chr1 | 93356221 | 93356440 | *Per2* | CLOCK | 129S6 |
| 93356397 | 2 | chr1 | 93356221 | 93356440 | *Per2* | CLOCK | 129S6 |
| 52960092 | 2 | chr7 | 52960041 | 52960800 | *Dbp* | CLOCK | 129S6 |
| 52960117 | 2 | chr7 | 52960041 | 52960800 | *Dbp* | CLOCK | 129S6 |
| 52960169 | 2 | chr7 | 52960041 | 52960800 | *Dbp* | CLOCK | 129S6 |
| 52960311 | 2 | chr7 | 52960041 | 52960800 | *Dbp* | CLOCK | 129S6 |
| 52960312 | 2 | chr7 | 52960041 | 52960800 | *Dbp* | CLOCK | 129S6 |
| 52960341 | 2 | chr7 | 52960041 | 52960800 | *Dbp* | CLOCK | 129S6 |
| 52960671 | 2 | chr7 | 52960041 | 52960800 | *Dbp* | CLOCK | 129S6 |
| 52961354 | 2 | chr7 | 52961331 | 52961720 | *Dbp* | CLOCK | 129S6 |
| 52961679 | 2 | chr7 | 52961331 | 52961720 | *Dbp* | CLOCK | 129S6 |
| 52963101 | 2 | chr7 | 52962911 | 52963280 | *Dbp* | CLOCK | 129S6 |
| 52963127 | 2 | chr7 | 52962911 | 52963280 | *Dbp* | CLOCK | 129S6 |
| 52963230 | 2 | chr7 | 52962911 | 52963280 | *Dbp* | CLOCK | 129S6 |
| 52963234 | 2 | chr7 | 52962911 | 52963280 | *Dbp* | CLOCK | 129S6 |
| 52960092 | 2 | chr7 | 52960071 | 52960211 | *Dbp* | CLOCK | 129S6 |
| 52960117 | 2 | chr7 | 52960071 | 52960211 | *Dbp* | CLOCK | 129S6 |
| 52960169 | 2 | chr7 | 52960071 | 52960211 | *Dbp* | CLOCK | 129S6 |
| 52960671 | 2 | chr7 | 52960581 | 52960840 | *Dbp* | CLOCK | 129S6 |
| 46716220 | 2 | chr19 | 46716061 | 46716420 | *Cyp17a1* | CLOCK | 129S6 |
| 51943736 | 2 | chr5 | 51943691 | 51944020 | *Ppargc1a* | CLOCK | 129S6 |
| 51943955 | 2 | chr5 | 51943691 | 51944020 | *Ppargc1a* | CLOCK | 129S6 |
| 68908614 | 2 | chr11 | 68908501 | 68908880 | *Per1* | PER1 | 129S6 |
| 68908620 | 2 | chr11 | 68908501 | 68908880 | *Per1* | PER1 | 129S6 |
| 68908750 | 2 | chr11 | 68908501 | 68908880 | *Per1* | PER1 | 129S6 |
| 68908813 | 2 | chr11 | 68908501 | 68908880 | *Per1* | PER1 | 129S6 |
| 68908310 | 2 | chr11 | 68908231 | 68908471 | *Per1* | PER1 | 129S6 |
| 98637109 | 2 | chr11 | 98636511 | 98637140 | *Nr1d1* | PER1 | 129S6 |
| 98644508 | 2 | chr11 | 98644481 | 98645140 | *Nr1d1* | PER1 | 129S6 |
| 98644565 | 2 | chr11 | 98644481 | 98645140 | *Nr1d1* | PER1 | 129S6 |
| 98644843 | 2 | chr11 | 98644481 | 98645140 | *Nr1d1* | PER1 | 129S6 |
| 98644918 | 2 | chr11 | 98644481 | 98645140 | *Nr1d1* | PER1 | 129S6 |
| 93355989 | 2 | chr1 | 93355871 | 93356151 | *Per2* | PER1 | 129S6 |
| 52960092 | 2 | chr7 | 52960021 | 52960541 | *Dbp* | PER1 | 129S6 |
| 52960117 | 2 | chr7 | 52960021 | 52960541 | *Dbp* | PER1 | 129S6 |
| 52960169 | 2 | chr7 | 52960021 | 52960541 | *Dbp* | PER1 | 129S6 |
| 52960311 | 2 | chr7 | 52960021 | 52960541 | *Dbp* | PER1 | 129S6 |
| 52960312 | 2 | chr7 | 52960021 | 52960541 | *Dbp* | PER1 | 129S6 |
| 52960341 | 2 | chr7 | 52960021 | 52960541 | *Dbp* | PER1 | 129S6 |
| 52963101 | 2 | chr7 | 52963041 | 52963300 | *Dbp* | PER1 | 129S6 |
| 52963127 | 2 | chr7 | 52963041 | 52963300 | *Dbp* | PER1 | 129S6 |
| 52963230 | 2 | chr7 | 52963041 | 52963300 | *Dbp* | PER1 | 129S6 |
| 52963234 | 2 | chr7 | 52963041 | 52963300 | *Dbp* | PER1 | 129S6 |
| 52961354 | 2 | chr7 | 52961341 | 52961610 | *Dbp* | PER1 | 129S6 |
| 46716220 | 2 | chr19 | 46716091 | 46716440 | *Cyp17a1* | PER1 | 129S6 |
| 46720943 | 2 | chr19 | 46720841 | 46721100 | *Cyp17a1* | PER1 | 129S6 |
| 68908614 | 2 | chr11 | 68908521 | 68908990 | *Per1* | PER2 | 129S6 |
| 68908620 | 2 | chr11 | 68908521 | 68908990 | *Per1* | PER2 | 129S6 |
| 68908750 | 2 | chr11 | 68908521 | 68908990 | *Per1* | PER2 | 129S6 |
| 68908813 | 2 | chr11 | 68908521 | 68908990 | *Per1* | PER2 | 129S6 |
| 68908960 | 2 | chr11 | 68908521 | 68908990 | *Per1* | PER2 | 129S6 |
| 68908968 | 2 | chr11 | 68908521 | 68908990 | *Per1* | PER2 | 129S6 |
| 68908310 | 2 | chr11 | 68908161 | 68908531 | *Per1* | PER2 | 129S6 |
| 98637109 | 2 | chr11 | 98636471 | 98637170 | *Nr1d1* | PER2 | 129S6 |
| 98644843 | 2 | chr11 | 98644661 | 98645150 | *Nr1d1* | PER2 | 129S6 |
| 98644918 | 2 | chr11 | 98644661 | 98645150 | *Nr1d1* | PER2 | 129S6 |
| 98634168 | 2 | chr11 | 98634141 | 98634530 | *Nr1d1* | PER2 | 129S6 |
| 98644508 | 2 | chr11 | 98644501 | 98644651 | *Nr1d1* | PER2 | 129S6 |
| 98644565 | 2 | chr11 | 98644501 | 98644651 | *Nr1d1* | PER2 | 129S6 |
| 93355989 | 2 | chr1 | 93355831 | 93356161 | *Per2* | PER2 | 129S6 |
| 93356260 | 2 | chr1 | 93356201 | 93356470 | *Per2* | PER2 | 129S6 |
| 93356397 | 2 | chr1 | 93356201 | 93356470 | *Per2* | PER2 | 129S6 |
| 93333537 | 2 | chr1 | 93333251 | 93333610 | *Per2* | PER2 | 129S6 |
| 150419182 | 2 | chr4 | 150418961 | 150419360 | *Per3* | PER2 | 129S6 |
| 150419349 | 2 | chr4 | 150418961 | 150419360 | *Per3* | PER2 | 129S6 |
| 150417652 | 2 | chr4 | 150417461 | 150417711 | *Per3* | PER2 | 129S6 |
| 52960092 | 2 | chr7 | 52960051 | 52960661 | *Dbp* | PER2 | 129S6 |
| 52960117 | 2 | chr7 | 52960051 | 52960661 | *Dbp* | PER2 | 129S6 |
| 52960169 | 2 | chr7 | 52960051 | 52960661 | *Dbp* | PER2 | 129S6 |
| 52960311 | 2 | chr7 | 52960051 | 52960661 | *Dbp* | PER2 | 129S6 |
| 52960312 | 2 | chr7 | 52960051 | 52960661 | *Dbp* | PER2 | 129S6 |
| 52960341 | 2 | chr7 | 52960051 | 52960661 | *Dbp* | PER2 | 129S6 |
| 52963101 | 2 | chr7 | 52962941 | 52963510 | *Dbp* | PER2 | 129S6 |
| 52963127 | 2 | chr7 | 52962941 | 52963510 | *Dbp* | PER2 | 129S6 |
| 52963230 | 2 | chr7 | 52962941 | 52963510 | *Dbp* | PER2 | 129S6 |
| 52963234 | 2 | chr7 | 52962941 | 52963510 | *Dbp* | PER2 | 129S6 |
| 52963367 | 2 | chr7 | 52962941 | 52963510 | *Dbp* | PER2 | 129S6 |
| 52963415 | 2 | chr7 | 52962941 | 52963510 | *Dbp* | PER2 | 129S6 |
| 52963419 | 2 | chr7 | 52962941 | 52963510 | *Dbp* | PER2 | 129S6 |
| 52961354 | 2 | chr7 | 52961311 | 52961650 | *Dbp* | PER2 | 129S6 |
| 46720828 | 2 | chr19 | 46720731 | 46721050 | *Cyp17a1* | PER2 | 129S6 |
| 46720829 | 2 | chr19 | 46720731 | 46721050 | *Cyp17a1* | PER2 | 129S6 |
| 46720943 | 2 | chr19 | 46720731 | 46721050 | *Cyp17a1* | PER2 | 129S6 |
| 46716220 | 2 | chr19 | 46716131 | 46716430 | *Cyp17a1* | PER2 | 129S6 |
| 120350590 | 2 | chr7 | 120350461 | 120351050 | *Arntl* | CRY1 | 129S6 |
| 52960092 | 2 | chr7 | 52959971 | 52960760 | *Dbp* | CRY1 | 129S6 |
| 52960117 | 2 | chr7 | 52959971 | 52960760 | *Dbp* | CRY1 | 129S6 |
| 52960169 | 2 | chr7 | 52959971 | 52960760 | *Dbp* | CRY1 | 129S6 |
| 52960311 | 2 | chr7 | 52959971 | 52960760 | *Dbp* | CRY1 | 129S6 |
| 52960312 | 2 | chr7 | 52959971 | 52960760 | *Dbp* | CRY1 | 129S6 |
| 52960341 | 2 | chr7 | 52959971 | 52960760 | *Dbp* | CRY1 | 129S6 |
| 52960671 | 2 | chr7 | 52959971 | 52960760 | *Dbp* | CRY1 | 129S6 |
| 52963101 | 2 | chr7 | 52962941 | 52963361 | *Dbp* | CRY1 | 129S6 |
| 52963127 | 2 | chr7 | 52962941 | 52963361 | *Dbp* | CRY1 | 129S6 |
| 52963230 | 2 | chr7 | 52962941 | 52963361 | *Dbp* | CRY1 | 129S6 |
| 52963234 | 2 | chr7 | 52962941 | 52963361 | *Dbp* | CRY1 | 129S6 |
| 52961354 | 2 | chr7 | 52961341 | 52961720 | *Dbp* | CRY1 | 129S6 |
| 52961679 | 2 | chr7 | 52961341 | 52961720 | *Dbp* | CRY1 | 129S6 |
| 52963415 | 2 | chr7 | 52963371 | 52963971 | *Dbp* | CRY1 | 129S6 |
| 52963419 | 2 | chr7 | 52963371 | 52963971 | *Dbp* | CRY1 | 129S6 |
| 52963542 | 2 | chr7 | 52963371 | 52963971 | *Dbp* | CRY1 | 129S6 |
| 52960671 | 2 | chr7 | 52960521 | 52960701 | *Dbp* | CRY1 | 129S6 |
| 52959755 | 2 | chr7 | 52959651 | 52959951 | *Dbp* | CRY1 | 129S6 |
| 52959760 | 2 | chr7 | 52959651 | 52959951 | *Dbp* | CRY1 | 129S6 |
| 52959795 | 2 | chr7 | 52959651 | 52959951 | *Dbp* | CRY1 | 129S6 |
| 52959486 | 2 | chr7 | 52959301 | 52959631 | *Dbp* | CRY1 | 129S6 |
| 52959594 | 2 | chr7 | 52959301 | 52959631 | *Dbp* | CRY1 | 129S6 |
| 52959619 | 2 | chr7 | 52959301 | 52959631 | *Dbp* | CRY1 | 129S6 |
| 68908310 | 2 | chr11 | 68907811 | 68908521 | *Per1* | CRY1 | 129S6 |
| 68908614 | 2 | chr11 | 68908531 | 68908941 | *Per1* | CRY1 | 129S6 |
| 68908620 | 2 | chr11 | 68908531 | 68908941 | *Per1* | CRY1 | 129S6 |
| 68908750 | 2 | chr11 | 68908531 | 68908941 | *Per1* | CRY1 | 129S6 |
| 68908813 | 2 | chr11 | 68908531 | 68908941 | *Per1* | CRY1 | 129S6 |
| 98637109 | 2 | chr11 | 98636401 | 98637420 | *Nr1d1* | CRY1 | 129S6 |
| 98644565 | 2 | chr11 | 98644521 | 98645050 | *Nr1d1* | CRY1 | 129S6 |
| 98644843 | 2 | chr11 | 98644521 | 98645050 | *Nr1d1* | CRY1 | 129S6 |
| 98644918 | 2 | chr11 | 98644521 | 98645050 | *Nr1d1* | CRY1 | 129S6 |
| 98634168 | 2 | chr11 | 98634161 | 98634611 | *Nr1d1* | CRY1 | 129S6 |
| 98644508 | 2 | chr11 | 98644481 | 98644651 | *Nr1d1* | CRY1 | 129S6 |
| 98644565 | 2 | chr11 | 98644481 | 98644651 | *Nr1d1* | CRY1 | 129S6 |
| 98636167 | 2 | chr11 | 98635971 | 98636171 | *Nr1d1* | CRY1 | 129S6 |
| 93355989 | 2 | chr1 | 93355741 | 93356191 | *Per2* | CRY1 | 129S6 |
| 93356260 | 2 | chr1 | 93356201 | 93356490 | *Per2* | CRY1 | 129S6 |
| 93356397 | 2 | chr1 | 93356201 | 93356490 | *Per2* | CRY1 | 129S6 |
| 93353564 | 2 | chr1 | 93353041 | 93353620 | *Per2* | CRY1 | 129S6 |
| 93352816 | 2 | chr1 | 93352511 | 93352900 | *Per2* | CRY1 | 129S6 |
| 93337174 | 2 | chr1 | 93336811 | 93337230 | *Per2* | CRY1 | 129S6 |
| 93337211 | 2 | chr1 | 93336811 | 93337230 | *Per2* | CRY1 | 129S6 |
| 93333537 | 2 | chr1 | 93333191 | 93333720 | *Per2* | CRY1 | 129S6 |
| 150418904 | 2 | chr4 | 150418811 | 150419271 | *Per3* | CRY1 | 129S6 |
| 150419182 | 2 | chr4 | 150418811 | 150419271 | *Per3* | CRY1 | 129S6 |
| 150417652 | 2 | chr4 | 150417501 | 150417850 | *Per3* | CRY1 | 129S6 |
| 150417822 | 2 | chr4 | 150417501 | 150417850 | *Per3* | CRY1 | 129S6 |
| 27490820 | 2 | chr2 | 27490731 | 27491100 | *Rxra* | CRY1 | 129S6 |
| 27490957 | 2 | chr2 | 27490731 | 27491100 | *Rxra* | CRY1 | 129S6 |
| 27490970 | 2 | chr2 | 27490731 | 27491100 | *Rxra* | CRY1 | 129S6 |
| 27490972 | 2 | chr2 | 27490731 | 27491100 | *Rxra* | CRY1 | 129S6 |
| 27490974 | 2 | chr2 | 27490731 | 27491100 | *Rxra* | CRY1 | 129S6 |
| 18068970 | 2 | chr3 | 18068881 | 18069180 | *Cyp7b1* | CRY1 | 129S6 |
| 18151353 | 2 | chr3 | 18151181 | 18151490 | *Cyp7b1* | CRY1 | 129S6 |
| 46716220 | 2 | chr19 | 46716091 | 46716450 | *Cyp17a1* | CRY1 | 129S6 |
| 46720828 | 2 | chr19 | 46720771 | 46721040 | *Cyp17a1* | CRY1 | 129S6 |
| 46720829 | 2 | chr19 | 46720771 | 46721040 | *Cyp17a1* | CRY1 | 129S6 |
| 46720943 | 2 | chr19 | 46720771 | 46721040 | *Cyp17a1* | CRY1 | 129S6 |
| 68908614 | 2 | chr11 | 68908521 | 68908920 | *Per1* | CRY2 | 129S6 |
| 68908620 | 2 | chr11 | 68908521 | 68908920 | *Per1* | CRY2 | 129S6 |
| 68908750 | 2 | chr11 | 68908521 | 68908920 | *Per1* | CRY2 | 129S6 |
| 68908813 | 2 | chr11 | 68908521 | 68908920 | *Per1* | CRY2 | 129S6 |
| 68908310 | 2 | chr11 | 68908221 | 68908511 | *Per1* | CRY2 | 129S6 |
| 98637109 | 2 | chr11 | 98636431 | 98637110 | *Nr1d1* | CRY2 | 129S6 |
| 98644843 | 2 | chr11 | 98644651 | 98645060 | *Nr1d1* | CRY2 | 129S6 |
| 98644918 | 2 | chr11 | 98644651 | 98645060 | *Nr1d1* | CRY2 | 129S6 |
| 98634168 | 2 | chr11 | 98634151 | 98634541 | *Nr1d1* | CRY2 | 129S6 |
| 98637109 | 2 | chr11 | 98636911 | 98637150 | *Nr1d1* | CRY2 | 129S6 |
| 98644508 | 2 | chr11 | 98644471 | 98644661 | *Nr1d1* | CRY2 | 129S6 |
| 98644565 | 2 | chr11 | 98644471 | 98644661 | *Nr1d1* | CRY2 | 129S6 |
| 93355989 | 2 | chr1 | 93355861 | 93356171 | *Per2* | CRY2 | 129S6 |
| 93356260 | 2 | chr1 | 93356181 | 93356411 | *Per2* | CRY2 | 129S6 |
| 93356397 | 2 | chr1 | 93356181 | 93356411 | *Per2* | CRY2 | 129S6 |
| 150419182 | 2 | chr4 | 150418961 | 150419191 | *Per3* | CRY2 | 129S6 |
| 27482311 | 2 | chr2 | 27482231 | 27482470 | *Rxra* | CRY2 | 129S6 |
| 27482313 | 2 | chr2 | 27482231 | 27482470 | *Rxra* | CRY2 | 129S6 |
| 27482370 | 2 | chr2 | 27482231 | 27482470 | *Rxra* | CRY2 | 129S6 |
| 52960092 | 2 | chr7 | 52959961 | 52960611 | *Dbp* | CRY2 | 129S6 |
| 52960117 | 2 | chr7 | 52959961 | 52960611 | *Dbp* | CRY2 | 129S6 |
| 52960169 | 2 | chr7 | 52959961 | 52960611 | *Dbp* | CRY2 | 129S6 |
| 52960311 | 2 | chr7 | 52959961 | 52960611 | *Dbp* | CRY2 | 129S6 |
| 52960312 | 2 | chr7 | 52959961 | 52960611 | *Dbp* | CRY2 | 129S6 |
| 52960341 | 2 | chr7 | 52959961 | 52960611 | *Dbp* | CRY2 | 129S6 |
| 52963101 | 2 | chr7 | 52962841 | 52963420 | *Dbp* | CRY2 | 129S6 |
| 52963127 | 2 | chr7 | 52962841 | 52963420 | *Dbp* | CRY2 | 129S6 |
| 52963230 | 2 | chr7 | 52962841 | 52963420 | *Dbp* | CRY2 | 129S6 |
| 52963234 | 2 | chr7 | 52962841 | 52963420 | *Dbp* | CRY2 | 129S6 |
| 52963367 | 2 | chr7 | 52962841 | 52963420 | *Dbp* | CRY2 | 129S6 |
| 52963415 | 2 | chr7 | 52962841 | 52963420 | *Dbp* | CRY2 | 129S6 |
| 52963419 | 2 | chr7 | 52962841 | 52963420 | *Dbp* | CRY2 | 129S6 |
| 52960671 | 2 | chr7 | 52960481 | 52960721 | *Dbp* | CRY2 | 129S6 |
| 52961354 | 2 | chr7 | 52961281 | 52961581 | *Dbp* | CRY2 | 129S6 |
| 46720828 | 2 | chr19 | 46720721 | 46721040 | *Cyp17a1* | CRY2 | 129S6 |
| 46720829 | 2 | chr19 | 46720721 | 46721040 | *Cyp17a1* | CRY2 | 129S6 |
| 46720943 | 2 | chr19 | 46720721 | 46721040 | *Cyp17a1* | CRY2 | 129S6 |
| 46716220 | 2 | chr19 | 46716111 | 46716590 | *Cyp17a1* | CRY2 | 129S6 |
| 68908614 | 2 | chr11 | 68908481 | 68908800 | *Per1* | NPAS2 | 129S6 |
| 68908620 | 2 | chr11 | 68908481 | 68908800 | *Per1* | NPAS2 | 129S6 |
| 68908750 | 2 | chr11 | 68908481 | 68908800 | *Per1* | NPAS2 | 129S6 |
| 68908310 | 2 | chr11 | 68908221 | 68908471 | *Per1* | NPAS2 | 129S6 |
| 98637109 | 2 | chr11 | 98636601 | 98637151 | *Nr1d1* | NPAS2 | 129S6 |
| 98644843 | 2 | chr11 | 98644621 | 98645190 | *Nr1d1* | NPAS2 | 129S6 |
| 98644918 | 2 | chr11 | 98644621 | 98645190 | *Nr1d1* | NPAS2 | 129S6 |
| 98637109 | 2 | chr11 | 98636841 | 98637150 | *Nr1d1* | NPAS2 | 129S6 |
| 98644918 | 2 | chr11 | 98644871 | 98645040 | *Nr1d1* | NPAS2 | 129S6 |
| 98644508 | 2 | chr11 | 98644481 | 98644871 | *Nr1d1* | NPAS2 | 129S6 |
| 98644565 | 2 | chr11 | 98644481 | 98644871 | *Nr1d1* | NPAS2 | 129S6 |
| 98644843 | 2 | chr11 | 98644481 | 98644871 | *Nr1d1* | NPAS2 | 129S6 |
| 93355989 | 2 | chr1 | 93355851 | 93356161 | *Per2* | NPAS2 | 129S6 |
| 93356260 | 2 | chr1 | 93356201 | 93356420 | *Per2* | NPAS2 | 129S6 |
| 93356397 | 2 | chr1 | 93356201 | 93356420 | *Per2* | NPAS2 | 129S6 |
| 52960092 | 2 | chr7 | 52960041 | 52960561 | *Dbp* | NPAS2 | 129S6 |
| 52960117 | 2 | chr7 | 52960041 | 52960561 | *Dbp* | NPAS2 | 129S6 |
| 52960169 | 2 | chr7 | 52960041 | 52960561 | *Dbp* | NPAS2 | 129S6 |
| 52960311 | 2 | chr7 | 52960041 | 52960561 | *Dbp* | NPAS2 | 129S6 |
| 52960312 | 2 | chr7 | 52960041 | 52960561 | *Dbp* | NPAS2 | 129S6 |
| 52960341 | 2 | chr7 | 52960041 | 52960561 | *Dbp* | NPAS2 | 129S6 |
| 52961354 | 2 | chr7 | 52961241 | 52961601 | *Dbp* | NPAS2 | 129S6 |
| 52963101 | 2 | chr7 | 52963061 | 52963370 | *Dbp* | NPAS2 | 129S6 |
| 52963127 | 2 | chr7 | 52963061 | 52963370 | *Dbp* | NPAS2 | 129S6 |
| 52963230 | 2 | chr7 | 52963061 | 52963370 | *Dbp* | NPAS2 | 129S6 |
| 52963234 | 2 | chr7 | 52963061 | 52963370 | *Dbp* | NPAS2 | 129S6 |
| 52963367 | 2 | chr7 | 52963061 | 52963370 | *Dbp* | NPAS2 | 129S6 |
| 52963542 | 2 | chr7 | 52963541 | 52963900 | *Dbp* | NPAS2 | 129S6 |
| 68908310 | 2 | chr11 | 68908211 | 68908501 | *Per1* | BMAL1 | 129T2 |
| 68908614 | 2 | chr11 | 68908511 | 68908830 | *Per1* | BMAL1 | 129T2 |
| 68908620 | 2 | chr11 | 68908511 | 68908830 | *Per1* | BMAL1 | 129T2 |
| 68908750 | 2 | chr11 | 68908511 | 68908830 | *Per1* | BMAL1 | 129T2 |
| 68908813 | 2 | chr11 | 68908511 | 68908830 | *Per1* | BMAL1 | 129T2 |
| 98644843 | 2 | chr11 | 98644571 | 98645021 | *Nr1d1* | BMAL1 | 129T2 |
| 98644918 | 2 | chr11 | 98644571 | 98645021 | *Nr1d1* | BMAL1 | 129T2 |
| 98637109 | 2 | chr11 | 98636841 | 98637181 | *Nr1d1* | BMAL1 | 129T2 |
| 98634168 | 2 | chr11 | 98634141 | 98634561 | *Nr1d1* | BMAL1 | 129T2 |
| 98643374 | 2 | chr11 | 98643181 | 98643480 | *Nr1d1* | BMAL1 | 129T2 |
| 98643452 | 2 | chr11 | 98643181 | 98643480 | *Nr1d1* | BMAL1 | 129T2 |
| 98643456 | 1 | chr11 | 98643181 | 98643480 | *Nr1d1* | BMAL1 | 129T2 |
| 93355989 | 2 | chr1 | 93355801 | 93356370 | *Per2* | BMAL1 | 129T2 |
| 93356260 | 2 | chr1 | 93355801 | 93356370 | *Per2* | BMAL1 | 129T2 |
| 93356260 | 2 | chr1 | 93356181 | 93356600 | *Per2* | BMAL1 | 129T2 |
| 93356397 | 2 | chr1 | 93356181 | 93356600 | *Per2* | BMAL1 | 129T2 |
| 150419182 | 2 | chr4 | 150418941 | 150419210 | *Per3* | BMAL1 | 129T2 |
| 150418904 | 2 | chr4 | 150418731 | 150418911 | *Per3* | BMAL1 | 129T2 |
| 27480259 | 2 | chr2 | 27480231 | 27480580 | *Rxra* | BMAL1 | 129T2 |
| 52960092 | 2 | chr7 | 52960041 | 52960760 | *Dbp* | BMAL1 | 129T2 |
| 52960117 | 2 | chr7 | 52960041 | 52960760 | *Dbp* | BMAL1 | 129T2 |
| 52960169 | 2 | chr7 | 52960041 | 52960760 | *Dbp* | BMAL1 | 129T2 |
| 52960311 | 2 | chr7 | 52960041 | 52960760 | *Dbp* | BMAL1 | 129T2 |
| 52960312 | 2 | chr7 | 52960041 | 52960760 | *Dbp* | BMAL1 | 129T2 |
| 52960341 | 2 | chr7 | 52960041 | 52960760 | *Dbp* | BMAL1 | 129T2 |
| 52960671 | 2 | chr7 | 52960041 | 52960760 | *Dbp* | BMAL1 | 129T2 |
| 52962826 | 2 | chr7 | 52962811 | 52963430 | *Dbp* | BMAL1 | 129T2 |
| 52963101 | 2 | chr7 | 52962811 | 52963430 | *Dbp* | BMAL1 | 129T2 |
| 52963127 | 2 | chr7 | 52962811 | 52963430 | *Dbp* | BMAL1 | 129T2 |
| 52963230 | 2 | chr7 | 52962811 | 52963430 | *Dbp* | BMAL1 | 129T2 |
| 52963234 | 2 | chr7 | 52962811 | 52963430 | *Dbp* | BMAL1 | 129T2 |
| 52963367 | 2 | chr7 | 52962811 | 52963430 | *Dbp* | BMAL1 | 129T2 |
| 52963415 | 2 | chr7 | 52962811 | 52963430 | *Dbp* | BMAL1 | 129T2 |
| 52963419 | 2 | chr7 | 52962811 | 52963430 | *Dbp* | BMAL1 | 129T2 |
| 52961354 | 2 | chr7 | 52961201 | 52961730 | *Dbp* | BMAL1 | 129T2 |
| 52961679 | 2 | chr7 | 52961201 | 52961730 | *Dbp* | BMAL1 | 129T2 |
| 52961720 | 2 | chr7 | 52961201 | 52961730 | *Dbp* | BMAL1 | 129T2 |
| 46716220 | 2 | chr19 | 46716101 | 46716281 | *Cyp17a1* | BMAL1 | 129T2 |
| 51943955 | 2 | chr5 | 51943791 | 51944090 | *Ppargc1a* | BMAL1 | 129T2 |
| 68908614 | 2 | chr11 | 68908511 | 68908970 | *Per1* | CLOCK | 129T2 |
| 68908620 | 2 | chr11 | 68908511 | 68908970 | *Per1* | CLOCK | 129T2 |
| 68908750 | 2 | chr11 | 68908511 | 68908970 | *Per1* | CLOCK | 129T2 |
| 68908813 | 2 | chr11 | 68908511 | 68908970 | *Per1* | CLOCK | 129T2 |
| 68908960 | 2 | chr11 | 68908511 | 68908970 | *Per1* | CLOCK | 129T2 |
| 68908968 | 2 | chr11 | 68908511 | 68908970 | *Per1* | CLOCK | 129T2 |
| 68908310 | 2 | chr11 | 68908231 | 68908501 | *Per1* | CLOCK | 129T2 |
| 98637109 | 2 | chr11 | 98636441 | 98637151 | *Nr1d1* | CLOCK | 129T2 |
| 98644565 | 2 | chr11 | 98644521 | 98645140 | *Nr1d1* | CLOCK | 129T2 |
| 98644843 | 2 | chr11 | 98644521 | 98645140 | *Nr1d1* | CLOCK | 129T2 |
| 98644918 | 2 | chr11 | 98644521 | 98645140 | *Nr1d1* | CLOCK | 129T2 |
| 98644508 | 2 | chr11 | 98644421 | 98644671 | *Nr1d1* | CLOCK | 129T2 |
| 98644565 | 2 | chr11 | 98644421 | 98644671 | *Nr1d1* | CLOCK | 129T2 |
| 93355989 | 2 | chr1 | 93355841 | 93356161 | *Per2* | CLOCK | 129T2 |
| 93356260 | 2 | chr1 | 93356221 | 93356440 | *Per2* | CLOCK | 129T2 |
| 93356397 | 2 | chr1 | 93356221 | 93356440 | *Per2* | CLOCK | 129T2 |
| 52960092 | 2 | chr7 | 52960041 | 52960800 | *Dbp* | CLOCK | 129T2 |
| 52960117 | 2 | chr7 | 52960041 | 52960800 | *Dbp* | CLOCK | 129T2 |
| 52960169 | 2 | chr7 | 52960041 | 52960800 | *Dbp* | CLOCK | 129T2 |
| 52960311 | 2 | chr7 | 52960041 | 52960800 | *Dbp* | CLOCK | 129T2 |
| 52960312 | 2 | chr7 | 52960041 | 52960800 | *Dbp* | CLOCK | 129T2 |
| 52960341 | 2 | chr7 | 52960041 | 52960800 | *Dbp* | CLOCK | 129T2 |
| 52960671 | 2 | chr7 | 52960041 | 52960800 | *Dbp* | CLOCK | 129T2 |
| 52961354 | 2 | chr7 | 52961331 | 52961720 | *Dbp* | CLOCK | 129T2 |
| 52961679 | 2 | chr7 | 52961331 | 52961720 | *Dbp* | CLOCK | 129T2 |
| 52963101 | 2 | chr7 | 52962911 | 52963280 | *Dbp* | CLOCK | 129T2 |
| 52963127 | 2 | chr7 | 52962911 | 52963280 | *Dbp* | CLOCK | 129T2 |
| 52963230 | 2 | chr7 | 52962911 | 52963280 | *Dbp* | CLOCK | 129T2 |
| 52963234 | 2 | chr7 | 52962911 | 52963280 | *Dbp* | CLOCK | 129T2 |
| 52960092 | 2 | chr7 | 52960071 | 52960211 | *Dbp* | CLOCK | 129T2 |
| 52960117 | 2 | chr7 | 52960071 | 52960211 | *Dbp* | CLOCK | 129T2 |
| 52960169 | 2 | chr7 | 52960071 | 52960211 | *Dbp* | CLOCK | 129T2 |
| 52960671 | 2 | chr7 | 52960581 | 52960840 | *Dbp* | CLOCK | 129T2 |
| 46716220 | 2 | chr19 | 46716061 | 46716420 | *Cyp17a1* | CLOCK | 129T2 |
| 51943736 | 2 | chr5 | 51943691 | 51944020 | *Ppargc1a* | CLOCK | 129T2 |
| 51943955 | 2 | chr5 | 51943691 | 51944020 | *Ppargc1a* | CLOCK | 129T2 |
| 68908614 | 2 | chr11 | 68908501 | 68908880 | *Per1* | PER1 | 129T2 |
| 68908620 | 2 | chr11 | 68908501 | 68908880 | *Per1* | PER1 | 129T2 |
| 68908750 | 2 | chr11 | 68908501 | 68908880 | *Per1* | PER1 | 129T2 |
| 68908813 | 2 | chr11 | 68908501 | 68908880 | *Per1* | PER1 | 129T2 |
| 68908310 | 2 | chr11 | 68908231 | 68908471 | *Per1* | PER1 | 129T2 |
| 98637109 | 2 | chr11 | 98636511 | 98637140 | *Nr1d1* | PER1 | 129T2 |
| 98644508 | 2 | chr11 | 98644481 | 98645140 | *Nr1d1* | PER1 | 129T2 |
| 98644565 | 2 | chr11 | 98644481 | 98645140 | *Nr1d1* | PER1 | 129T2 |
| 98644843 | 2 | chr11 | 98644481 | 98645140 | *Nr1d1* | PER1 | 129T2 |
| 98644918 | 2 | chr11 | 98644481 | 98645140 | *Nr1d1* | PER1 | 129T2 |
| 93355989 | 2 | chr1 | 93355871 | 93356151 | *Per2* | PER1 | 129T2 |
| 52960092 | 2 | chr7 | 52960021 | 52960541 | *Dbp* | PER1 | 129T2 |
| 52960117 | 2 | chr7 | 52960021 | 52960541 | *Dbp* | PER1 | 129T2 |
| 52960169 | 2 | chr7 | 52960021 | 52960541 | *Dbp* | PER1 | 129T2 |
| 52960311 | 2 | chr7 | 52960021 | 52960541 | *Dbp* | PER1 | 129T2 |
| 52960312 | 2 | chr7 | 52960021 | 52960541 | *Dbp* | PER1 | 129T2 |
| 52960341 | 2 | chr7 | 52960021 | 52960541 | *Dbp* | PER1 | 129T2 |
| 52963101 | 2 | chr7 | 52963041 | 52963300 | *Dbp* | PER1 | 129T2 |
| 52963127 | 2 | chr7 | 52963041 | 52963300 | *Dbp* | PER1 | 129T2 |
| 52963230 | 2 | chr7 | 52963041 | 52963300 | *Dbp* | PER1 | 129T2 |
| 52963234 | 2 | chr7 | 52963041 | 52963300 | *Dbp* | PER1 | 129T2 |
| 52961354 | 2 | chr7 | 52961341 | 52961610 | *Dbp* | PER1 | 129T2 |
| 46716220 | 2 | chr19 | 46716091 | 46716440 | *Cyp17a1* | PER1 | 129T2 |
| 46720943 | 2 | chr19 | 46720841 | 46721100 | *Cyp17a1* | PER1 | 129T2 |
| 68908614 | 2 | chr11 | 68908521 | 68908990 | *Per1* | PER2 | 129T2 |
| 68908620 | 2 | chr11 | 68908521 | 68908990 | *Per1* | PER2 | 129T2 |
| 68908750 | 2 | chr11 | 68908521 | 68908990 | *Per1* | PER2 | 129T2 |
| 68908813 | 2 | chr11 | 68908521 | 68908990 | *Per1* | PER2 | 129T2 |
| 68908960 | 2 | chr11 | 68908521 | 68908990 | *Per1* | PER2 | 129T2 |
| 68908968 | 2 | chr11 | 68908521 | 68908990 | *Per1* | PER2 | 129T2 |
| 68908310 | 2 | chr11 | 68908161 | 68908531 | *Per1* | PER2 | 129T2 |
| 98637109 | 2 | chr11 | 98636471 | 98637170 | *Nr1d1* | PER2 | 129T2 |
| 98644843 | 2 | chr11 | 98644661 | 98645150 | *Nr1d1* | PER2 | 129T2 |
| 98644918 | 2 | chr11 | 98644661 | 98645150 | *Nr1d1* | PER2 | 129T2 |
| 98634168 | 2 | chr11 | 98634141 | 98634530 | *Nr1d1* | PER2 | 129T2 |
| 98644508 | 2 | chr11 | 98644501 | 98644651 | *Nr1d1* | PER2 | 129T2 |
| 98644565 | 2 | chr11 | 98644501 | 98644651 | *Nr1d1* | PER2 | 129T2 |
| 93355989 | 2 | chr1 | 93355831 | 93356161 | *Per2* | PER2 | 129T2 |
| 93356260 | 2 | chr1 | 93356201 | 93356470 | *Per2* | PER2 | 129T2 |
| 93356397 | 2 | chr1 | 93356201 | 93356470 | *Per2* | PER2 | 129T2 |
| 93333537 | 2 | chr1 | 93333251 | 93333610 | *Per2* | PER2 | 129T2 |
| 150419182 | 2 | chr4 | 150418961 | 150419360 | *Per3* | PER2 | 129T2 |
| 150419349 | 2 | chr4 | 150418961 | 150419360 | *Per3* | PER2 | 129T2 |
| 150417652 | 2 | chr4 | 150417461 | 150417711 | *Per3* | PER2 | 129T2 |
| 52960092 | 2 | chr7 | 52960051 | 52960661 | *Dbp* | PER2 | 129T2 |
| 52960117 | 2 | chr7 | 52960051 | 52960661 | *Dbp* | PER2 | 129T2 |
| 52960169 | 2 | chr7 | 52960051 | 52960661 | *Dbp* | PER2 | 129T2 |
| 52960311 | 2 | chr7 | 52960051 | 52960661 | *Dbp* | PER2 | 129T2 |
| 52960312 | 2 | chr7 | 52960051 | 52960661 | *Dbp* | PER2 | 129T2 |
| 52960341 | 2 | chr7 | 52960051 | 52960661 | *Dbp* | PER2 | 129T2 |
| 52963101 | 2 | chr7 | 52962941 | 52963510 | *Dbp* | PER2 | 129T2 |
| 52963127 | 2 | chr7 | 52962941 | 52963510 | *Dbp* | PER2 | 129T2 |
| 52963230 | 2 | chr7 | 52962941 | 52963510 | *Dbp* | PER2 | 129T2 |
| 52963234 | 2 | chr7 | 52962941 | 52963510 | *Dbp* | PER2 | 129T2 |
| 52963367 | 2 | chr7 | 52962941 | 52963510 | *Dbp* | PER2 | 129T2 |
| 52963415 | 2 | chr7 | 52962941 | 52963510 | *Dbp* | PER2 | 129T2 |
| 52963419 | 2 | chr7 | 52962941 | 52963510 | *Dbp* | PER2 | 129T2 |
| 52961354 | 2 | chr7 | 52961311 | 52961650 | *Dbp* | PER2 | 129T2 |
| 46720828 | 2 | chr19 | 46720731 | 46721050 | *Cyp17a1* | PER2 | 129T2 |
| 46720829 | 2 | chr19 | 46720731 | 46721050 | *Cyp17a1* | PER2 | 129T2 |
| 46720943 | 2 | chr19 | 46720731 | 46721050 | *Cyp17a1* | PER2 | 129T2 |
| 46716220 | 2 | chr19 | 46716131 | 46716430 | *Cyp17a1* | PER2 | 129T2 |
| 120350590 | 2 | chr7 | 120350461 | 120351050 | *Arntl* | CRY1 | 129T2 |
| 52960092 | 2 | chr7 | 52959971 | 52960760 | *Dbp* | CRY1 | 129T2 |
| 52960117 | 2 | chr7 | 52959971 | 52960760 | *Dbp* | CRY1 | 129T2 |
| 52960169 | 2 | chr7 | 52959971 | 52960760 | *Dbp* | CRY1 | 129T2 |
| 52960311 | 2 | chr7 | 52959971 | 52960760 | *Dbp* | CRY1 | 129T2 |
| 52960312 | 2 | chr7 | 52959971 | 52960760 | *Dbp* | CRY1 | 129T2 |
| 52960341 | 2 | chr7 | 52959971 | 52960760 | *Dbp* | CRY1 | 129T2 |
| 52960671 | 2 | chr7 | 52959971 | 52960760 | *Dbp* | CRY1 | 129T2 |
| 52963101 | 2 | chr7 | 52962941 | 52963361 | *Dbp* | CRY1 | 129T2 |
| 52963127 | 2 | chr7 | 52962941 | 52963361 | *Dbp* | CRY1 | 129T2 |
| 52963230 | 2 | chr7 | 52962941 | 52963361 | *Dbp* | CRY1 | 129T2 |
| 52963234 | 2 | chr7 | 52962941 | 52963361 | *Dbp* | CRY1 | 129T2 |
| 52961354 | 2 | chr7 | 52961341 | 52961720 | *Dbp* | CRY1 | 129T2 |
| 52961679 | 2 | chr7 | 52961341 | 52961720 | *Dbp* | CRY1 | 129T2 |
| 52963415 | 2 | chr7 | 52963371 | 52963971 | *Dbp* | CRY1 | 129T2 |
| 52963419 | 2 | chr7 | 52963371 | 52963971 | *Dbp* | CRY1 | 129T2 |
| 52963542 | 2 | chr7 | 52963371 | 52963971 | *Dbp* | CRY1 | 129T2 |
| 52960671 | 2 | chr7 | 52960521 | 52960701 | *Dbp* | CRY1 | 129T2 |
| 52959755 | 2 | chr7 | 52959651 | 52959951 | *Dbp* | CRY1 | 129T2 |
| 52959760 | 2 | chr7 | 52959651 | 52959951 | *Dbp* | CRY1 | 129T2 |
| 52959795 | 2 | chr7 | 52959651 | 52959951 | *Dbp* | CRY1 | 129T2 |
| 52959486 | 2 | chr7 | 52959301 | 52959631 | *Dbp* | CRY1 | 129T2 |
| 52959594 | 2 | chr7 | 52959301 | 52959631 | *Dbp* | CRY1 | 129T2 |
| 52959619 | 2 | chr7 | 52959301 | 52959631 | *Dbp* | CRY1 | 129T2 |
| 68908310 | 2 | chr11 | 68907811 | 68908521 | *Per1* | CRY1 | 129T2 |
| 68908614 | 2 | chr11 | 68908531 | 68908941 | *Per1* | CRY1 | 129T2 |
| 68908620 | 2 | chr11 | 68908531 | 68908941 | *Per1* | CRY1 | 129T2 |
| 68908750 | 2 | chr11 | 68908531 | 68908941 | *Per1* | CRY1 | 129T2 |
| 68908813 | 2 | chr11 | 68908531 | 68908941 | *Per1* | CRY1 | 129T2 |
| 98637109 | 2 | chr11 | 98636401 | 98637420 | *Nr1d1* | CRY1 | 129T2 |
| 98644565 | 2 | chr11 | 98644521 | 98645050 | *Nr1d1* | CRY1 | 129T2 |
| 98644843 | 2 | chr11 | 98644521 | 98645050 | *Nr1d1* | CRY1 | 129T2 |
| 98644918 | 2 | chr11 | 98644521 | 98645050 | *Nr1d1* | CRY1 | 129T2 |
| 98634168 | 2 | chr11 | 98634161 | 98634611 | *Nr1d1* | CRY1 | 129T2 |
| 98644508 | 2 | chr11 | 98644481 | 98644651 | *Nr1d1* | CRY1 | 129T2 |
| 98644565 | 2 | chr11 | 98644481 | 98644651 | *Nr1d1* | CRY1 | 129T2 |
| 98636167 | 2 | chr11 | 98635971 | 98636171 | *Nr1d1* | CRY1 | 129T2 |
| 93355989 | 2 | chr1 | 93355741 | 93356191 | *Per2* | CRY1 | 129T2 |
| 93356260 | 2 | chr1 | 93356201 | 93356490 | *Per2* | CRY1 | 129T2 |
| 93356397 | 2 | chr1 | 93356201 | 93356490 | *Per2* | CRY1 | 129T2 |
| 93353564 | 2 | chr1 | 93353041 | 93353620 | *Per2* | CRY1 | 129T2 |
| 93352816 | 2 | chr1 | 93352511 | 93352900 | *Per2* | CRY1 | 129T2 |
| 93337174 | 2 | chr1 | 93336811 | 93337230 | *Per2* | CRY1 | 129T2 |
| 93337211 | 2 | chr1 | 93336811 | 93337230 | *Per2* | CRY1 | 129T2 |
| 93333537 | 2 | chr1 | 93333191 | 93333720 | *Per2* | CRY1 | 129T2 |
| 150418904 | 2 | chr4 | 150418811 | 150419271 | *Per3* | CRY1 | 129T2 |
| 150419182 | 2 | chr4 | 150418811 | 150419271 | *Per3* | CRY1 | 129T2 |
| 150417652 | 2 | chr4 | 150417501 | 150417850 | *Per3* | CRY1 | 129T2 |
| 150417822 | 2 | chr4 | 150417501 | 150417850 | *Per3* | CRY1 | 129T2 |
| 27490820 | 2 | chr2 | 27490731 | 27491100 | *Rxra* | CRY1 | 129T2 |
| 27490957 | 2 | chr2 | 27490731 | 27491100 | *Rxra* | CRY1 | 129T2 |
| 27490970 | 2 | chr2 | 27490731 | 27491100 | *Rxra* | CRY1 | 129T2 |
| 27490972 | 2 | chr2 | 27490731 | 27491100 | *Rxra* | CRY1 | 129T2 |
| 27490974 | 2 | chr2 | 27490731 | 27491100 | *Rxra* | CRY1 | 129T2 |
| 18068970 | 2 | chr3 | 18068881 | 18069180 | *Cyp7b1* | CRY1 | 129T2 |
| 18151353 | 2 | chr3 | 18151181 | 18151490 | *Cyp7b1* | CRY1 | 129T2 |
| 46716220 | 2 | chr19 | 46716091 | 46716450 | *Cyp17a1* | CRY1 | 129T2 |
| 46720828 | 2 | chr19 | 46720771 | 46721040 | *Cyp17a1* | CRY1 | 129T2 |
| 46720829 | 2 | chr19 | 46720771 | 46721040 | *Cyp17a1* | CRY1 | 129T2 |
| 46720943 | 2 | chr19 | 46720771 | 46721040 | *Cyp17a1* | CRY1 | 129T2 |
| 68908614 | 2 | chr11 | 68908521 | 68908920 | *Per1* | CRY2 | 129T2 |
| 68908620 | 2 | chr11 | 68908521 | 68908920 | *Per1* | CRY2 | 129T2 |
| 68908750 | 2 | chr11 | 68908521 | 68908920 | *Per1* | CRY2 | 129T2 |
| 68908813 | 2 | chr11 | 68908521 | 68908920 | *Per1* | CRY2 | 129T2 |
| 68908310 | 2 | chr11 | 68908221 | 68908511 | *Per1* | CRY2 | 129T2 |
| 98637109 | 2 | chr11 | 98636431 | 98637110 | *Nr1d1* | CRY2 | 129T2 |
| 98644843 | 2 | chr11 | 98644651 | 98645060 | *Nr1d1* | CRY2 | 129T2 |
| 98644918 | 2 | chr11 | 98644651 | 98645060 | *Nr1d1* | CRY2 | 129T2 |
| 98634168 | 2 | chr11 | 98634151 | 98634541 | *Nr1d1* | CRY2 | 129T2 |
| 98637109 | 2 | chr11 | 98636911 | 98637150 | *Nr1d1* | CRY2 | 129T2 |
| 98644508 | 2 | chr11 | 98644471 | 98644661 | *Nr1d1* | CRY2 | 129T2 |
| 98644565 | 2 | chr11 | 98644471 | 98644661 | *Nr1d1* | CRY2 | 129T2 |
| 93355989 | 2 | chr1 | 93355861 | 93356171 | *Per2* | CRY2 | 129T2 |
| 93356260 | 2 | chr1 | 93356181 | 93356411 | *Per2* | CRY2 | 129T2 |
| 93356397 | 2 | chr1 | 93356181 | 93356411 | *Per2* | CRY2 | 129T2 |
| 150419182 | 2 | chr4 | 150418961 | 150419191 | *Per3* | CRY2 | 129T2 |
| 27482311 | 2 | chr2 | 27482231 | 27482470 | *Rxra* | CRY2 | 129T2 |
| 27482313 | 2 | chr2 | 27482231 | 27482470 | *Rxra* | CRY2 | 129T2 |
| 27482370 | 2 | chr2 | 27482231 | 27482470 | *Rxra* | CRY2 | 129T2 |
| 52960092 | 2 | chr7 | 52959961 | 52960611 | *Dbp* | CRY2 | 129T2 |
| 52960117 | 2 | chr7 | 52959961 | 52960611 | *Dbp* | CRY2 | 129T2 |
| 52960169 | 2 | chr7 | 52959961 | 52960611 | *Dbp* | CRY2 | 129T2 |
| 52960311 | 2 | chr7 | 52959961 | 52960611 | *Dbp* | CRY2 | 129T2 |
| 52960312 | 2 | chr7 | 52959961 | 52960611 | *Dbp* | CRY2 | 129T2 |
| 52960341 | 2 | chr7 | 52959961 | 52960611 | *Dbp* | CRY2 | 129T2 |
| 52963101 | 2 | chr7 | 52962841 | 52963420 | *Dbp* | CRY2 | 129T2 |
| 52963127 | 2 | chr7 | 52962841 | 52963420 | *Dbp* | CRY2 | 129T2 |
| 52963230 | 2 | chr7 | 52962841 | 52963420 | *Dbp* | CRY2 | 129T2 |
| 52963234 | 2 | chr7 | 52962841 | 52963420 | *Dbp* | CRY2 | 129T2 |
| 52963367 | 2 | chr7 | 52962841 | 52963420 | *Dbp* | CRY2 | 129T2 |
| 52963415 | 2 | chr7 | 52962841 | 52963420 | *Dbp* | CRY2 | 129T2 |
| 52963419 | 2 | chr7 | 52962841 | 52963420 | *Dbp* | CRY2 | 129T2 |
| 52960671 | 2 | chr7 | 52960481 | 52960721 | *Dbp* | CRY2 | 129T2 |
| 52961354 | 2 | chr7 | 52961281 | 52961581 | *Dbp* | CRY2 | 129T2 |
| 46720828 | 2 | chr19 | 46720721 | 46721040 | *Cyp17a1* | CRY2 | 129T2 |
| 46720829 | 2 | chr19 | 46720721 | 46721040 | *Cyp17a1* | CRY2 | 129T2 |
| 46720943 | 2 | chr19 | 46720721 | 46721040 | *Cyp17a1* | CRY2 | 129T2 |
| 46716220 | 2 | chr19 | 46716111 | 46716590 | *Cyp17a1* | CRY2 | 129T2 |
| 68908614 | 2 | chr11 | 68908481 | 68908800 | *Per1* | NPAS2 | 129T2 |
| 68908620 | 2 | chr11 | 68908481 | 68908800 | *Per1* | NPAS2 | 129T2 |
| 68908750 | 2 | chr11 | 68908481 | 68908800 | *Per1* | NPAS2 | 129T2 |
| 68908310 | 2 | chr11 | 68908221 | 68908471 | *Per1* | NPAS2 | 129T2 |
| 98637109 | 2 | chr11 | 98636601 | 98637151 | *Nr1d1* | NPAS2 | 129T2 |
| 98644843 | 2 | chr11 | 98644621 | 98645190 | *Nr1d1* | NPAS2 | 129T2 |
| 98644918 | 2 | chr11 | 98644621 | 98645190 | *Nr1d1* | NPAS2 | 129T2 |
| 98637109 | 2 | chr11 | 98636841 | 98637150 | *Nr1d1* | NPAS2 | 129T2 |
| 98644918 | 2 | chr11 | 98644871 | 98645040 | *Nr1d1* | NPAS2 | 129T2 |
| 98644508 | 2 | chr11 | 98644481 | 98644871 | *Nr1d1* | NPAS2 | 129T2 |
| 98644565 | 2 | chr11 | 98644481 | 98644871 | *Nr1d1* | NPAS2 | 129T2 |
| 98644843 | 2 | chr11 | 98644481 | 98644871 | *Nr1d1* | NPAS2 | 129T2 |
| 93355989 | 2 | chr1 | 93355851 | 93356161 | *Per2* | NPAS2 | 129T2 |
| 93356260 | 2 | chr1 | 93356201 | 93356420 | *Per2* | NPAS2 | 129T2 |
| 93356397 | 2 | chr1 | 93356201 | 93356420 | *Per2* | NPAS2 | 129T2 |
| 52960092 | 2 | chr7 | 52960041 | 52960561 | *Dbp* | NPAS2 | 129T2 |
| 52960117 | 2 | chr7 | 52960041 | 52960561 | *Dbp* | NPAS2 | 129T2 |
| 52960169 | 2 | chr7 | 52960041 | 52960561 | *Dbp* | NPAS2 | 129T2 |
| 52960311 | 2 | chr7 | 52960041 | 52960561 | *Dbp* | NPAS2 | 129T2 |
| 52960312 | 2 | chr7 | 52960041 | 52960561 | *Dbp* | NPAS2 | 129T2 |
| 52960341 | 2 | chr7 | 52960041 | 52960561 | *Dbp* | NPAS2 | 129T2 |
| 52961354 | 2 | chr7 | 52961241 | 52961601 | *Dbp* | NPAS2 | 129T2 |
| 52963101 | 2 | chr7 | 52963061 | 52963370 | *Dbp* | NPAS2 | 129T2 |
| 52963127 | 2 | chr7 | 52963061 | 52963370 | *Dbp* | NPAS2 | 129T2 |
| 52963230 | 2 | chr7 | 52963061 | 52963370 | *Dbp* | NPAS2 | 129T2 |
| 52963234 | 2 | chr7 | 52963061 | 52963370 | *Dbp* | NPAS2 | 129T2 |
| 52963367 | 2 | chr7 | 52963061 | 52963370 | *Dbp* | NPAS2 | 129T2 |
| 52963542 | 2 | chr7 | 52963541 | 52963900 | *Dbp* | NPAS2 | 129T2 |
| 68908310 | 2 | chr11 | 68908211 | 68908501 | *Per1* | BMAL1 | 129X1 |
| 68908614 | 2 | chr11 | 68908511 | 68908830 | *Per1* | BMAL1 | 129X1 |
| 68908620 | 2 | chr11 | 68908511 | 68908830 | *Per1* | BMAL1 | 129X1 |
| 68908750 | 2 | chr11 | 68908511 | 68908830 | *Per1* | BMAL1 | 129X1 |
| 68908813 | 2 | chr11 | 68908511 | 68908830 | *Per1* | BMAL1 | 129X1 |
| 98644843 | 2 | chr11 | 98644571 | 98645021 | *Nr1d1* | BMAL1 | 129X1 |
| 98644918 | 2 | chr11 | 98644571 | 98645021 | *Nr1d1* | BMAL1 | 129X1 |
| 98637109 | 2 | chr11 | 98636841 | 98637181 | *Nr1d1* | BMAL1 | 129X1 |
| 98634168 | 2 | chr11 | 98634141 | 98634561 | *Nr1d1* | BMAL1 | 129X1 |
| 98643374 | 2 | chr11 | 98643181 | 98643480 | *Nr1d1* | BMAL1 | 129X1 |
| 98643452 | 2 | chr11 | 98643181 | 98643480 | *Nr1d1* | BMAL1 | 129X1 |
| 98643456 | 1 | chr11 | 98643181 | 98643480 | *Nr1d1* | BMAL1 | 129X1 |
| 93355989 | 2 | chr1 | 93355801 | 93356370 | *Per2* | BMAL1 | 129X1 |
| 93356260 | 2 | chr1 | 93355801 | 93356370 | *Per2* | BMAL1 | 129X1 |
| 93356260 | 2 | chr1 | 93356181 | 93356600 | *Per2* | BMAL1 | 129X1 |
| 93356397 | 2 | chr1 | 93356181 | 93356600 | *Per2* | BMAL1 | 129X1 |
| 150419182 | 2 | chr4 | 150418941 | 150419210 | *Per3* | BMAL1 | 129X1 |
| 150418904 | 2 | chr4 | 150418731 | 150418911 | *Per3* | BMAL1 | 129X1 |
| 27480259 | 2 | chr2 | 27480231 | 27480580 | *Rxra* | BMAL1 | 129X1 |
| 51943955 | 2 | chr5 | 51943791 | 51944090 | *Ppargc1a* | BMAL1 | 129X1 |
| 68908614 | 2 | chr11 | 68908511 | 68908970 | *Per1* | CLOCK | 129X1 |
| 68908620 | 2 | chr11 | 68908511 | 68908970 | *Per1* | CLOCK | 129X1 |
| 68908750 | 2 | chr11 | 68908511 | 68908970 | *Per1* | CLOCK | 129X1 |
| 68908813 | 2 | chr11 | 68908511 | 68908970 | *Per1* | CLOCK | 129X1 |
| 68908960 | 2 | chr11 | 68908511 | 68908970 | *Per1* | CLOCK | 129X1 |
| 68908968 | 2 | chr11 | 68908511 | 68908970 | *Per1* | CLOCK | 129X1 |
| 68908310 | 2 | chr11 | 68908231 | 68908501 | *Per1* | CLOCK | 129X1 |
| 98637109 | 2 | chr11 | 98636441 | 98637151 | *Nr1d1* | CLOCK | 129X1 |
| 98644565 | 2 | chr11 | 98644521 | 98645140 | *Nr1d1* | CLOCK | 129X1 |
| 98644843 | 2 | chr11 | 98644521 | 98645140 | *Nr1d1* | CLOCK | 129X1 |
| 98644918 | 2 | chr11 | 98644521 | 98645140 | *Nr1d1* | CLOCK | 129X1 |
| 98644508 | 2 | chr11 | 98644421 | 98644671 | *Nr1d1* | CLOCK | 129X1 |
| 98644565 | 2 | chr11 | 98644421 | 98644671 | *Nr1d1* | CLOCK | 129X1 |
| 93355989 | 2 | chr1 | 93355841 | 93356161 | *Per2* | CLOCK | 129X1 |
| 93356260 | 2 | chr1 | 93356221 | 93356440 | *Per2* | CLOCK | 129X1 |
| 93356397 | 2 | chr1 | 93356221 | 93356440 | *Per2* | CLOCK | 129X1 |
| 51943736 | 2 | chr5 | 51943691 | 51944020 | *Ppargc1a* | CLOCK | 129X1 |
| 51943955 | 2 | chr5 | 51943691 | 51944020 | *Ppargc1a* | CLOCK | 129X1 |
| 68908614 | 2 | chr11 | 68908501 | 68908880 | *Per1* | PER1 | 129X1 |
| 68908620 | 2 | chr11 | 68908501 | 68908880 | *Per1* | PER1 | 129X1 |
| 68908750 | 2 | chr11 | 68908501 | 68908880 | *Per1* | PER1 | 129X1 |
| 68908813 | 2 | chr11 | 68908501 | 68908880 | *Per1* | PER1 | 129X1 |
| 68908310 | 2 | chr11 | 68908231 | 68908471 | *Per1* | PER1 | 129X1 |
| 98637109 | 2 | chr11 | 98636511 | 98637140 | *Nr1d1* | PER1 | 129X1 |
| 98644508 | 2 | chr11 | 98644481 | 98645140 | *Nr1d1* | PER1 | 129X1 |
| 98644565 | 2 | chr11 | 98644481 | 98645140 | *Nr1d1* | PER1 | 129X1 |
| 98644843 | 2 | chr11 | 98644481 | 98645140 | *Nr1d1* | PER1 | 129X1 |
| 98644918 | 2 | chr11 | 98644481 | 98645140 | *Nr1d1* | PER1 | 129X1 |
| 93355989 | 2 | chr1 | 93355871 | 93356151 | *Per2* | PER1 | 129X1 |
| 68908614 | 2 | chr11 | 68908521 | 68908990 | *Per1* | PER2 | 129X1 |
| 68908620 | 2 | chr11 | 68908521 | 68908990 | *Per1* | PER2 | 129X1 |
| 68908750 | 2 | chr11 | 68908521 | 68908990 | *Per1* | PER2 | 129X1 |
| 68908813 | 2 | chr11 | 68908521 | 68908990 | *Per1* | PER2 | 129X1 |
| 68908960 | 2 | chr11 | 68908521 | 68908990 | *Per1* | PER2 | 129X1 |
| 68908968 | 2 | chr11 | 68908521 | 68908990 | *Per1* | PER2 | 129X1 |
| 68908310 | 2 | chr11 | 68908161 | 68908531 | *Per1* | PER2 | 129X1 |
| 98637109 | 2 | chr11 | 98636471 | 98637170 | *Nr1d1* | PER2 | 129X1 |
| 98644843 | 2 | chr11 | 98644661 | 98645150 | *Nr1d1* | PER2 | 129X1 |
| 98644918 | 2 | chr11 | 98644661 | 98645150 | *Nr1d1* | PER2 | 129X1 |
| 98634168 | 2 | chr11 | 98634141 | 98634530 | *Nr1d1* | PER2 | 129X1 |
| 98644508 | 2 | chr11 | 98644501 | 98644651 | *Nr1d1* | PER2 | 129X1 |
| 98644565 | 2 | chr11 | 98644501 | 98644651 | *Nr1d1* | PER2 | 129X1 |
| 93355989 | 2 | chr1 | 93355831 | 93356161 | *Per2* | PER2 | 129X1 |
| 93356260 | 2 | chr1 | 93356201 | 93356470 | *Per2* | PER2 | 129X1 |
| 93356397 | 2 | chr1 | 93356201 | 93356470 | *Per2* | PER2 | 129X1 |
| 93333537 | 2 | chr1 | 93333251 | 93333610 | *Per2* | PER2 | 129X1 |
| 150419182 | 2 | chr4 | 150418961 | 150419360 | *Per3* | PER2 | 129X1 |
| 150419349 | 2 | chr4 | 150418961 | 150419360 | *Per3* | PER2 | 129X1 |
| 150417652 | 2 | chr4 | 150417461 | 150417711 | *Per3* | PER2 | 129X1 |
| 84652894 | 2 | chr10 | 84652861 | 84653071 | *Cry1* | PER2 | 129X1 |
| 120350590 | 2 | chr7 | 120350461 | 120351050 | *Arntl* | CRY1 | 129X1 |
| 68908310 | 2 | chr11 | 68907811 | 68908521 | *Per1* | CRY1 | 129X1 |
| 68908614 | 2 | chr11 | 68908531 | 68908941 | *Per1* | CRY1 | 129X1 |
| 68908620 | 2 | chr11 | 68908531 | 68908941 | *Per1* | CRY1 | 129X1 |
| 68908750 | 2 | chr11 | 68908531 | 68908941 | *Per1* | CRY1 | 129X1 |
| 68908813 | 2 | chr11 | 68908531 | 68908941 | *Per1* | CRY1 | 129X1 |
| 98637109 | 2 | chr11 | 98636401 | 98637420 | *Nr1d1* | CRY1 | 129X1 |
| 98644565 | 2 | chr11 | 98644521 | 98645050 | *Nr1d1* | CRY1 | 129X1 |
| 98644843 | 2 | chr11 | 98644521 | 98645050 | *Nr1d1* | CRY1 | 129X1 |
| 98644918 | 2 | chr11 | 98644521 | 98645050 | *Nr1d1* | CRY1 | 129X1 |
| 98634168 | 2 | chr11 | 98634161 | 98634611 | *Nr1d1* | CRY1 | 129X1 |
| 98644508 | 2 | chr11 | 98644481 | 98644651 | *Nr1d1* | CRY1 | 129X1 |
| 98644565 | 2 | chr11 | 98644481 | 98644651 | *Nr1d1* | CRY1 | 129X1 |
| 98636167 | 2 | chr11 | 98635971 | 98636171 | *Nr1d1* | CRY1 | 129X1 |
| 93355989 | 2 | chr1 | 93355741 | 93356191 | *Per2* | CRY1 | 129X1 |
| 93356260 | 2 | chr1 | 93356201 | 93356490 | *Per2* | CRY1 | 129X1 |
| 93356397 | 2 | chr1 | 93356201 | 93356490 | *Per2* | CRY1 | 129X1 |
| 93353564 | 2 | chr1 | 93353041 | 93353620 | *Per2* | CRY1 | 129X1 |
| 93352816 | 2 | chr1 | 93352511 | 93352900 | *Per2* | CRY1 | 129X1 |
| 93337174 | 2 | chr1 | 93336811 | 93337230 | *Per2* | CRY1 | 129X1 |
| 93337211 | 2 | chr1 | 93336811 | 93337230 | *Per2* | CRY1 | 129X1 |
| 93333537 | 2 | chr1 | 93333191 | 93333720 | *Per2* | CRY1 | 129X1 |
| 150418904 | 2 | chr4 | 150418811 | 150419271 | *Per3* | CRY1 | 129X1 |
| 150419182 | 2 | chr4 | 150418811 | 150419271 | *Per3* | CRY1 | 129X1 |
| 150417652 | 2 | chr4 | 150417501 | 150417850 | *Per3* | CRY1 | 129X1 |
| 150417822 | 2 | chr4 | 150417501 | 150417850 | *Per3* | CRY1 | 129X1 |
| 84652797 | 2 | chr10 | 84652741 | 84653121 | *Cry1* | CRY1 | 129X1 |
| 84652855 | 2 | chr10 | 84652741 | 84653121 | *Cry1* | CRY1 | 129X1 |
| 84652856 | 2 | chr10 | 84652741 | 84653121 | *Cry1* | CRY1 | 129X1 |
| 84652894 | 2 | chr10 | 84652741 | 84653121 | *Cry1* | CRY1 | 129X1 |
| 27490820 | 2 | chr2 | 27490731 | 27491100 | *Rxra* | CRY1 | 129X1 |
| 27490957 | 2 | chr2 | 27490731 | 27491100 | *Rxra* | CRY1 | 129X1 |
| 27490970 | 2 | chr2 | 27490731 | 27491100 | *Rxra* | CRY1 | 129X1 |
| 27490972 | 2 | chr2 | 27490731 | 27491100 | *Rxra* | CRY1 | 129X1 |
| 27490974 | 2 | chr2 | 27490731 | 27491100 | *Rxra* | CRY1 | 129X1 |
| 18068970 | 2 | chr3 | 18068881 | 18069180 | *Cyp7b1* | CRY1 | 129X1 |
| 18151353 | 2 | chr3 | 18151181 | 18151490 | *Cyp7b1* | CRY1 | 129X1 |
| 68908614 | 2 | chr11 | 68908521 | 68908920 | *Per1* | CRY2 | 129X1 |
| 68908620 | 2 | chr11 | 68908521 | 68908920 | *Per1* | CRY2 | 129X1 |
| 68908750 | 2 | chr11 | 68908521 | 68908920 | *Per1* | CRY2 | 129X1 |
| 68908813 | 2 | chr11 | 68908521 | 68908920 | *Per1* | CRY2 | 129X1 |
| 68908310 | 2 | chr11 | 68908221 | 68908511 | *Per1* | CRY2 | 129X1 |
| 98637109 | 2 | chr11 | 98636431 | 98637110 | *Nr1d1* | CRY2 | 129X1 |
| 98644843 | 2 | chr11 | 98644651 | 98645060 | *Nr1d1* | CRY2 | 129X1 |
| 98644918 | 2 | chr11 | 98644651 | 98645060 | *Nr1d1* | CRY2 | 129X1 |
| 98634168 | 2 | chr11 | 98634151 | 98634541 | *Nr1d1* | CRY2 | 129X1 |
| 98637109 | 2 | chr11 | 98636911 | 98637150 | *Nr1d1* | CRY2 | 129X1 |
| 98644508 | 2 | chr11 | 98644471 | 98644661 | *Nr1d1* | CRY2 | 129X1 |
| 98644565 | 2 | chr11 | 98644471 | 98644661 | *Nr1d1* | CRY2 | 129X1 |
| 93355989 | 2 | chr1 | 93355861 | 93356171 | *Per2* | CRY2 | 129X1 |
| 93356260 | 2 | chr1 | 93356181 | 93356411 | *Per2* | CRY2 | 129X1 |
| 93356397 | 2 | chr1 | 93356181 | 93356411 | *Per2* | CRY2 | 129X1 |
| 150419182 | 2 | chr4 | 150418961 | 150419191 | *Per3* | CRY2 | 129X1 |
| 84652797 | 2 | chr10 | 84652791 | 84653140 | *Cry1* | CRY2 | 129X1 |
| 84652855 | 2 | chr10 | 84652791 | 84653140 | *Cry1* | CRY2 | 129X1 |
| 84652856 | 2 | chr10 | 84652791 | 84653140 | *Cry1* | CRY2 | 129X1 |
| 84652894 | 2 | chr10 | 84652791 | 84653140 | *Cry1* | CRY2 | 129X1 |
| 84653124 | 2 | chr10 | 84652791 | 84653140 | *Cry1* | CRY2 | 129X1 |
| 84653128 | 2 | chr10 | 84652791 | 84653140 | *Cry1* | CRY2 | 129X1 |
| 27482311 | 2 | chr2 | 27482231 | 27482470 | *Rxra* | CRY2 | 129X1 |
| 27482313 | 2 | chr2 | 27482231 | 27482470 | *Rxra* | CRY2 | 129X1 |
| 27482370 | 2 | chr2 | 27482231 | 27482470 | *Rxra* | CRY2 | 129X1 |
| 68908614 | 2 | chr11 | 68908481 | 68908800 | *Per1* | NPAS2 | 129X1 |
| 68908620 | 2 | chr11 | 68908481 | 68908800 | *Per1* | NPAS2 | 129X1 |
| 68908750 | 2 | chr11 | 68908481 | 68908800 | *Per1* | NPAS2 | 129X1 |
| 68908310 | 2 | chr11 | 68908221 | 68908471 | *Per1* | NPAS2 | 129X1 |
| 98637109 | 2 | chr11 | 98636601 | 98637151 | *Nr1d1* | NPAS2 | 129X1 |
| 98644843 | 2 | chr11 | 98644621 | 98645190 | *Nr1d1* | NPAS2 | 129X1 |
| 98644918 | 2 | chr11 | 98644621 | 98645190 | *Nr1d1* | NPAS2 | 129X1 |
| 98637109 | 2 | chr11 | 98636841 | 98637150 | *Nr1d1* | NPAS2 | 129X1 |
| 98644918 | 2 | chr11 | 98644871 | 98645040 | *Nr1d1* | NPAS2 | 129X1 |
| 98644508 | 2 | chr11 | 98644481 | 98644871 | *Nr1d1* | NPAS2 | 129X1 |
| 98644565 | 2 | chr11 | 98644481 | 98644871 | *Nr1d1* | NPAS2 | 129X1 |
| 98644843 | 2 | chr11 | 98644481 | 98644871 | *Nr1d1* | NPAS2 | 129X1 |
| 93355989 | 2 | chr1 | 93355851 | 93356161 | *Per2* | NPAS2 | 129X1 |
| 93356260 | 2 | chr1 | 93356201 | 93356420 | *Per2* | NPAS2 | 129X1 |
| 93356397 | 2 | chr1 | 93356201 | 93356420 | *Per2* | NPAS2 | 129X1 |

**Supplementary Table S11**

Analysis of SNPs from binding regions of clock core genes in open chromatin regions.

The table is appended as an additional Supplementary file with the name: **Table S11.xlsx**

## Supplementary References

1. Nantel, F. *et al.* Spermiogenesis deficiency and germ-cell apoptosis in CREM-mutant mice. *Nature* **380,** 159–162. (1996).

2. Kosir, R. *et al.* Determination of reference genes for circadian studies in different tissues and mouse strains. *BMC Mol Biol* **11,** 60 (2010).

3. Nelson, W., Tong, Y. L., Lee, J. K. & Halberg, F. Methods for cosinor-rhythmometry. *Chronobiologia* **6,** 305–23 (1979).

4. Wickham, H. *Ggplot2 : elegant graphics for data analysis*. (Springer, 2009). at <http://bvbr.bib-bvb.de:8991/F?func=service&doc_library=BVB01&doc_number=017387312&line_number=0001&func_code=DB_RECORDS&service_type=MEDIA>

5. Rudd, M. F. *et al.* The predicted impact of coding single nucleotide polymorphisms database. *Cancer Epidemiol Biomark. Prev* **14,** 2598–604 (2005).

6. Koike, N. *et al.* Transcriptional Architecture and Chromatin Landscape of the Core Circadian Clock in Mammals. *Science* **338,** 349–354 (2012).

7. Wang, J. R. *et al.* Imputation of single-nucleotide polymorphisms in inbred mice using local phylogeny. *Genetics* **190,** 449–58 (2012).

8. Wingender, E., Dietze, P., Karas, H. & Knüppel, R. TRANSFAC: A Database on Transcription Factors and Their DNA Binding Sites. *Nucleic Acids Res.* **24,** 238–241 (1996).
